# Photochemical reduction of aryl chlorides, bromides, and iodides *via* ternary EDA complexes with guanidine bases

**DOI:** 10.1039/d6sc00251j

**Published:** 2026-02-26

**Authors:** Robert J. Hannan, Alexandria M. Vondran, Sunghwan Cho, Kerry M. Hanson, Claire Tonnelé, David Casanova, Christopher J. Bardeen, Ana Bahamonde

**Affiliations:** a Address Chemistry Department, University of California, Riverside 501 Big Springs Rd. Riverside CA 92521 USA ana.bahamonde@ucr.edu; b Ikerbasque Researcher, Donostia International Physics Center (DIPC) Paseo Manuel Lardizabal 4 20018 Donostia Euskadi Spain

## Abstract

Photoredox catalysis has traditionally required sophisticated catalyst design, multi-photon systems, or photoelectrochemical strategies to reach the reducing potentials necessary for aryl chloride activation. In a surprising departure from these paradigms, we find that a simple, bench-stable guanidine base, TBD, functions as a powerful photoreductant under visible light, promoting the reduction of aryl iodides, bromides, and even unactivated aryl chlorides—as well as Birch-type dearomatization of polyarenes. Mechanistic interrogation reveals an unusual ternary EDA complex formed between a TBD dimer and the aryl halide, which upon excitation engages in an electron transfer, generating aryl radicals. These intermediates enable hydrodehalogenation, borylation, and radical cyclization pathways, demonstrating broad downstream reactivity. This discovery establishes guanidine bases as a new class of photoactive reductants and highlights aggregation-driven EDA activation as a powerful and underexplored strategy for highly reducing photochemical processes.

## Abstract 2

Commercially available TBD was found to be an effective photoreductant for a variety of aryl halides. The reactivity is proposed to proceed *via* formation of a ternary EDA complex composed of two TBD molecules and one aryl halide.

## Graphical abstract

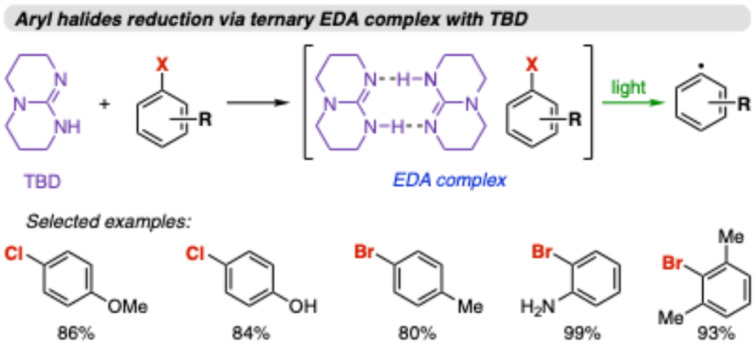

Since the advent of photoredox catalysis, sustained efforts to access more extreme redox potentials have driven the development of increasingly powerful organometallic and organic photocatalysts (PCs) through rational structural design.^1^ Efforts to develop strong photoreductants have focused on achieving the highly reducing potentials required for aryl halide activation. Early work demonstrated that Ir- and Au-based photocatalysts could mediate the reduction of aryl iodides and bromides.^2^ In contrast, the reduction of aryl chlorides remained a long-standing challenge, with most reported examples limited to electron-poor substrates.^1*d*–*f*,3^ Notably, even alternative strategies that circumvent the energetic penalty of aryl halide radical anion formation—such as halogen atom transfer—have only succeeded in reducing unactivated aryl chlorides when employing highly reactive reagents that render tributyltin or triethylsilyl radicals.^4^

Systems capable of reducing a range of aryl chlorides have required increasingly sophisticated approaches. König expanded this reactivity to challenging substrates through the development of photocatalysts capable of two-photon absorption, thereby accessing highly reducing excited states.^5^ In a complementary dual activation strategy, initial chemical or electrochemical reduction of the photocatalyst precedes photoexcitation. This enables the reduced photocatalyst to reach the necessary reduction potentials for these demanding transformations upon photoexcitation.^6^ Finally, anionic photocatalysts have also been reported to mediate these challenging reductions, albeit with reduced functional group compatibility (Fig. 1a).^7^

**Fig. 1:**
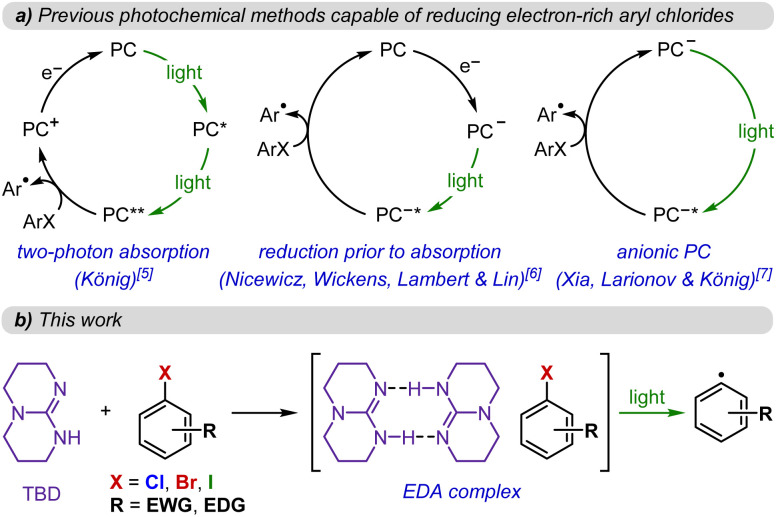
(a) Previous strategies to afford reductions of aryl halides including electron-rich aryl chlorides by photochemical means and (b) this work.

In contrast, we report a milder approach where commercially available guanidinium-type bases can unexpectedly act as strong photoreductants, enabling the reduction of both activated and unactivated aryl iodides, bromides, and chlorides, as well as the Birch-type reduction of extended π-systems.^8^ This unusual reactivity is proposed to arise from the formation of rare ternary electron donor–acceptor (EDA) complexes^9^ containing the aryl halide and a dimer of the guanidine base, as evidenced by absorption, emission, and NMR spectroscopic data. These ternary ground-state aggregates facilitate efficient photoinduced electron transfer, providing a straightforward and accessible platform for achieving highly reducing photochemical transformations without the need for a designer photocatalyst and/or strong external reductants (Fig. 1b). Additionally, the strategy described in this work harnesses close association between the photocatalyst and the substrate, further distinguishing it from previously reported approaches that have been shown to proceed through the generation of solvated electrons.^10^

We demonstrate the generality of the method for generating aryl radical intermediates through the hydrodehalogenation of a broad range of arenes. However, the reactivity of aryl radicals extends beyond simple reduction, enabling a variety of downstream transformations.^2–7^ To illustrate this potential, we highlight representative examples including borylation and intramolecular cyclization, underscoring the broader synthetic utility of the aryl radicals accessible under our mild conditions.

Beyond expanding synthetic capabilities, this study reveals mechanistic nuances relevant to ongoing debates in “photocatalyst-free” reactivity. Similar conditions have previously been reported to enable “photocatalyst-free” organometallic reactions, which, in light of our findings, may involve unrecognized photoinduced formation of aryl radical intermediates.^11^ More broadly, this work suggests that species lacking a clear chromophore may represent an underexplored strategy for achieving photochemical transformations *via* aggregation-induced absorbance. This may be a potentially general yet underappreciated mode of activation in photochemical redox processes.

This project originated from our observation that indoles serve as effective reducing agents for bromoarenes under visible-light irradiation.^12^ While the method proved effective for electron-poor arenes, electron-rich substrates gave poor yields. For example, the reduction of *p*-bromoanisole only afforded an 8% yield of the debrominated arene. To expand this chemistry toward electron-rich arenes, we selected *p*-bromotoluene (1a) as a model substrate and started an optimization campaign. When screening for bases, we discovered guanidine-type bases dramatically improved the reactivity, and control experiments revealed that the indole photocatalyst was no longer required for the reduction when using these bases. Further optimization ultimately led to the conditions shown in Table 1, entry 1.

**Table 1:** Optimized conditions and control experiments

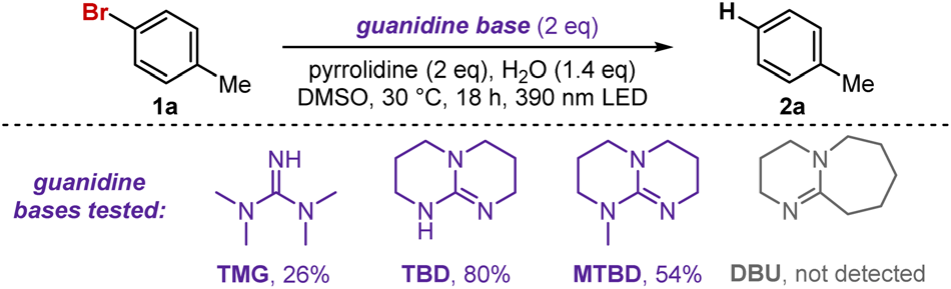			
Entry	Guanidine base	Variation	Yield
1	TBD	None	80%
2	None	No guanidine base	11%
3	TBD	No light	Not detected
4	TBD	427 nm lamp	2%
5	TBD	No pyrrorlidine	38%
6	TBD	NEt_3_ (2 eq) as amine	52%
7	TBD	No water added	68%
8	TBD	1 equiv TBD	68%
9	TBD	Open to air	9%

a Reaction conditions: 1a (0.2 mmol), guanidine base (0.4 mmol), pyrrolidine (0.4 mmol), H_2_O (0.28 mmol), and 0.5 mL of DMSO. The solution was irradiated with a 390 nm LED and stirred at 30 °C for 18 h. All yields were determined by ^1^H NMR using 1,3,5-trimethoxybenzene as an internal standard.

TBD (1,5,7-triazabicyclo[4.4.0]dec-5-ene) displayed superior performance compared to other guanidine-containing bases. Notably, no reactivity was observed when using related scaffolds lacking the guanidine moiety, such as DBU (1,8-diazabicyclo[5.4.0]undec-7-ene) (Table 1). Interestingly, a small amount of product was formed even in the absence of TBD; however, despite extensive efforts, yields never exceeded ∼10% (entry 2). We hypothesize that electron-rich amines may also form EDA complexes with the arene, albeit far less efficiently. No reaction occurred without light, and irradiation at higher wavelengths resulted in significantly diminished yields (entries 3 and 4).

The addition of an amine was not strictly necessary for product formation; however, including an amine significantly improved yields (entry 5). Without an amine, substantial TBD degradation was detected by NMR, whereas in its presence the TBD concentration remained essentially constant throughout the reaction. This observation suggests that the amine serves as the terminal reductant, reducing the TBD radical cation generated from arene reduction (*vide infra*). Among the amines evaluated, pyrrolidine provided the highest yields, although other amines, such as triethylamine, were also effective in promoting the reaction (entry 6). When no water was added, or when the amount of TBD was reduced to one equivalent, a moderate decrease in yield was observed (entries 7 and 8). In contrast, performing the reaction open to air resulted in a dramatic drop in yield, consistent with behavior commonly observed in photochemical transformations involving open-shell species (entry 9).

The substrate scope demonstrated broad functional group tolerance, minimal steric influence on reactivity, and unexpectedly strong performance with traditionally challenging electron-rich substrates (Fig. 2). We began by evaluating a Hammett series of *para*-substituted aryl chlorides, bromides, and iodides to assess electronic effects on the dehalogenation reaction (1a–1f). High yields were obtained for all bromides and iodides, independent of the electronic nature of the substituent. Remarkably, excellent yields were also achieved for aryl chlorides, and even electron-rich examples such as phenol 1e′ and anisole 1f′ were successfully reduced in 84% and 86% yields, respectively, when the reaction time was extended to 65 h. To the best of our knowledge, such efficient reduction of electron-rich aryl chlorides has only been reported using specialized systems requiring the photocatalyst to undergo either two-photon excitation, a reduction event prior to excitation, or be anionic in nature.^5–7^ The fact that this transformation proceeds efficiently using such a simple and economical system is therefore particularly noteworthy. Also significant is the tolerance of unprotected phenols and anilines, which consistently led to high yields ranging between 75% and 99% (1e, 1g, and 1m).

**Fig. 2:**
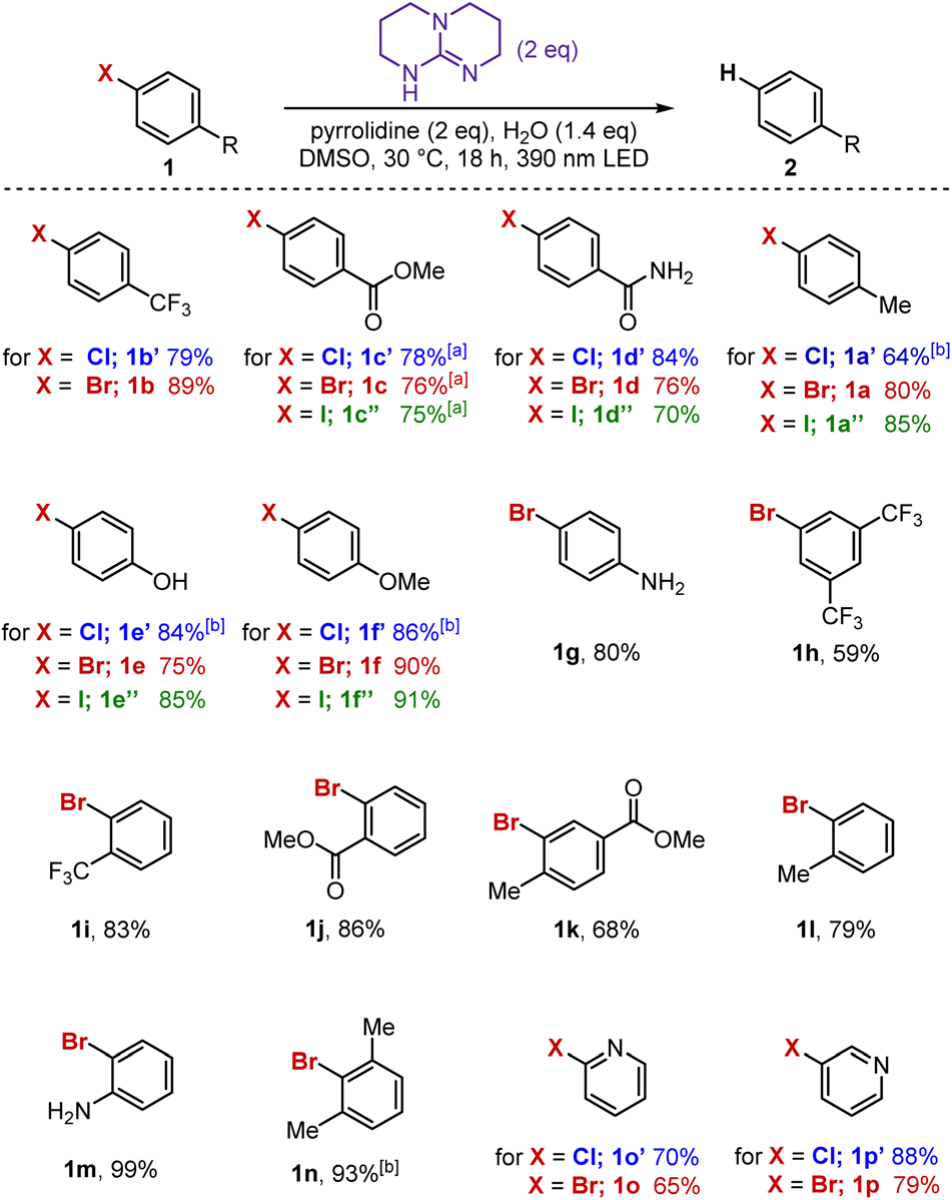
Dehalogenation of chloro- (1′) bromo- (1) and iodoarenes (1″). Reaction conditions: 1 (0.2 mmol), TBD (0.4 mmol), pyrrolidine (0.4 mmol), H_2_O (0.28 mmol), and 0.5 mL of DMSO. The solution was irradiated with a 390 nm LED and stirred at 30 °C for 18 h. All yields were determined by the average of two replicates using ^1^H NMR, with 1,3,5-trimethoxybenzene as an internal standard. ^[a]^No pyrrolidine used. ^[b]^Reaction time: 65 h.

We next examined the effect of different substitution patterns and steric hindrance on the reaction. Once again, high yields were obtained, with the reaction showing little to no sensitivity to *ortho* substitution (1i–1m). Even the electron-rich *ortho*-disubstituted bromide 1n afforded excellent yields when the reaction time was extended to 65 h. Finally, both chloro- and bromo-substituted pyridines (1o and 1p) were successfully reduced, providing moderate to good yields.

Similar to other haloarene reduction protocols, the reactivity observed here can be harnessed beyond simple dehalogenation. To demonstrate the breadth of downstream transformations accessible from the putative intermediate aryl radical, we explored three representative examples (Fig. 3), noting that this is only a subset of the many possibilities known for aryl radical chemistry.^2–7^

**Fig. 3:**
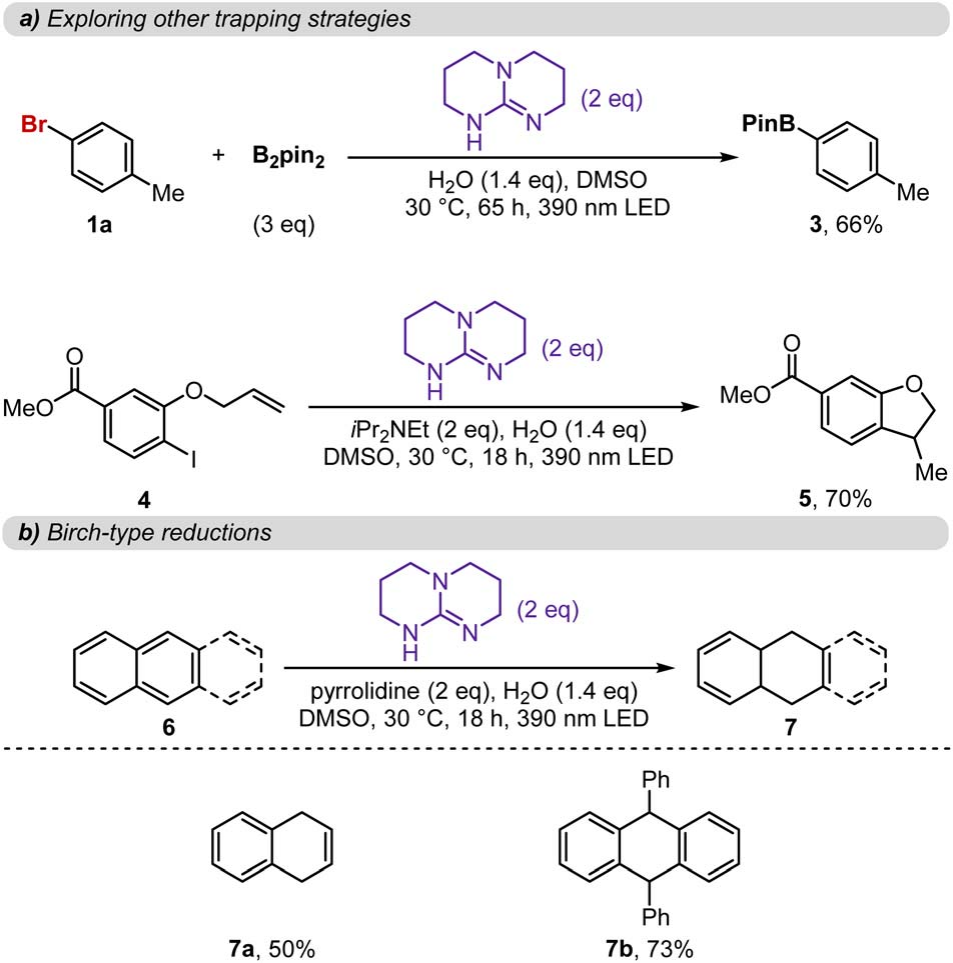
Exploring further reactivity of the putative aryl radical intermediate. (a) Other coupling partners. Top reaction conditions: 1a (0.4 mmol), TBD (0.8 mmol), B_2_Pin_2_ (1.2 mmol), H_2_O (0.28 mmol), and 0.5 mL of DMSO. The solution was irradiated with a 390 nm LED and stirred at 30 °C for 65 h. Bottom reaction conditions: 4 (0.2 mmol), TBD (0.4 mmol), diisopropylethylamine (0.4 mmol), H_2_O (0.28 mmol), and 0.5 mL of DMSO. The solution was irradiated with a 390 nm LED and stirred at 30 °C for 18 h. The reported yields are of the isolated final products 3 and 5, respectively. (b) Birch-type reaction conditions: 6 (0.2 mmol), TBD (0.4 mmol), pyrrolidine (0.4 mmol), H_2_O (0.28 mmol), and 0.5 mL of DMSO. The solution was irradiated with a 390 nm LED and stirred at 30 °C for 18 h. All yields were determined by the average of two replicates using ^1^H NMR, with 1,3,5-trimethoxybenzene as an internal standard.

First, trapping with pinacol borane delivers the corresponding aryl boronic ester 3, a versatile handle for subsequent cross-coupling reactions. Second, substrates bearing pendant olefins undergo intramolecular cyclization, exemplified by a 5-*exo*-trig radical cyclization to furnish 5 in good yield. Finally, under these conditions polyarenes undergo Birch-type reduction,^8^ providing the corresponding dearomatized products in moderate to good yields (7a and 7b).

Given the lack of an obvious chromophore in our key additive, TBD, we conducted a series of spectroscopic experiments to identify the photoactive species responsible for the observed reactivity.^13^ We first evaluated whether the reactivity could arise from a pervasive impurity commonly present in commercial guanidine bases, given that multiple guanidines promoted product formation (Table 1). However, different batches of TBD, including material sourced from multiple vendors as well as TBD recrystallized from toluene, all provided comparable yields in the model reaction. These results suggest that the observed chemistry is intrinsic to TBD rather than due to an impurity.

We continued by measuring the UV-vis absorption spectra of the arenes 1a and 1c, TBD, and mixtures thereof (Fig. 4a). A very weak absorbance was observed for each component individually (purple and red spectra). However, upon mixing the two reaction components, a new absorption band emerged (green spectrum).

**Fig. 4:**
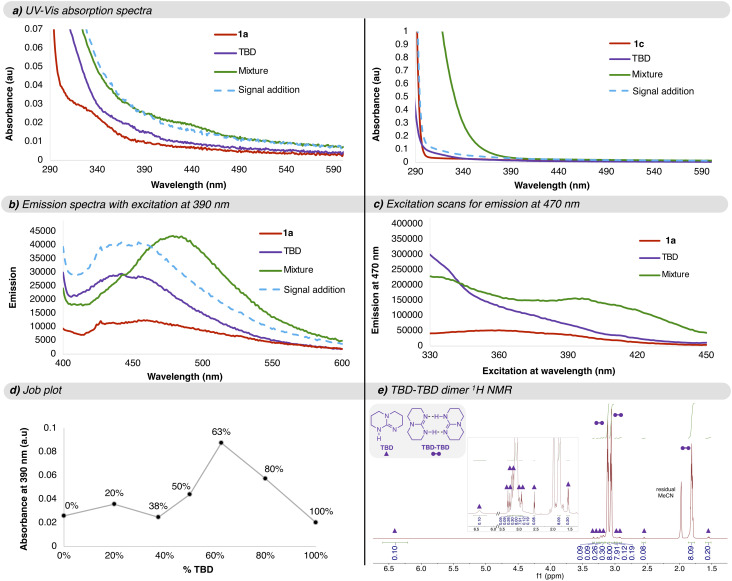
Characterization of the photoactive species. (a) UV-vis absorption spectra of 0.4 M solutions of 1a or 1c (red spectra, top and bottom, respectively), 0.8 M TBD (purple spectrum), and a mixture of 0.8 M TBD and 0.4 M 1a or 1c (green spectra, top and bottom, respectively) in CH_3_CN measured in a 10 mm-path cuvette. The light-blue dashed trace is the simulated sum of the red and purple spectra. (b) Emission spectra irradiating at 390 nm of 0.4 M solution of 1a (red spectrum), 0.8 M TBD (purple spectrum), and a mixture of 0.8 M TBD and 0.4 M 1a (green spectrum) in CH_3_CN measured in a 10 mm-path cuvette. The light-blue dashed trace is the simulated sum of the red and purple spectra. (c) Excitation scan measuring the emission at 470 nm of 0.4 M solution of 1a (red spectrum), 0.8 M TBD (purple spectrum), and a mixture of 0.8 M TBD and 0.4 M 1a (green spectrum) in CH_3_CN measured in a 10 mm-path cuvette. (d) Job plot of 1c and TBD where the absorbance at 390 nm is plotted for solutions containing different ratios of 1c and TBD, maintaining the total concentration ([1c] and [TBD]) constant at 0.4 M in DMSO. (e) ^1^H NMR of a 0.4 M solution of TBD in CD_3_CN showing 10 : 1 dimer : monomer ratio.

This effect is particularly evident for the combination of TBD and the electron-poor arene 1c, where the summed spectra of the individual reagents (dashed light-blue trace) differ markedly from the spectrum of the mixture shown in green (bottom plot). For the mixture of 1a and TBD, there is a slight enhancement of the absorbance between 390 and 450 nm, suggesting that a new species may be formed (top plot). Because the emergence of a new absorption feature is consistent with EDA complex formation that could explain the observed reactivity,^9^ and because the diminished spectral changes with more electron-rich arenes may suggest a potential shift in mechanism for these substrates, we conducted additional studies focusing on the TBD/1a mixture.

Next, the emission spectra irradiating at the reaction wavelength, 390 nm, were measured. The substrate 1a signal was barely above the background, but both the TBD and mixed samples gave clear emission lineshapes (red, purple, and green spectra, respectively, Fig. 4b). Notably, mixing both components resulted in a red-shifted emission band relative to the simulated sum of the 1a and TBD spectra, shifting from ∼450 nm to ∼470 nm (dashed light-blue *vs.* solid purple traces). To further examine the origin of this emission, an excitation scan monitoring the emission at 470 nm was conducted. This experiment probes the absorption spectra of the ground-state species that gives rise to the emission band of interest at 470 nm. It should be noted that this is different from transient absorption spectroscopy, which probes the absorption spectrum of an excited state species. As shown in Fig. 4c, again a clear divergence between the traces of the individual components compared to the mixture (red and purple *vs.* green) was observed, consistent with the formation of a new emissive species. Additionally, the new emissive feature in the excitation scan (390 to 440 nm) coincides with the region where the absorption enhancement is seen in Fig. 4a. This provides direct evidence for the formation of an EDA complex between TBD and electron-rich arene 1a, suggesting that EDA complex formation is not limited to mixtures of TBD and electron-poor arenes like 1c.

Finally, we measured the time-resolved photoluminescence from both the TBD and TBD/1a solutions in CH_3_CN. The TBD solution emission lifetime of ∼2 ns was redshifted and shortened to less than 1 ns when 1a was added (Fig. S6), suggesting that the presence of 1a opens up a new nonradiative relaxation pathway for the complex. Since Förster transfer from TBD to 1a is not energetically possible, the fluorescence quenching likely reflects electron transfer from the TBD to 1a,^14^ which would provide the initial step in the photoredox reaction.

Subsequently, we sought to determine the stoichiometry of the reaction components responsible for forming the photoactive EDA complex. To this end, we performed a Job plot analysis in which the total concentration of TBD and aryl bromide 1c was held constant while varying their molar ratio, and the absorbance at the reaction wavelength (390 nm) was monitored. A maximum in absorbance was observed at approximately a 2 : 1 TBD : aryl bromide ratio, indicating the formation of an unusual termolecular EDA complex composed of two equivalents of TBD and one equivalent of aryl bromide (Fig. 4d). An analogous trend was observed when monitoring the absorption at 430 nm (see SI Fig. S4), the peak absorbance of the complex according to the data displayed in Fig. 4a.

To rationalize this stoichiometry, we examined the aggregation behavior of TBD by ^1^H NMR in CD_3_CN at the same concentration used in the reduction reaction (Fig. 4e). The spectrum indicates that TBD exists predominantly as a symmetric dimer, giving rise to three distinct signals, contrasting with the non-symmetrical monomer, which displays nine signals highlighted in the spectra by the purple triangles.^15^ Integration of these signals suggests an approximate 10 : 1 dimer-to-monomer ratio (*i.e.*, one monomer for every 21 TBD molecules in solution).

Taken together, these experiments strongly support the formation of a ground-state ternary EDA complex displaying new absorption and emission features. The data is consistent with the association of dimeric TBD with the aryl halide to form the photoactive complex, wherein photoinduced electron transfer from [TBD]_2_ to the arene accounts for the observed reactivity.

To further assess the feasibility of photochemical reduction *via* this EDA complex and to gain insight into its mechanistic features, we computationally investigated the association between dimeric TBD and bromotoluene (1a). Electronic-structure calculations (see details in the SI) reveal several low-energy conformers of the [TBD]_2_⋯bromotoluene assembly that are thermodynamically accessible in solution, with multiple structures exhibiting exergonic formation free energies (Fig. S8, Table S6). The most stable conformer is predicted to form with Δ*G* = −5.0 kcal mol^−1^ and features the bromoaryl positioned above one of the TBD units, while preserving the H-bonded TBD–TBD dimer motif (Fig. 5a). Interestingly, the lowest-lying excited states of these complexes display pronounced CT (charge transfer) character, with the hole primarily localized on the TBD fragment and the aryl moiety acting as the electron acceptor (Fig. S9). These transitions are associated with small oscillator strengths (Table S7), consistent with the modest enhancement of the long-wavelength absorption tail observed experimentally (Fig. 4a). Furthermore, excited-state geometry optimization on the singlet manifold locate a CT minimum (Fig. 5b) featuring weak radiative intensity and significantly red-shifted emission energy relative to isolated TBD (monomer and dimer, Table S8). This computed deactivation energy can thus be assigned to the distinctive low-energy emission band observed for the mixture in Fig. 4b (green trace).

**Fig. 5:**
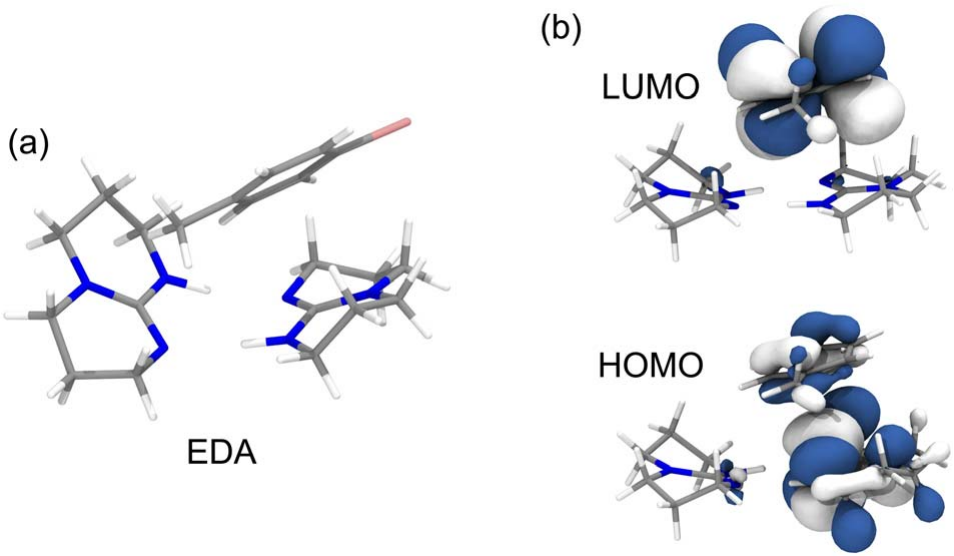
Computational results. (a) Structure of the EDA complex optimized at the CAM-B33LYP-D3/6-311++G(d,p) computational level in acetronitrile solution. (b) Frontier molecular orbitals for the lowest CT state of EDA obtained at its equilibrium geometry.

It is surprising that such a complex could give rise to low energy states that enable absorption around 400 nm, however, hydrogen-bonding (as in the TBD dimer) is known to support electronic delocalization.^16^ This type of noncovalent interaction has been shown to give rise to new lower energy states that exhibit “clusteroluminescence” in a wide variety of systems, including hydrogen-bonded amines.^17^ However, this phenomenon has not been previously utilized to drive catalysis. While a detailed study is beyond the scope of this communication, we believe that a similar effect may be responsible for the red-shifted absorption observed for TBD, with the dimer displaying spectral features extending into the visible region that would not be expected for the monomer alone. The possibility that such cluster-based electronic states could participate in photocatalysis has not previously been recognized. Ongoing studies in our laboratories aim to further elucidate the photophysical behavior of concentrated TBD solutions.

With these results in hand, we propose the mechanism depicted in Fig. 6. Beginning from the TBD–TBD dimer, which is the dominant species in solution under the reaction conditions (Fig. 4e),^15^ association with the aryl halide (1) forms a ternary EDA complex (Fig. 4 and 5). Upon photoexcitation, characteristic of EDA complexes, single-electron transfer from the electron-rich TBD dimer to the aryl halide occurs.^9^ The resulting aryl halide radical anion undergoes mesolysis to generate the aryl radical, this was confirmed by a spin trap experiment that supports the formation of aryl radical intermediates (Fig. S7). Then, this open shell species engages in productive pathways leading to products 2, 3, 5, and 7. In parallel, the oxidized TBD dimer is proposed to be reduced back to its neutral form *via* single-electron transfer from the amine additive, completing the reductive cycle.

**Fig. 6:**
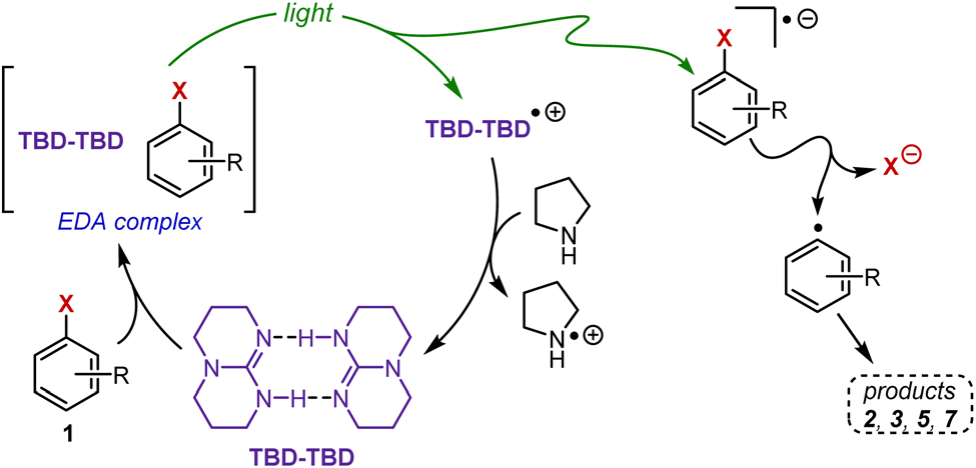
Proposed mechanism.

## Conclusions

In summary, we have shown that commercially available guanidine-type bases, particularly TBD, can act as unexpectedly potent photoreductants, enabling the reduction of aryl iodides, bromides, and even electron-rich aryl chlorides under 390 nm light irradiation. This simple system achieves transformations that previously required sophisticated two-photon excitation strategies, reduction-excitation methods, or anionic photocatalysts.^5–7^ Mechanistic studies suggest that key to this reactivity is the formation of an unusual ternary EDA complex between a TBD dimer and the aryl halide. Upon light excitation, efficient electron transfer from the TBD dimer^15^ to the aryl halide generates an aryl radical, which engages in a variety of productive transformations, while the neutral TBD dimer is regenerated *via* single-electron transfer from an amine additive to complete a closed photoredox cycle.

More broadly, the demonstration that a noncovalent assembly can act as a potent visible light electron-transfer agent may open new avenues for the design of photocatalyst systems. The identification of this species relied on combining emission spectra with excitation scans as a complementary photophysical approach for identifying EDA complexes when UV-vis data alone is inconclusive—an aspect that remains underutilized and occasionally debated within the synthetic community.^9*a*,*f*^

Finally, the synthetic utility of this system is broad, as it renders aryl radicals which can be integrated with established downstream protocols. Remarkably, this methodology shows minimal sensitivity to steric hindrance or electronic effects, delivering high yields across *para*-, *meta*-, and *ortho*-substituted arenes, and tolerating traditionally challenging electron-rich substrates. Collectively, these findings demonstrate the power of leveraging ground-state aggregate formation in “photocatalyst-free” systems and highlight the potential of simple organic bases as highly reducing photochemical reagents.

## Author contributions

R. J. H. and A. B. conceived of the presented idea and designed the experimental methods. R. J. H. and A. M. V. performed the optimization and scope exploration experiments. S. C., K. H., and C. J. B. conceived and carried out the time resolve spectroscopic experiments. C. T. and D. C. conceived and carried out the computational studies. A. B. wrote the manuscript with contributions of all the authors.

## Conflicts of interest

There are no conflicts to declare.

## Supplementary Material

SC-OLF-D6SC00251J-s001

## Data Availability

Experimental procedures and characterization data are provided as supplementary information (SI). The authors have seen and approved this submission, and this manuscript has been exclusively submitted to *Chemical Science*. Supplementary information is available. See DOI: https://doi.org/10.1039/d6sc00251j.
