# Supplementary material for: Photochemical reduction of aryl chlorides, bromides, and iodides via ternary EDA complexes with guanidine bases
Source: Chem Sci. 2026 Feb 26;17(17):8486–92. doi: 10.1039/d6sc00251j (PMC12974598; doi:10.1039/d6sc00251j)
Supplement: SC-017-D6SC00251J-s001 [file SC-017-D6SC00251J-s001.pdf]

Supplementary Information (SI) for Chemical Science.
This journal is © The Royal Society of Chemistry 2026

Photochemical Reduction of Aryl Chlorides, Bromides, and Iodides via Ternary EDA Complexes with Guanidine Bases

Robert J. Hannan,[a] Alexandria M. Vondran,[a] Sunghwan Cho,[a] Kerry Hanson,[a] Claire Tonnelé,[b] David Casanova,[b] Christopher J. Bardeen,[a] and Ana Bahamonde*[a]

[a] Department of Chemistry, University of California, Riverside
501 W Big Springs Rd., Riverside, CA, 92521, USA

[b] Ikerbasque Researcher, Donostia International Physics Center (DIPC)
Paseo Manuel Lardizabal 4, 20018 Donostia, Euskadi, Spain

E-mail: ana.bahamonde@ucr.edu

Table of Contents:

1. General Considerations S2
2. General Procedures S3
3. Optimization of Reaction Conditions S4
4. UV-Vis Absorption Spectra of the Mixtures of the Reaction Components S6
5. Excitation Scan of TBD and 4-Bromotoluene Mixture S9
6. Time-resolved Fluorescence Experiments S10
7. Spin Trap Experiment S11
8. Scope Entries S12
9. Computational Studies S24
10. References S27
11. NMR Spectra S30

1. General Considerations

All reagents were purchased form commercial suppliers and used without further purification unless otherwise stated. TBD was dried periodically prior to use, as reduced yield was observed when left open to air over time. The TBD was subjected to high vacuum for two hours once a week for consistency. We suggest as best practice to storage it in a desiccator as well as the periodic cycles of high vacuum. ^{1}H NMR spectra were obtained in $CDCl_3$, benzene-d_6, or acetonitrile-d_3 at 400 MHz or 600 MHz. Chemical shifts are reported in ppm and referenced to the $CHCl_3$ singlet at 7.26 ppm, the benzene singlet at 7.16 ppm, or the acetonitrile singlet at 1.94 ppm. ^{13}C NMR spectra were obtained in $CDCl_3$, benzene-d_6, at 101 MHz or 151 MHz and referenced to the center peak of the $CDCl_3$ triplet at 77.16 ppm. The abbreviations s, d, t, quint, sext, hept, dd, ddd, dt, m, brs, brd, brt, brm, and ABq stand for the resonance multiplicities singlet, doublet, triplet, quintet, sextet, heptet, doublet of doublets, doublet of doublet of doublets, doublet of triplets, multiplet, broad singlet, broad doublet, broad triplet, broad multiplet, and AB quartet respectively. Thin-layer chromatography was performed with EMD silica gel 60 F254 plates eluting with solvents indicated, visualized by a 254 nm UV lamp, and stained with vanillin stain as necessary. UV-Vis spectra were acquired using on an Agilent Technologies Cary 60 UV-Vis at room temperature. Emission spectra were collected on a Horiba PTI QM-400 Fluorescence spectrophotometer at room temperature. NMR, UV-Vis, and Emission spectra were obtained using the Analytical Chemistry Instrumentation Facility at the University of California, Riverside.

2. General Procedures

General Procedure A, Photo-dehalogenation

In an oven dried 10 mL Schlenk tube under constant nitrogen flux, an oven dried glass coated stir bar is added, followed by 0.5 mL of dry DMSO, 0.5 µL of water, dry TBD (55.7 mg, 0.4 mmol), aryl halide (0.2 mmol), and pyrrolidine (33 µL, 0.4mmol). Flasks are sealed using silicon grease and parafilm before being placed in baths to maintain constant 30 °C temperature and stirred at 350 rpm. The Schlenk flask is placed touching wall of glass water bath directly in line with the center of the 390 nm Kessil LED lamp. Reaction is run for 18 hours generally, select substrates run for longer as noted. After irradiation, 1,3,5 trimethoxybenzene was added to the crude reaction mixture and use as an internal standard. An aliquot of the mixture was taken for ^{1}H NMR analysis.

General Procedure B, Birch-type Reactions

In an oven dried 10 mL Schlenk tube under constant nitrogen flux, an oven dried glass stir bar is added, followed by 0.5 mL of dry DMSO, 0.5 µL of water, dry TBD (55.7 mg, 0.4 mmol), Aryl halide (0.2 mmol), and pyrrolidine (33 µL, 0.4 mmol). Flasks are sealed using silicon grease and parafilm before being placed in baths to maintain constant 30 °C temperature and and stirred at 350 rpm. The Schlenk flask is placed touching wall of glass water bath directly in line with the center of the 390 nm Kessil LED lamp. Reaction is run for 18 hours. After irradiation, 1,3,5 trimethoxybenzene was added to the crude reaction mixture and use as an internal standard. An aliquot of the mixture was taken for ^{1}H NMR analysis.

3. Optimization of Reaction Conditions

Model Reaction:

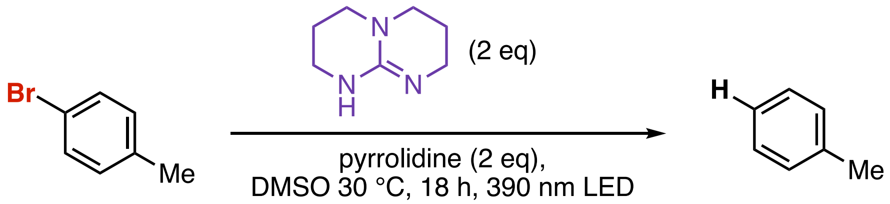

Reactions set up according to the general procedure A. Reaction conditions: **1a** (0.2 mmol), guanidine base (0.4 mmol), pyrrolidine (0.4 mmol), H_2O (0.28 mmol), and 0.5 mL of DMSO. The solution was irradiated with a 390 nm LED and stirred at 30 °C for 18 h. All yields were determined by ^{1}H NMR using 1,3,5-trimethoxybenzene as an internal standard.

Table S1. Water Loading

Entry	Water Loading	Yield
1	0 μL	68%
2	5 μL	80%
3	10 μL	73%

Table S2. Amine Screening

Entry	Sacrificial Amine	Yield
1	NEt_3	52%
2	Pyrrolidine	80%
3	No Sacrificial Amine	38%

Table S3. Solvent Screening

Entry	Solvent	Yield
1	MeCN	10%
2	DMF	6%
3	DMSO	80%

Table S4. Base Screening

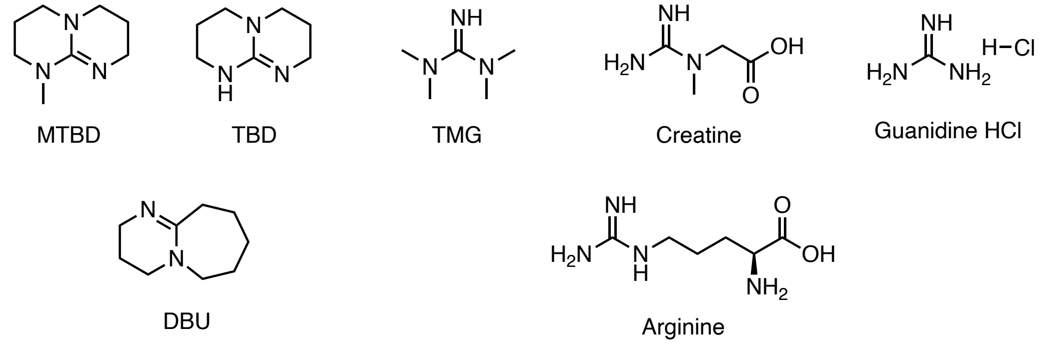

Entry	Base	Yield
1	TMG	26%
2	DBU	0%
3	MTBD	54%
4	TBD	80%
5	1 equiv TBD	68%
6	Creatine	7%
7	Arginine	3%
8	Guanidine HCl	11%

Table S5. Controls

Entry	Variation	Yield
1	No Light	0%
2	No TBD	11%
3	No Stirring	22%
4	Open to Air	9%
5	427nm lamp	2%

4. UV-Vis Absorption Spectra of the Mixtures of the Reaction Components

4.1. UV-Vis absorption spectra of TBD mixed with 4-bromotoluene (1a)

Solutions of 0.8 M TBD (1,5,7-triazabicyclo[4.4.0]dec-5-ene), 0.4 M of 4-bromotoluene (**1a**), and of both 0.8 M TBD and 0.4 M 4-bromotoluene were made in 20 mL scintillation vial using MeCN. The solutions were allowed to fully dissolve before transferring to 10 mm pathlength quartz cuvette. UV-Vis absorption spectra were collected on an Agilent Technologies Cary 60 UV-Vis at room temperature.

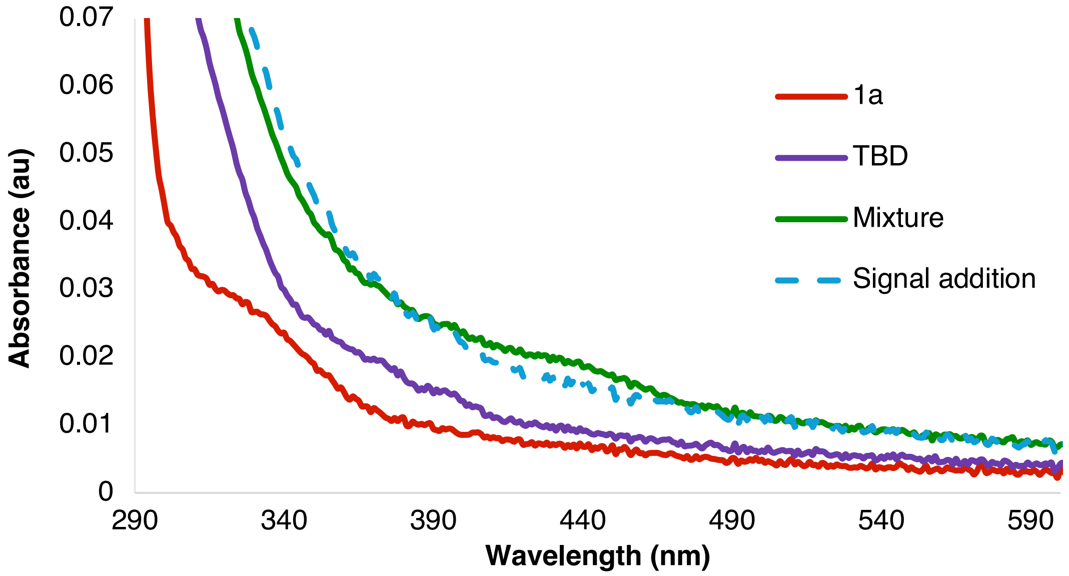

Figure S1. UV-Vis absorption spectra of 0.4 M solutions of **1a** (red spectrum), 0.8 M TBD (purple spectrum), and a mixture of 0.8 M TBD and 0.4 M **1a** (green spectrum) in CH_3CN measured in a 10 mm-path cuvette. The light-blue dashed trace is the simulated sum of the red and purple spectra.

4.2. UV-Vis absorption spectra of TBD mixed with methyl-4-bromobenzoate (1c)

Solutions of 0.8 M TBD (1,5,7-triazabicyclo[4.4.0]dec-5-ene), 0.4 M of methyl-4-bromobenzoate (**1c**), and of both 0.8 M TBD and 0.4 M 4-bromotoluene were made in 20 mL scintillation vial using MeCN. The solutions were allowed to fully dissolve before transferring to 10 mm pathlength quartz cuvette. UV-Vis absorption spectra were collected on an Agilent Technologies Cary 60 UV-Vis at room temperature.

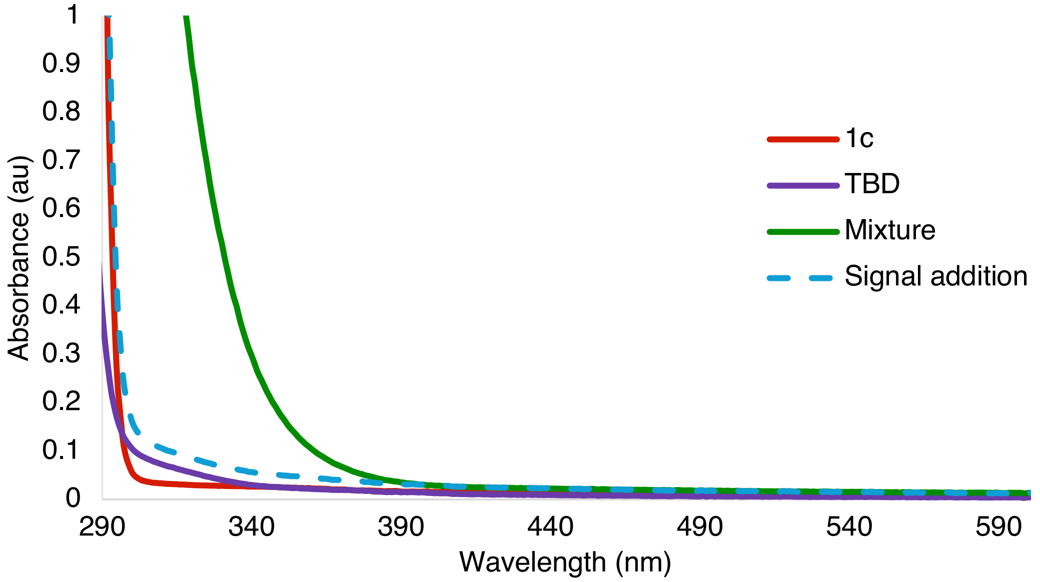

Figure S2. UV-Vis absorption spectra of 0.4 M solutions of **1c** (red spectrum), 0.8 M TBD (purple spectrum), and a mixture of 0.8 M TBD and 0.4 M **1c** (green spectrum) in CH_3CN measured in a 10 mm-path cuvette. The light-blue dashed trace is the simulated sum of the red and purple spectra.

4.3. Job Plot of TBD and methyl 4-bromobenzoate (1c)

A series of samples were made varying the molar ratio of TBD and methyl 4-bromobenzoate while holding the total concentration constant at 0.4 M. The solutions were made in 20 mL scintillation vials using DMSO as solvent. The solutions were allowed to fully dissolve before transferring to 10 mm pathlength quartz cuvette. UV-Vis absorption spectra were collected on an Agilent Technologies Cary 60 UV-Vis at room temperature. The mixture absorbance was measured for each sample and plotted in Figure S3. Subsequently, the absorbance of the mixtures at 390 and 430 nm was plotted against the TBD molar ratio to create the Job plots Figure S4 and S5, respectively.

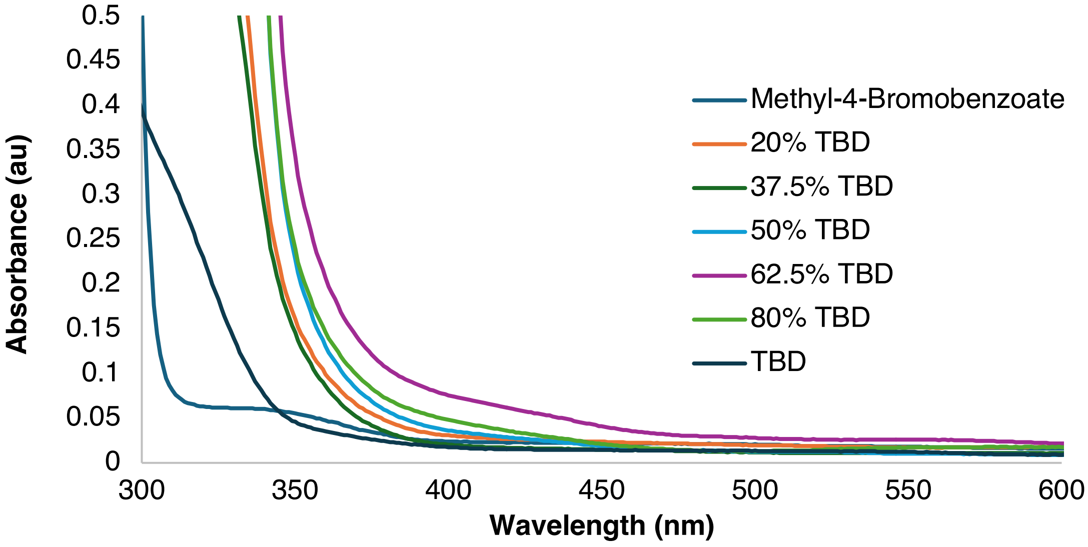

Figure S3. UV-Vis absorption spectra of **1c** and TBD solutions with combined total concentration but varied ratios between them of 0.4 M in DMSO measured in a 10 mm-path cuvette.

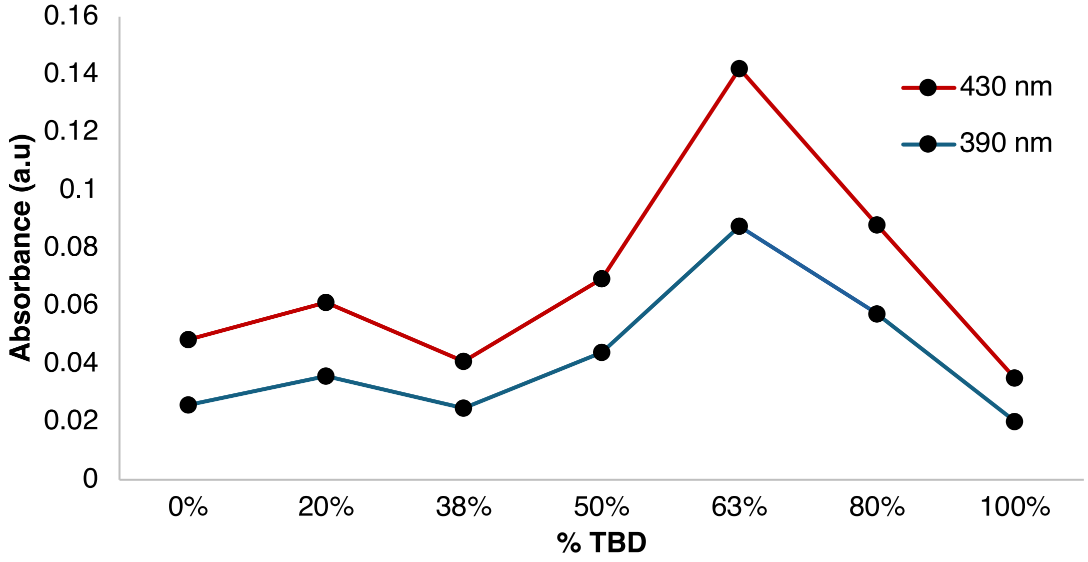

Figure S4. Job plot of **1c** and TBD where the absorbance at 390 nm (blue) and 430 nm (red) are plotted for solutions containing different ratios of **1c** and TBD maintaining the total concentration ([**1c**] and [TBD]) constant at 0.4 M in DMSO.

5. Excitation Scan of TBD and 4-Bromotoluene Mixture

A 0.8 M solution of TBD and another and 0.4 M solution of 4-bromotoluene (**1a**) were prepared in 20 mL scintillation vials using 3 mL of MeCN. The solutions were allowed to fully dissolve before and sparged with N_2 for 10 minutes prior to transferring to 10 mm pathlength quartz cuvette. Emission spectra were collected on a Horiba PTI QM-400 Fluorescence spectrophotometer at room temperature. Emission measured at 470 nm.

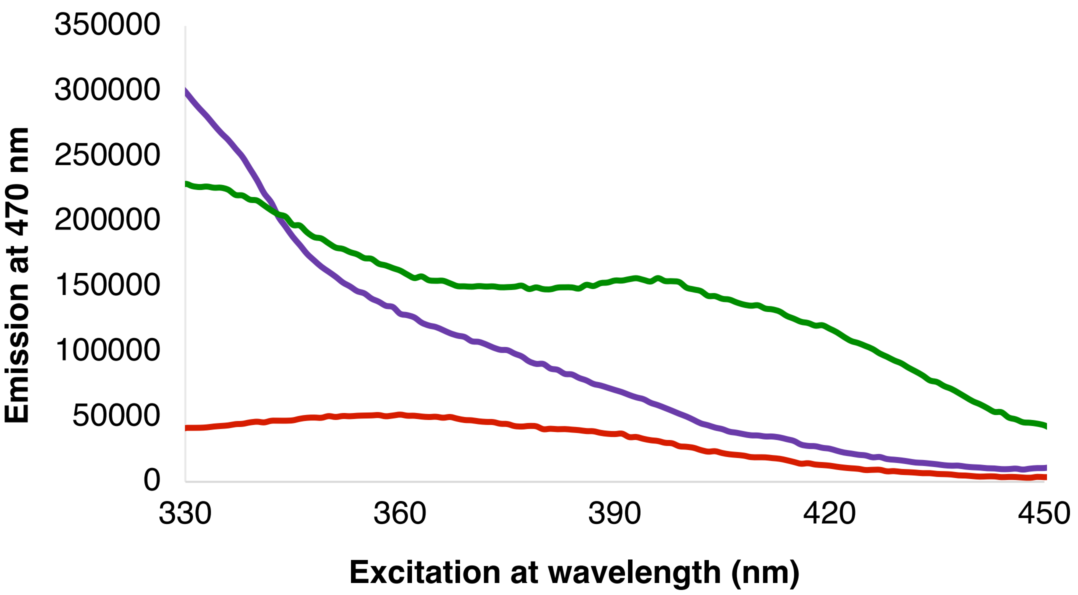

Figure S5. Excitation scan measuring the emission at 470 nm of 0.4 M solution of **1a** (red spectrum), 0.8 M TBD (purple spectrum), and a mixture of 0.8 M TBD and 0.4 M **1a** (green spectrum) in CH_3CN.

6. Time-resolved Fluorescence Experiments

A 0.8 M solution of TBD and another and 0.4 M solution of 4-bromotoluene (**1a**) were prepared in 20 mL scintillation vials using 3 mL of MeCN. The solutions were allowed to fully dissolve before and sparged with N_2 for 10 minutes prior to transferring to 10 mm pathlength quartz cuvette. The fluorescence lifetimes were taken using front face detection with a Hamamatsu C4334 streakscope picosecond streak camera. The 400 nm excitation was generated by frequency doubling the 800 nm pulse from a 40 kHz Spectra-Physics Spitfire Ti:sapphire regenerative amplifier. Excitation at 400 nm.

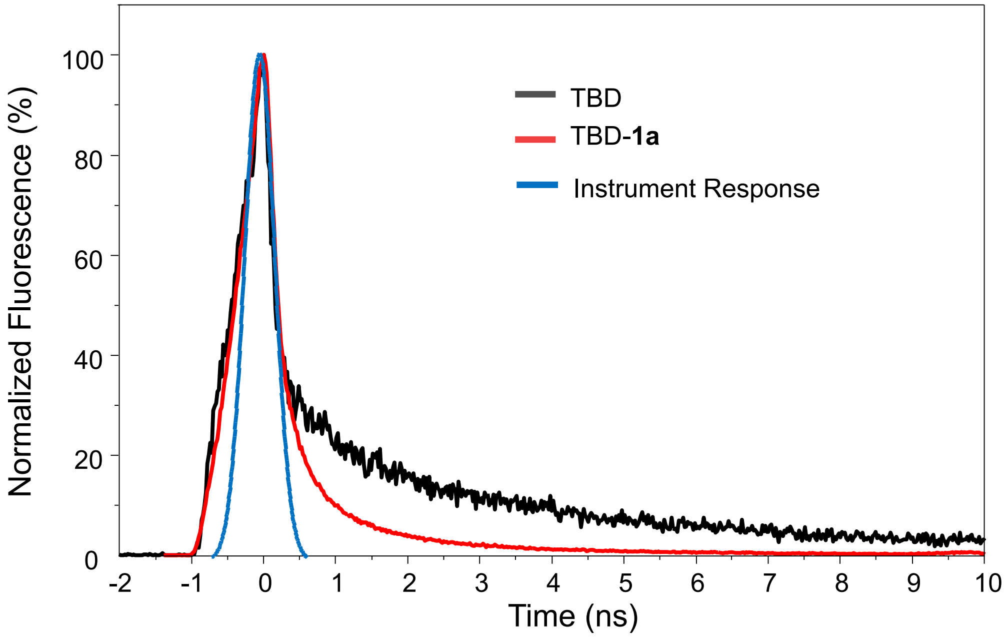

Figure S6. Time-resolved fluorescence decays of a 0.8 M solution of TBD (black) and a mixture of 0.8 M TBD and 0.4 M **1a** (TBD-**1a**, red) in CH_3CN. The instrument response function (blue) is also shown. The TBD-**1a** data is less noisy because it has a higher initial signal but decays more rapidly than TBD by itself. This fluorescence quenching is consistent with excitation followed by electron transfer to **1a**.

7. Spin Trap Experiment

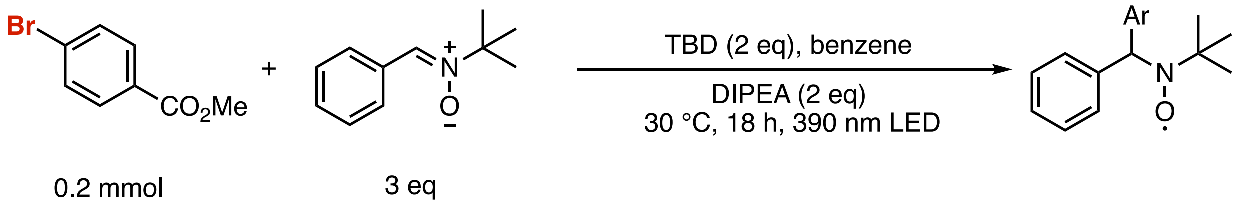

N-tertbutyl-α-phenylnitrone (0.6 mmol, 106.3 mg) was added to 0.5 mL of benzene. This solution was transferred to an oven dried 10 mL Schlenk under constant nitrogen. To this a glass coated stir bar was added, followed by TBD (0.4 mmol, 55.7 mg), methyl-4-bromobenzoate (0.2 mmol, 43 mg), and DIPEA (0.4 mmol, 70 µL). This was then placed in a 30 ºC water bath and irradiated with 390 nm light for 18 h. This solution was transferred to an EPR tube under nitrogen, then sealed with a Teflon stopper and parafilm. The EPR spectrum was acquired at room temperature (Bruker Magnetech ESR 5000, microwave frequency: 1000 kHz, power = 2.0 mW, modulation amplitude = 0.1 mT). The observed signals match previous literature. [1]

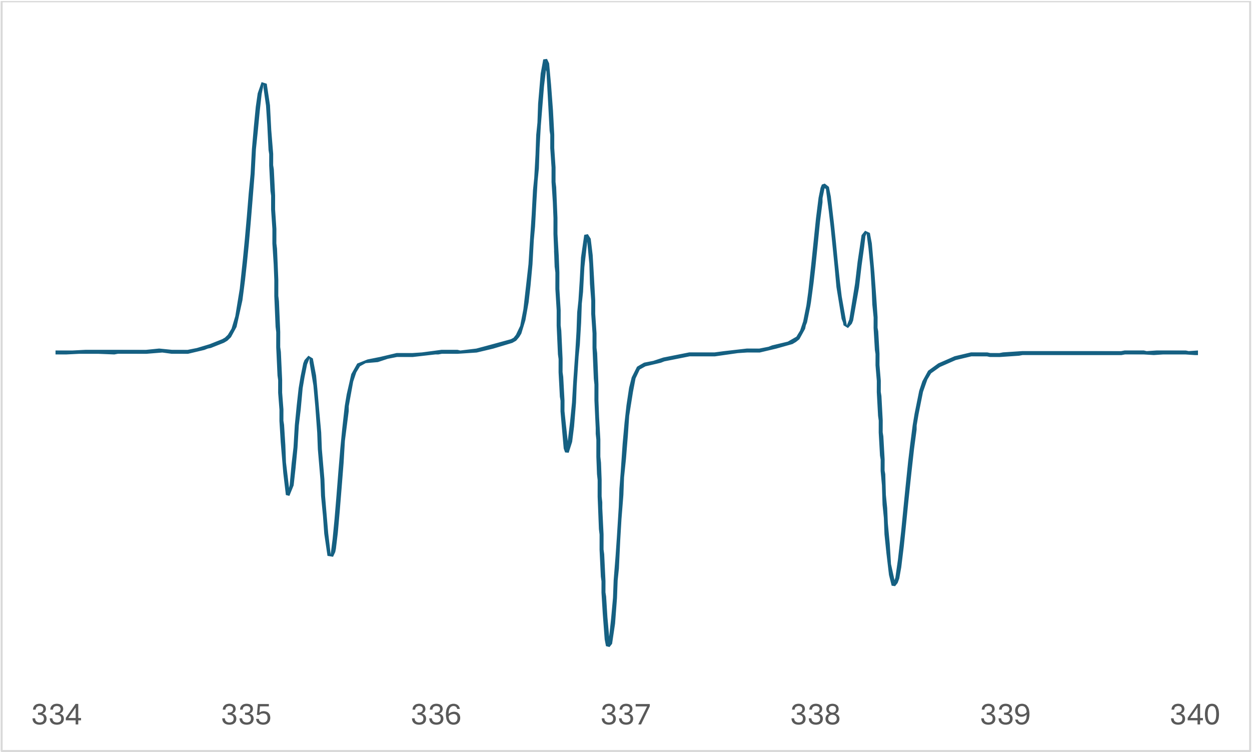

Figure S7. X Band EPR Spectrum of reaction crude.

8. Scope Entries

8.1. Dehalogenation Reactions

4-Bromotoluene

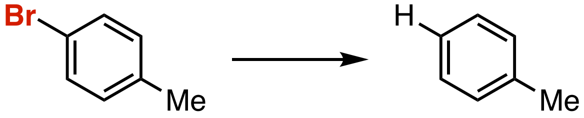

1a

Following general procedure A, 4-bromotoluene (25 µL, 0.2 mmol) was dehalogenated over 18 h to yield a yellow/green clear solution. NMR yield was calculated using 1,3,5-trimethoxybenzene as internal standard, reported as the average of 2 replicates (80%). ^{1}H NMR (600 MHz, $CDCl_3$) δ 7.25 (t, J = 7.6 Hz, 2H), 7.20 – 7.14 (m, 3H), 2.35 (s, 3H). The characterization data matches a previous report.[2]

4-Chlorotoluene

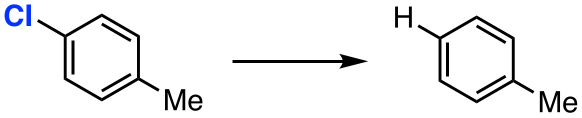

1a'

Following general procedure A, 4-chlorotoluene (24 µL, 0.2 mmol) was dehalogenated over 65 h to yield an orange solution. NMR yield was calculated using 1,3,5-trimethoxybenzene as internal standard (64%). ^{1}H NMR (600 MHz, $CDCl_3$) δ 7.25 (t, J = 7.5 Hz, 2H), 7.18-7.14 (m, 3H), 2.35 (s, 3H). The characterization data matches a previous report.[2]

4-Iodotoluene

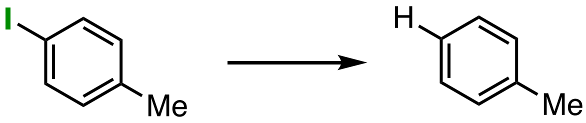

1a"

Following general procedure A, 4-iodotoluene (43.6 mg, 0.2 mmol) was dehalogenated over 18 h to yield a yellow/green clear solution. NMR yield was calculated using 1,3,5-trimethoxybenzene as internal standard, reported as the average of 2 replicates (85%). ^{1}H NMR (600 MHz, $CDCl_3$) δ 7.25 (t, J = 7.3 Hz, 2H), 7.16 (t, J = 7.3 Hz, 3H), 2.35 (s, 3H). The characterization data matches a previous report.[2]

4-Bromotrifluorotoluene

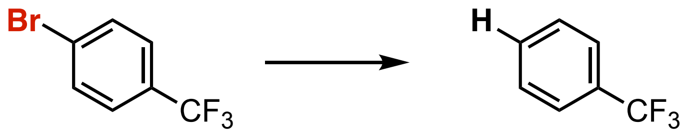

1b

Following general procedure A, 4-bromotrifluorotoluene (28 μL, 0.2 mmol) was dehalogenated over 18 h to yield a yellow/green clear solution. NMR yield was calculated using 1,3,5-trimethoxybenzene as internal standard, reported as the average of 2 replicates (89%). ^{1}H NMR (600 MHz, $CDCl_3$) δ 7.63 (d, J = 7.8 Hz, 2H), 7.56 (t, J = 7.8 Hz, 1H), 7.49 (t, J = 7.8 Hz, 2H). The characterization data matches a previous report.[3]

4-Chlorotrifluorotoluene

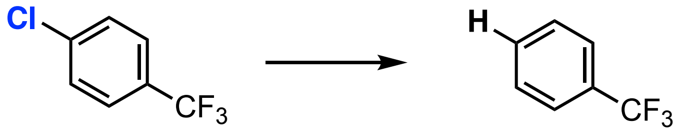

1b'

Following general procedure A, 4-chlorotrifluorotoluene (27 μL, 0.2 mmol) was dehalogenated over 18 h to yield a yellow/green clear solution. NMR yield was calculated using 1,3,5-trimethoxybenzene as internal standard, reported as the average of 2 replicates (79%). ^{1}H NMR (600 MHz, $CDCl_3$) δ 7.63 (d, J = 7.4 Hz, 2H), 7.56 (d, J = 7.8 Hz, 1H), 7.50 (t, J = 7.4 Hz, 2H). The characterization data matches a previous report.[3]

Methyl 4-bromobenzoate

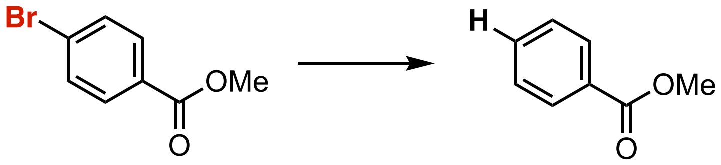

1c

Following general procedure A, methyl 4-bromobenzoate (43 mg, 0.2 mmol) was dehalogenated over 18 h to yield a yellow/green clear solution. NMR yield was calculated using 1,3,5-trimethoxybenzene as internal standard, reported as the average of 2 replicates (76%). ^{1}H NMR (600 MHz, $CDCl_3$) δ 8.04 (s, 2H), 7.56 (s, 1H), 7.45 (d, J = 10.7 Hz, 2H), 3.92 (s, 3H). The characterization data matches a previous report.[4]

Methyl 4-chlorobenzoate

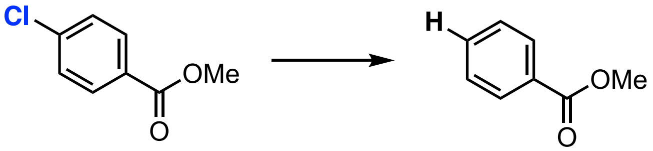

1c'

Following general procedure A but excluding pyrrolidine, methyl 4-chlorobenzoate (34.6 mg, 0.2 mmol) was irradiated with a 390 nm LED and stirred at 30 °C for 18 h to yield a yellow/green clear solution. NMR yield was calculated using 1,3,5-trimethoxybenzene as internal standard, reported as the average of 2 replicates (78%). ^{1}H NMR (600 MHz, $CDCl_3$) δ 8.05 (d, J = 7.7 Hz, 2H), 7.57 (t, J = 7.7 Hz, 1H), 7.45 (t, J = 7.7 Hz, 2H), 3.93 (s, 3H). The characterization data matches a previous report.[4]

Methyl 4-iodobenzoate

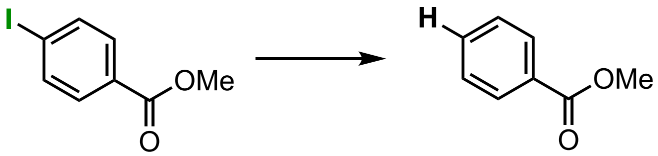

1c"

Following general procedure A but excluding pyrrolidine, methyl 4-iodobenzoate (52.4 mg, 0.2 mmol) was irradiated with a 390 nm LED and stirred at 30 °C for 18 h to yield a yellow/green clear solution. NMR yield was calculated using 1,3,5-trimethoxybenzene as internal standard, reported as the average of 2 replicates (75%). ^{1}H NMR (500 MHz, $CDCl_3$) δ 8.06 – 8.00 (m, 2H), 7.56 (t, J = 7.6 Hz, 1H), 7.44 (t, J = 7.6 Hz, 2H), 3.91 (s, 3H). The characterization data matches a previous report.[4]

4-Bromobenzamide

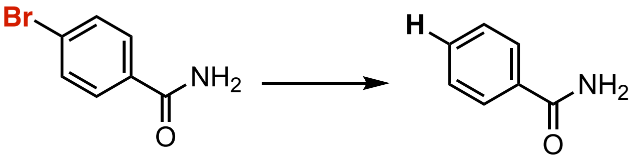

1d

Following general procedure A, 4-bromobenzamide (40 mg, 0.2 mmol) was irradiated with a 390 nm LED and stirred at 30 °C for 18 h to yield a yellow/green clear solution. NMR yield was calculated using 1,3,5-trimethoxybenzene as internal standard, reported as the average of 2 replicates (76%). ^{1}H NMR (500 MHz, $CDCl_3$) δ 7.87 – 7.81 (m, 2H), 7.54 – 7.48 (m, 1H), 7.43 (t, J = 7.6 Hz, 2H). The characterization data matches a previous report.[5]

4-Chlorobenzamide

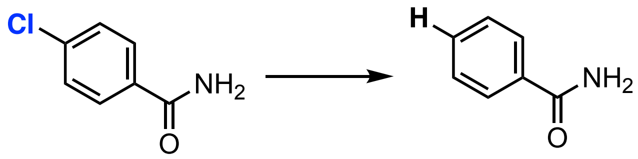

1d'

Following general procedure A, 4-chlorobenzamide (31.1 mg, 0.2 mmol) was irradiated with a 390 nm LED and stirred at 30 °C for 18 h to yield a yellow/green clear solution. NMR yield was calculated using 1,3,5-trimethoxybenzene as internal standard, reported as the average of 2 replicates (84%). ^{1}H NMR (600 MHz, $CDCl_3$) δ 7.85 (d, J = 7.2 Hz, 2H), 7.52 (d, J = 7.2 Hz, 1H), 7.45 (t, J = 7.2 Hz, 2H). The characterization data matches a previous report.[5]

4-Iodobenzamide

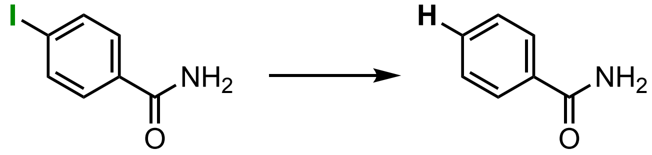

1d"

Following general procedure A, 4-iodobenzamide (49.4 mg, 0.2 mmol) was irradiated with a 390 nm LED and stirred at 30 °C for 18 h to yield a yellow/green clear solution. NMR yield was calculated using 1,3,5-trimethoxybenzene as internal standard, reported as the average of 2 replicates (70%). ^{1}H NMR (600 MHz, $CDCl_3$) δ 7.84 (d, J = 7.1 Hz, 2H), 7.52 (t, J = 7.1 Hz, 1H), 7.45 (d, J = 7.1 Hz, 2H). The characterization data matches a previous report.[5]

4-Bromophenol

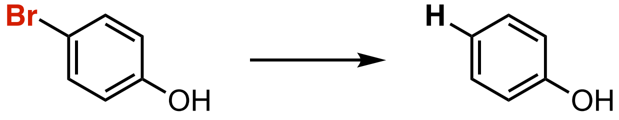

1e

Following general procedure A, 4-bromophenol (34.6 mg, 0.2 mmol) was irradiated with a 390 nm LED and stirred at 30 °C for 18 h to yield a yellow/green clear solution. NMR yield was calculated using 1,3,5-trimethoxybenzene as internal standard, reported as the average of 2 replicates (75%). ^{1}H NMR (600 MHz, $CDCl_3$) δ 7.11 (bs, 2H), 6.71 (d, J = 7.5 Hz, 2H), 6.61 (bs, 1H). The characterization data matches a previous report with a slight shift due to hydrogen bonding with the phenol.[6]

4-Chlorophenol

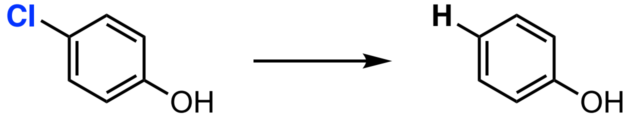

1e'

Following general procedure A, 4-chlorophenol (25.7 mg, 0.2 mmol) was irradiated with a 390 nm LED and stirred at 30 °C for 65 h to yield an orange solution. NMR yield was calculated using 1,3,5-trimethoxybenzene as internal standard, reported as the average of 2 replicates (84%). ^{1}H NMR (600 MHz, $CDCl_3$) δ 7.18 (t, J = 7.6 Hz, 2H), 6.87 – 6.83 (m, 2H), 6.80 (t, J = 7.6 Hz, 1H). The characterization data matches a previous report with a slight shift due to hydrogen bonding with the phenol.[6]

4-Iodophenol

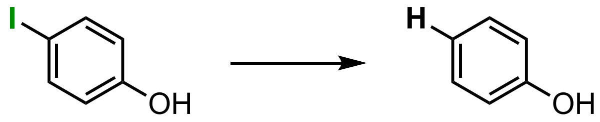

1e"

Following general procedure A, 4-iodobenzamide (49.4 mg, 0.2 mmol) was irradiated with a 390 nm LED and stirred at 30 °C for 18 h to yield a yellow/green clear solution. NMR yield was calculated using 1,3,5-trimethoxybenzene as internal standard, reported as the average of 2 replicates (85%). ^{1}H NMR (600 MHz, $CDCl_3$) δ 7.12 (d, J = 8.8 Hz, 2H), 6.73 (s, 2H), 6.63 (d, J = 6.8 Hz, 1H). The characterization data matches a previous report with a slight shift due to hydrogen bonding with the phenol.[6]

4-Bromoanisole

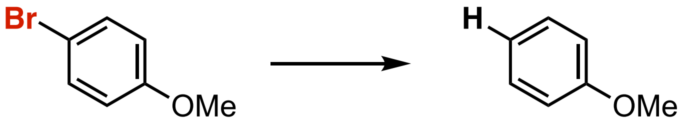

1f

Following general procedure A, 4-bromoroanisole (25 μL, 0.2 mmol) was irradiated with a 390 nm LED and stirred at 30 °C for 18 h to yield an orange solution. NMR yield was calculated using 1,3,5-trimethoxybenzene as internal standard, reported as the average of 2 replicates (90%). ^{1}H NMR (600 MHz, $CDCl_3$) δ 7.35 – 7.25 (m, 2H), 6.95 (t, J = 7.7 Hz, 1H), 6.91 (d, J = 7.7 Hz, 2H), 3.82 (s, 3H). The characterization data matches a previous report.[7]

4-Chloroanisole

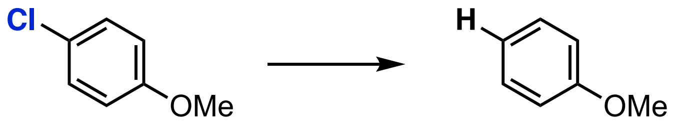

1f'

Following general procedure A, 4-chloroanisole (24 µL, 0.2 mmol) was irradiated with a 390 nm LED and stirred at 30 °C for 65 h to yield an orange solution. NMR yield was calculated using 1,3,5-trimethoxybenzene as internal standard, reported as the average of 2 replicates (86%). ^{1}H NMR (600 MHz, $CDCl_3$) δ 7.31 – 2.28 (m, 2H), 6.95 (t, *J* = 7.4 Hz, 1H), 6.91 (d, *J* = 7.4 Hz, 2H), 3.81 (s, 3H). The characterization data matches a previous report.[7]

4-Iodoanisole

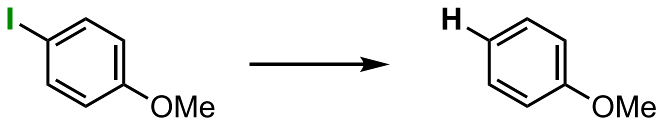

1f''

Following general procedure A, 4-iodoanisole (46.8 mg, 0.2 mmol) was irradiated with a 390 nm LED and stirred at 30 °C for 18 h to yield a yellow/green clear solution. NMR yield was calculated using 1,3,5-trimethoxybenzene as internal standard, reported as the average of 2 replicates (91%). ^{1}H NMR (600 MHz, $CDCl_3$) δ 7.30 – 7.27 (m, 2H), 6.94 (t, *J* = 6.0 Hz, 1H), 6.91 (d, *J* = 6.0 Hz, 2H), 3.81 (s, 3H). The characterization data matches a previous report.[7]

4-Bromoaniline

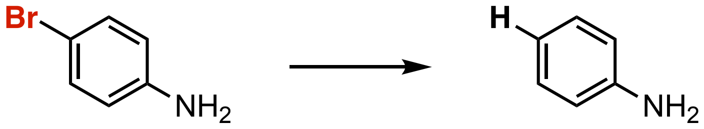

1g

Following general procedure A, 4-bromoaniline (34.4 mg, 0.2 mmol) was irradiated with a 390 nm LED and stirred at 30 °C for 18 h to yield a yellow clear solution. NMR yield was calculated using 1,3,5-trimethoxybenzene as internal standard, reported as the average of 2 replicates (80%). ^{1}H NMR (600 MHz, $CDCl_3$) δ 7.18 – 7.13 (m, 2H), 6.75 (d, *J* = 6.6 Hz, 1H), 6.69 (d, *J* = 7.4 Hz, 2H). The characterization data matches a previous report.[8]

1,3-Bis(trifluoromethyl)-5-bromobenzene

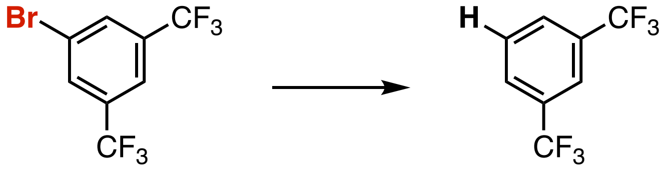

1h

Following general procedure A, 1,3-bis(trifluoromethyl)-5-bromobenzene (35 µL, 0.2 mmol) was irradiated with a 390 nm LED and stirred at 30 °C for 18 h to yield a dark orange solution. NMR yield was calculated using 1,3,5-trimethoxybenzene as internal standard, reported as the average of 2 replicates (59%). ^{1}H NMR (600 MHz, $CDCl_3$) δ 7.91 (s, 1H), 7.85 (d, *J* = 7.9 Hz, 2H), 7.67 (t, *J* = 7.9 Hz, 1H). The characterization data matches a previous report.[9]

1-Bromo-2-(trifluoromethyl)benzene

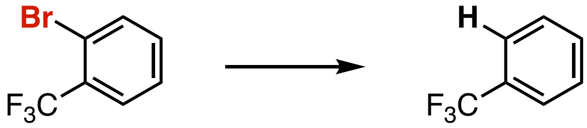

1i

Following general procedure A, 1-bromo-2-(trifluoromethyl)benzene (27 µL, 0.2 mmol) was irradiated with a 390 nm LED and stirred at 30 °C for 18 h to yield an orange solution. NMR yield was calculated using 1,3,5-trimethoxybenzene as internal standard, reported as the average of 2 replicates (83%). ^{1}H NMR (600 MHz, $CDCl_3$) δ 7.63 (d, *J* = 7.9 Hz, 2H), 7.56 (t, *J* = 7.9 Hz, 1H), 7.49 (t, *J* = 7.9 Hz, 2H). The characterization data matches a previous report.[3]

Methyl 2-bromobenzoate

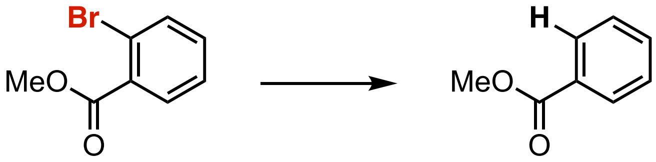

1j

Following general procedure A, methyl 2-bromobenzoate (28.1 µL, 0.2 mmol) was irradiated with a 390 nm LED and stirred at 30 °C for 18 h to yield an orange solution. NMR yield was calculated using 1,3,5-trimethoxybenzene as internal standard, reported as the average of 2 replicates (86%). ^{1}H NMR (600 MHz, $CDCl_3$) δ 8.05 (s, 2H), 7.57 (s, 1H), 7.46 (d, *J* = 9.6 Hz, 2H), 3.93 (s, 3H). The characterization data matches a previous report.[4]

Methyl 3-bromo-4-methylbenzoate

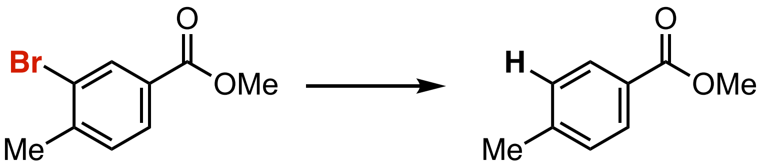

1k

Following general procedure A, methyl 3-bromo-4-methylbenzoate (32 µL, 0.2 mmol) was irradiated with a 390 nm LED and stirred at 30 °C for 18 h to yield a red/orange solution. NMR yield was calculated using 1,3,5-trimethoxybenzene as internal standard, reported as the average of 2 replicates (68%). ^{1}H NMR (600 MHz, $CDCl_3$) δ 7.92 (d, J = 7.6 Hz, 2H), 7.24 (d, J = 7.6 Hz, 2H), 3.90 (s, 3H), 2.41 (s, 3H). The characterization data matches a previous report.[10]

2-Bromotoluene

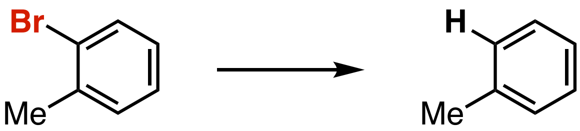

1l

Following general procedure A, 2-bromotoluene (43 mg, 0.2 mmol) was irradiated with a 390 nm LED and stirred at 30 °C for 18 h to yield a yellow/green clear solution. NMR yield was calculated using 1,3,5-trimethoxybenzene as internal standard, reported as the average of 2 replicates (79%). ^{1}H NMR (600 MHz, $CDCl_3$) δ 7.25 (t, J = 7.7 Hz, 2H), 7.19 – 7.14 (m, 3H), 2.35 (s, 3H). The characterization data matches a previous report.[2]

2-Bromoaniline

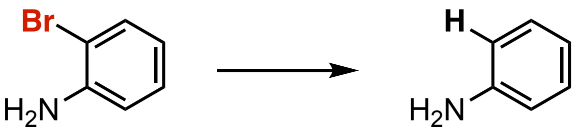

1m

Following general procedure A, 2-bromotoluene (43 mg, 0.2 mmol) was irradiated with a 390 nm LED and stirred at 30 °C for 18 h to yield a yellow/green clear solution. NMR yield was calculated using 1,3,5-trimethoxybenzene as internal standard, reported as the average of 2 replicates (99%). ^{1}H NMR (600 MHz, $CDCl_3$) δ 7.15 (t, J = 7.5 Hz, 2H), 6.75 (t, J = 7.5 Hz, 1H), 6.69 (d, J = 7.5 Hz, 2H). The characterization data matches a previous report.[8]

2-Bromo-*m*-xylene

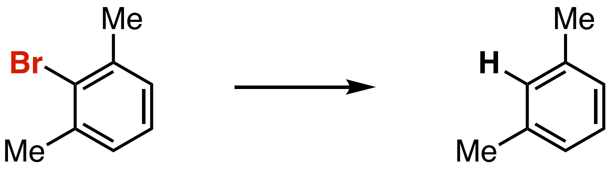

1n

Following general procedure A, 2-bromo-*m*-xylene (27 µL, 0.2 mmol) was irradiated with a 390 nm LED and stirred at 30 °C for 65 h to yield a yellow clear solution. NMR yield was calculated using 1,3,5-trimethoxybenzene as internal standard, reported as the average of 2 replicates (93%). ^{1}H NMR (600 MHz, $CDCl_3$) δ 7.15 (t, *J* = 7.6 Hz, 1H), 7.00 (s, 1H), 6.98 (d, *J* = 7.6 Hz, 2H), 2.32 (s, 6H). The characterization data matches a previous report.[11]

2-Bromopyridine

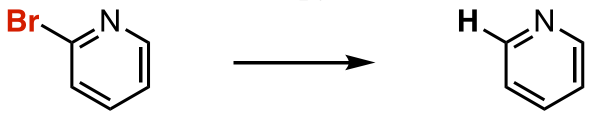

1o

Following general procedure A, 2-bromopyridine (19 µL, 0.2 mmol) was irradiated with a 390 nm LED and stirred at 30 °C for 18 h to yield a yellow clear solution. NMR yield was calculated using 1,3,5-trimethoxybenzene as internal standard, reported as the average of 2 replicates (65%). ^{1}H NMR (600 MHz, $CDCl_3$) δ 8.61 (s, 2H), 7.68 (t, *J* = 7.8 Hz, 1H), 7.31 – 7.29 (m, 2H). The characterization data matches a previous report.[2]

2-Chloropyridine

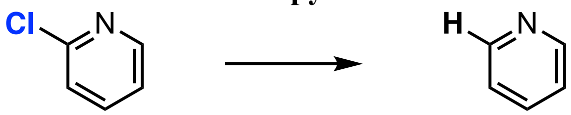

1o'

Following general procedure A, 2-chloropyridine (19 µL, 0.2 mmol) was irradiated with a 390 nm LED and stirred at 30 °C for 18 h to yield a yellow clear solution. NMR yield was calculated using 1,3,5-trimethoxybenzene as internal standard, reported as the average of 2 replicates (70%) ^{1}H NMR (600 MHz, $CDCl_3$) δ 8.60 (s, 2H), 7.67 (d, *J* = 7.5 Hz, 1H), 7.31 – 7.29 (m, 2H). The characterization data matches a previous report.[2]

3-Bromopyridine

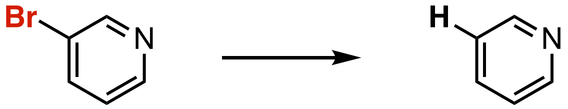

1p

Following general procedure A, 3-bromopyridine (19 µL, 0.2 mmol) was irradiated with a 390 nm LED and stirred at 30 °C for 18 h to yield a yellow clear solution. NMR yield was calculated using 1,3,5-trimethoxybenzene as internal standard, reported as the average of 2 replicates (79%). ^{1}H NMR (600 MHz, $CDCl_3$) δ 8.60 (s, 2H), 7.72 – 7.65 (m, 1H), 7.29 – 7.27 (m, 2H). The characterization data matches a previous report.[2]

3-Chloropyridine

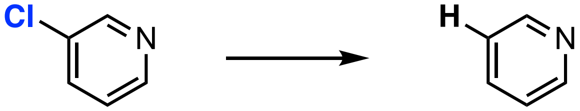

1p'

Following general procedure A, 3-chloropyridine (20 µL, 0.2 mmol) was irradiated with a 390 nm LED and stirred at 30 °C for 18 h to yield a yellow clear solution. NMR yield was calculated using 1,3,5-trimethoxybenzene as internal standard, reported as the average of 2 replicates (88%). ^{1}H NMR (600 MHz, $CDCl_3$) δ 8.62 – 8.59 (m, 2H), 7.68 (t, J = 7.3 Hz, 1H), 7.29 – 7.27 (m, 2H). The characterization data matches a previous report.[2]

8.2. Dehalo-borylation Reaction

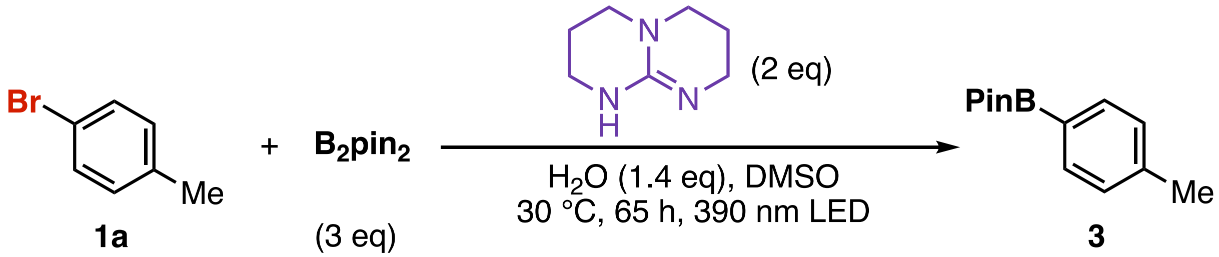

In an oven dried 10 mL Schlenk tube under constant nitrogen flux, an oven dried glass stir bar was added, followed by 1.0 mL of dry DMSO, 10 µL of water, dry TBD (111.4 mg, 0.8 mmol), aryl halide **1a** (0.4 mmol), and B_2Pin_2 (304.8 mg, 1.2 mmol). Flask was sealed using silicon grease and parafilm before being placed in baths to maintain constant 30 °C temperature and stirred at 350

rpm. The Schlenk flask was placed touching wall of glass water bath directly in line with the center of the 390 nm Kessil LED lamp. The mixture was irradiated for 65 hours. The reaction mixture was transferred to a separatory funnel and extracted using 10 mL water, 2 mL of brine, and 3 X 10 mL of ethyl acetate. The organic layer was washed with brine then dried over Na_2SO_4. The solution was concentrated under reduced pressure. This material purified utilizing flash chromatography (98:2 Hexanes:EtOAc) to yield a clear oil (57.5 mg, 66% yield). ^{1}H NMR (600 MHz, $CDCl_3$) δ 7.74 (d, J = 7.5 Hz, 2H), 7.22 (d, J = 7.5 Hz, 2H), 2.40 (s, 3H), 1.37 (s, 12H). ^{13}C NMR (151 MHz, $CDCl_3$) δ 141.4, 134.8, 128.6, 115.0, 83.6, 24.9, 21.8. The characterization data matches a previous report.[12]

8.3. Cyclization Reaction

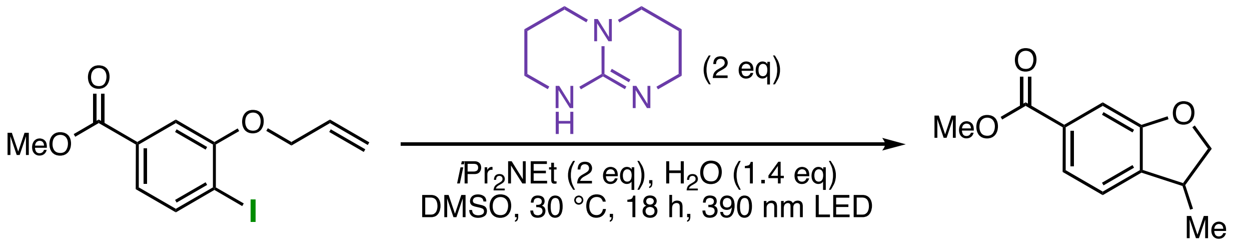

4 **5**

In an oven dried 10 mL Schlenk tube under constant nitrogen flux, an oven dried glass stir bar was added, followed by 0.5 mL of dry DMSO, 0.5 µL of water, dry TBD (55.7 mg, 0.4 mmol), DIPEA (70 µL, 0.4 mmol), (aryl halide **4** (0.2 mmol). The flask was sealed using silicon grease and parafilm before being placed in a bath to maintain constant 30 °C temperature and stirred at 350 rpm. The Schlenk flask was placed touching wall of glass water bath directly in line with the center of the 390 nm Kessil LED lamp. The mixture was irradiated for 18 h. The reaction mixture was transferred to a separatory funnel and extracted using 10 mL of an aqueous saturated solution of K_2CO_3 and 3 X 10 mL of ethyl acetate. The organic layer was washed with brine then dried over Na_2SO_4. The solution was concentrated under reduced pressure. This material purified utilizing flash chromatography (gradient from 100:0 to 80:20 Hexanes:EtOAc) to yield a clear oil (26.8 mg, 70% yield). ^{1}H NMR (600 MHz, $CDCl_3$) δ 7.63 (dd, J = 7.6, 1.5 Hz, 1H), 7.44 (d, J = 1.5 Hz, 1H), 7.25 – 7.20 (m, 1H), 4.76 (t, J = 8.9 Hz, 1H), 4.19 – 4.12 (m, 1H), 3.92 (s, 3H), 3.60 (h, J = 7.2 Hz, 1H), 1.38 (d, J = 7.2 Hz, 3H). ^{13}C NMR (151 MHz, $CDCl_3$) δ 167.0, 159.9, 137.9, 130.4, 123.5, 122.6, 110.3, 78.8, 52.1, 36.5, 19.1. HRMS (ESI-TOF) m/z $[M+H]^+$ calcd. for $C_{11}H_{13}O_3$, 194.0935; found, 193.0855.

8.4. Birch-type Reactions

Napthalene

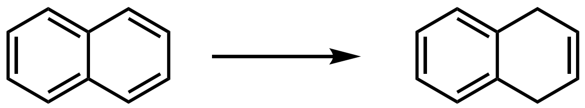

7a

Following general procedure B, napthalene (25.6 mg, 0.2 mmol) was reduced over 18 h to yield a yellow/green solution. NMR yield was calculated using 1,3,5-trimethoxybenzene as internal standard, reported as the average of 2 replicates (50%). ^{1}H NMR (600 MHz, $CDCl_3$) δ 7.14 – 7.12 (m, 4H), 5.92 (s, 2H), 3.36 (s, 4H). The characterization data matches a previous report.[13]

9,10-Diphenylanthracene

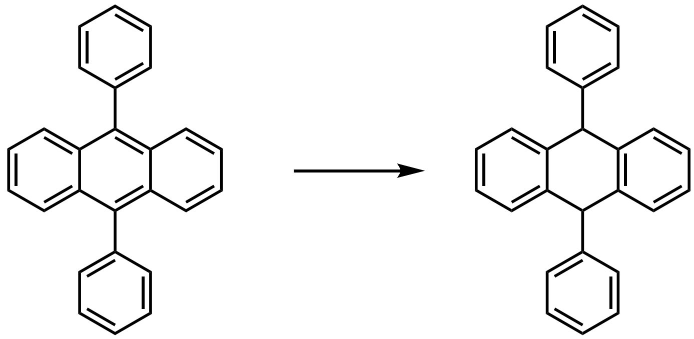

7b

Following general procedure B, 9,10-diphenylanthracene (66.1 mg, 0.2 mmol) was reduced over 18 h to yield an orange solution. NMR yield was calculated using 1,3,5-trimethoxybenzene as internal standard, reported as the average of 2 replicates (78%). ^{1}H NMR (600 MHz, $CDCl_3$) δ 7.36 – 7.28 (m, 4H), 7.27 – 7.22 (m, 2H), 7.20 – 7.10 (m, 12H), 5.24 (s, 2H). The characterization data matches a previous report.[14]

9. Computational Studies

Ground state geometries were optimized within the framework of the density functional theory (DFT) using the CAM-B3LYP exchange-correlation functional (XCF) and the 6-311++G(d,p) basis set, and including the Grimme's D3 correction.[15] Gibbs free energy of formation of the EDA complexes were evaluated from MP2/6-311++G(d,p) single point calculations corrected by the thermal correction to Gibbs Free Energy derived from DFT. On the basis of the obtained equilibrium structures obtained, vertical excitation energies and oscillator strengths were computed at time-dependent (TD) DFT with the CAM-B3LYP XCF and 6-311+G(d,p) basis set. Excited state geometry optimizations were performed with the same XCF and the 6-311+G(d,p) basis set with D3 correction. Solvation effects were taken into account by means of polarizable continuum models (PCM), with the integral equation formalism (IEF-PCM)[16,17] implementation for geometry optimizations and the conductor-like model (C-PCM)[18,19] for calculations of the vertical transition energies. All calculations were performed using Gaussian 16[20] and Q-Chem[21] program packages.

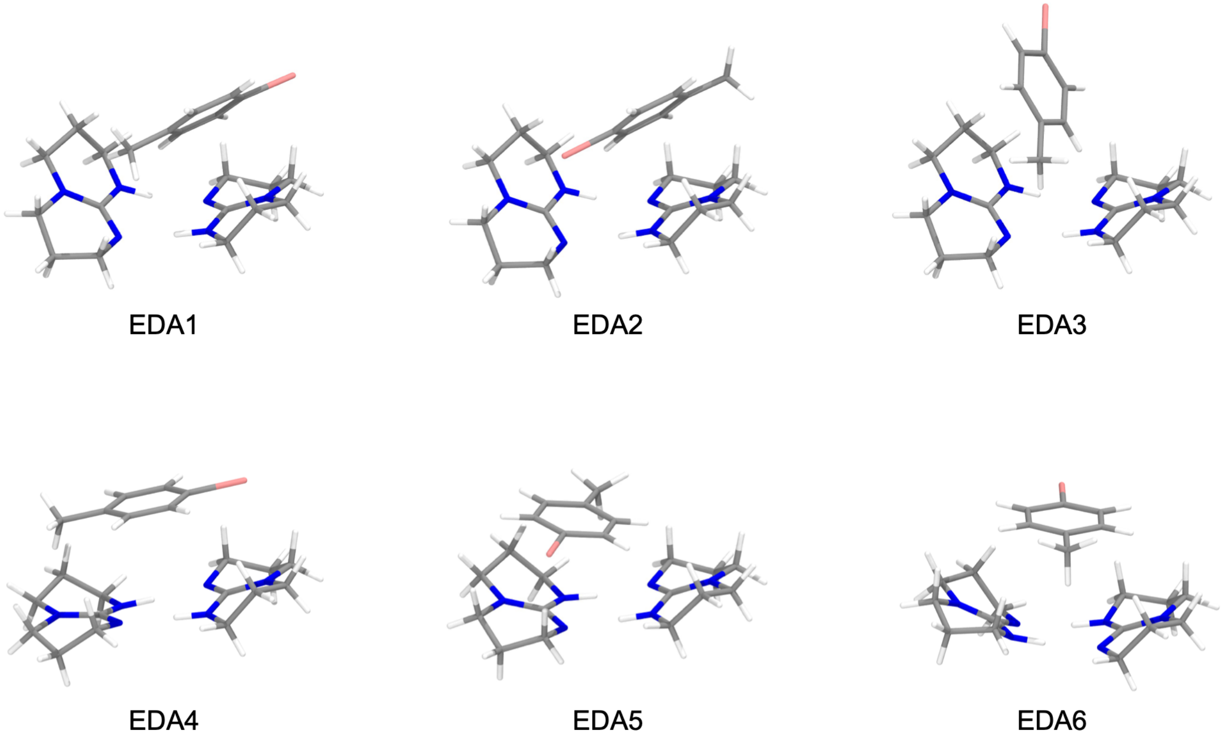

Figure S8. Ground state geometry of the EDA complexes optimized at the CAM-B3LYP-D3/6-311++G(d,p) level in acetonitrile.

Table S6. Gibbs free energy of formation (ΔG, in kcal/mol) of the EDA complexes.

EDA complex	ΔG
EDA1	-4.965
EDA2	-3.945
EDA3	-3.891
EDA4	-2.494
EDA5	-1.716
EDA6	-0.416

Table S7. Vertical transition energies (in eV) and oscillator strengths (in parentheses) of the EDA complexes, calculated at the CAM-B3LYP/6-311+G(d,p) level in acetonitrile at their respective ground state geometry.

EDA complex	S_1	S_2	S_3	S_4
EDA1	5.269 (0.011)	5.352 (0.007)	5.390 (0.005)	5.509 (0.010)
EDA2	5.267 (0.009)	5.340 (0.014)	5.403 (0.010)	5.427 (0.007)
EDA3	5.248 (0.010)	5.377 (0.014)	5.466 (0.016)	5.514 (0.000)
EDA4	5.231 (0.009)	5.300 (0.002)	5.368 (0.005)	5.411 (0.003)
EDA5	5.238 (0.008)	5.277 (0.006)	5.357 (0.004)	5.385 (0.002)
EDA6	5.263 (0.007)	5.350 (0.000)	5.398 (0.001)	5.518 (0.004)

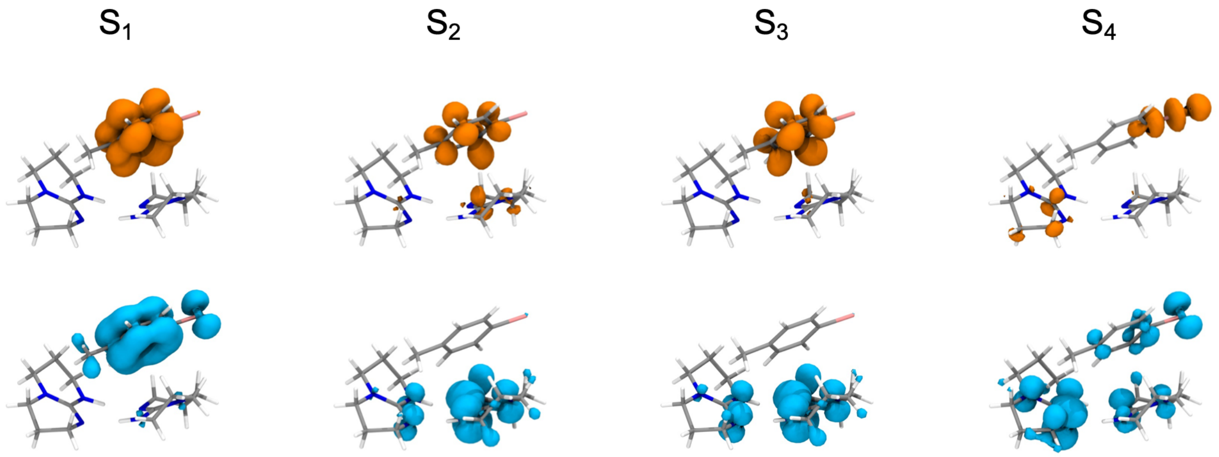

Figure S9. Electron/hole pair densities (orange/blue) for the S_1-S_4 states of EDA1 as obtained at the ground state geometry at the CAM-B3LYP/6-311+G(d,p) level in acetonitrile.

Table S8. Vertical de-excitation energies (in eV) from the S_1 excited state and associated oscillator strengths (*f*) computed at the CAM-B3LYP-D3/6-311+G(d,p) level in acetonitrile.

	**ΔE**	***f***
TBD	4.515	0.003
TBD-TBD	4.410	0.005
EDA5	3.596	0.010

10. References

1. Chavda, H. P.; Liang, K. J.; Megna, T. J.; Taylor, O. R.; Kim, G.; López, A. L.; Bahamonde, A. Indole photocatalysts and secondary amine ligands enable nickel-photoredox C(sp^2)–heteroatom couplings. *ACS Catal.* **2025**, *15*(12), 10760–10767. https://doi.org/10.1021/acscatal.5c03251
2. Fulmer, G. R.; Miller, A. J. M.; Sherden, N. H.; Gottlieb, H. E.; Nudelman, A.; Stoltz, B. M.; Bercaw, J. E.; Goldberg, K. I. NMR Chemical Shifts of Trace Impurities: Common Laboratory Solvents, Organics, and Gases in Deuterated Solvents Relevant to the Organometallic Chemist. *Organometallics* **2010**, *29*, 2176–2179. https://doi.org/10.1021/om100106e.
3. Pradhan, S.; Hu, J.; Ren, P.; Qin, Y.; Jain, N.; Monti, S.; Barcaro, G.; Jaworski, A.; Dai, X.; Jabor Rabeah; Silvestre-Albero, J.; Celorrio, V.; Rokicińska, A.; Piotr Kuśtrowski; Aert, S. V.; Bals, S.; Das, S. An Atomically Dispersed Photocatalyst for Undirected *para*-Selective C─H Bond Functionalizations. *Angew. Chem. Int. Ed.* **2025**, *64* (33), e202508512. https://doi.org/10.1002/anie.202508512.
4. Zhang, C.; Feng, P.; Jiao, N. Cu-Catalyzed Esterification Reaction via Aerobic Oxygenation and C–C Bond Cleavage: An Approach to α-Ketoesters. *J. Am. Chem. Soc.* **2013**, *135* (40), 15257–15262. https://doi.org/10.1021/ja4085463.
5. You, T.; Wang, Z.; Chen, J.; Xia, Y. Transfer Hydro-Dehalogenation of Organic Halides Catalyzed by Ruthenium(II) Complex. *J. Org. Chem.* **2017**, *82* (3), 1340–1346. https://doi.org/10.1021/acs.joc.6b02222.
6. Zimmermann, B. M.; Ngoc, T. T.; Tzaras, D.-I.; Kaicharla, T.; Teichert, J. F. A Bifunctional Copper Catalyst Enables Ester Reduction with H_2: Expanding the Reactivity Space of Nucleophilic Copper Hydrides. *J. Am. Chem. Soc* **2021**, *143* (40), 16865–16873. https://doi.org/10.1021/jacs.1c09626.
7. Yan, Z.; Yuan, X.-A.; Zhao, Y.; Zhu, C.; Xie, J. Selective Hydroarylation of 1,3-Diynes Using a Dimeric Manganese Catalyst: Modular Synthesis of *Z* -Enynes. *Angew. Chem. Int. Ed.* **2018**, *57* (39), 12906–12910. https://doi.org/10.1002/anie.201807851.
8. Shirai, T.; Sugimoto, K.; Iwasaki, M.; Sumida, R.; Fujita, H.; Yamamoto, Y. Decarbonylation through Aldehydic C–H Bond Cleavage by a Cationic Iridium Catalyst. *Synlett* **2019**, *30* (08), 972–976. https://doi.org/10.1055/s-0037-1611802.

9. Yamada, K.; Pak, K.; Gevorgyan, V. General Regio- and Diastereoselective Allylic C–H Oxygenation of Internal Alkenes. *J. Am. Chem. Soc* **2024**, *146* (27), 18218–18223. https://doi.org/10.1021/jacs.4c06421.
10. Majek, M.; Jacobi von Wangelin, A. Metal-Free Carbonylations by Photoredox Catalysis. *Angew. Chem. Int. Ed.* **2014**, *54* (7), 2270–2274. https://doi.org/10.1002/anie.201408516.
11. Krüger, T.; Vorndran, K.; Linker, T. Regioselective Arene Functionalization: Simple Substitution of Carboxylate by Alkyl Groups. *Chemistry* **2009**, *15* (44), 12082–12091. https://doi.org/10.1002/chem.200901774.
12. Wu, S., Schiel, F., & Melchiorre, P. (2023). A General Light-Driven Organocatalytic Platform for the Activation of Inert Substrates. *Angew. Chem. Int. Ed.,* **2023**, *135* (32), e202306364. https://onlinelibrary.wiley.com/doi/10.1002/anie.202306364
13. Mallik, S.; Wang, H.; Matera, N.; Li, B.; Stagni, S.; Melchiorre, P. A Bifunctional Organic Photocatalyst for Efficient Single-Electron and Energy Transfer Activation. *Angew. Chem. Int. Ed.* **2025**, *64* (35). https://doi.org/10.1002/anie.202509770.
14. Yoo, B. I.; Kim, Y. J.; You, Y.; Yang, J. W.; Kim, S. W. Birch Reduction of Aromatic Compounds by Inorganic Electride $[Ca_2N]^{+\bullet}E^{-}$ in an Alcoholic Solvent: An Analogue of Solvated Electrons. *J. Org. Chem.* **2018**, *83* (22), 13847–13853. https://doi.org/10.1021/acs.joc.8b02094.
15. Grimme, S.; Hansen, A.; Brandenburg, J. G.; Bannwarth, C. Dispersion-Corrected Mean-Field Electronic Structure Methods. *Chem. Rev.* **2016**, *116* (9), 5105–5154. https://doi.org/10.1021/acs.chemrev.5b00533.
16. Tomasi, J.; Mennucci, B.; Cancès, E. The IEF version of the PCM solvation method: an overview of a new method addressed to study molecular solutes at the QM ab initio level. *THEOCHEM* **1999**, *464* (1), 211–226. https://doi.org/10.1016/S0166-1280(98)00553-3.
17. Cancès, E. Mennucci, B.; Tomasi, J. A new integral equation formalism for the polarizable continuum model: Theoretical background and applications to isotropic and anisotropic dielectrics. *J. Chem. Phys.* **1997**, *107* (8), 3032–3041. https://doi.org/10.1063/1.474659.
18. Cossi, M.; Rega, N.; Scalmani, G.; Barone, V. Energies, structures, and electronic properties of molecules in solution with the C-PCM solvation model. *J. Comput. Chem.* **2003**, *24* (6), 669–681. https://doi.org/10.1002/jcc.10189.

19. Barone, V.; Cossi, M. Quantum Calculation of Molecular Energies and Energy Gradients in Solution by a Conductor Solvent Model. *J. Phys. Chem. A* **1998**, *102* (11), 1995–2001. https://pubs.acs.org/doi/10.1021/jp9716997.

20. Frisch, M.J., Trucks, G.W., Schlegel, H.B., et al. *Gaussian 16, Revision B.01*. Gaussian, Inc., Wallingford CT, 2016.

21. Epifanovsky, E.; Gilbert, A. T. B.; Feng, X.; Lee, J.; Mao, Y.; Mardirossian, N.; Pokhilko, P.; White, A. F.; Coons, M. P.; Dempwolff, A. L.; et al. Software for the frontiers of quantum chemistry: An overview of developments in the Q-Chem 5 package. *J. Chem. Phys.* **2021**, *155*, 084801. https://doi.org/10.1063/5.0055522

11. NMR Spectra

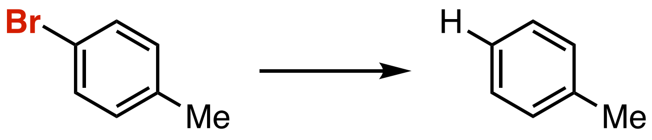

1a

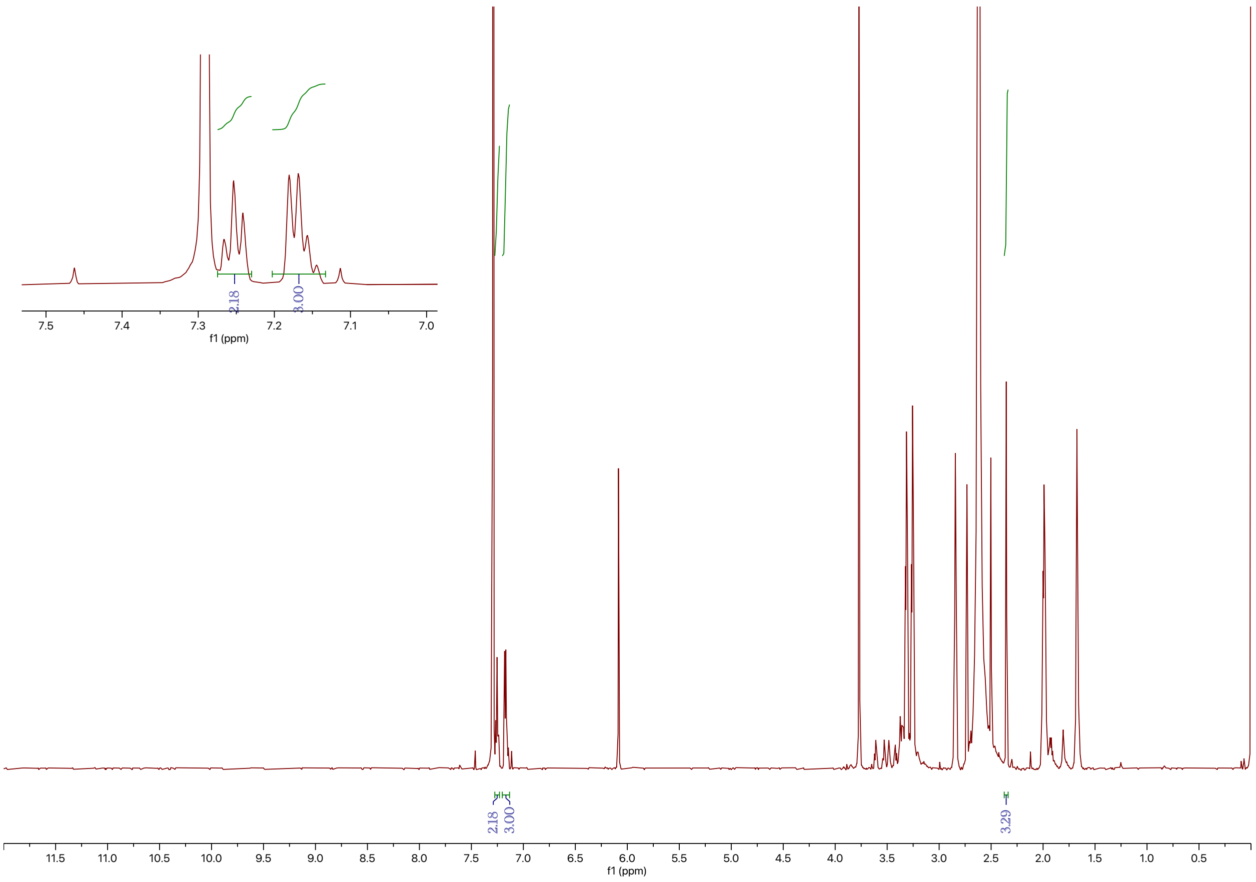

Crude reaction mixture - ^{1}H NMR (600 MHz, $CDCl_3$)

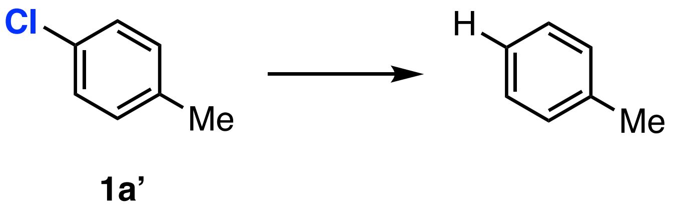

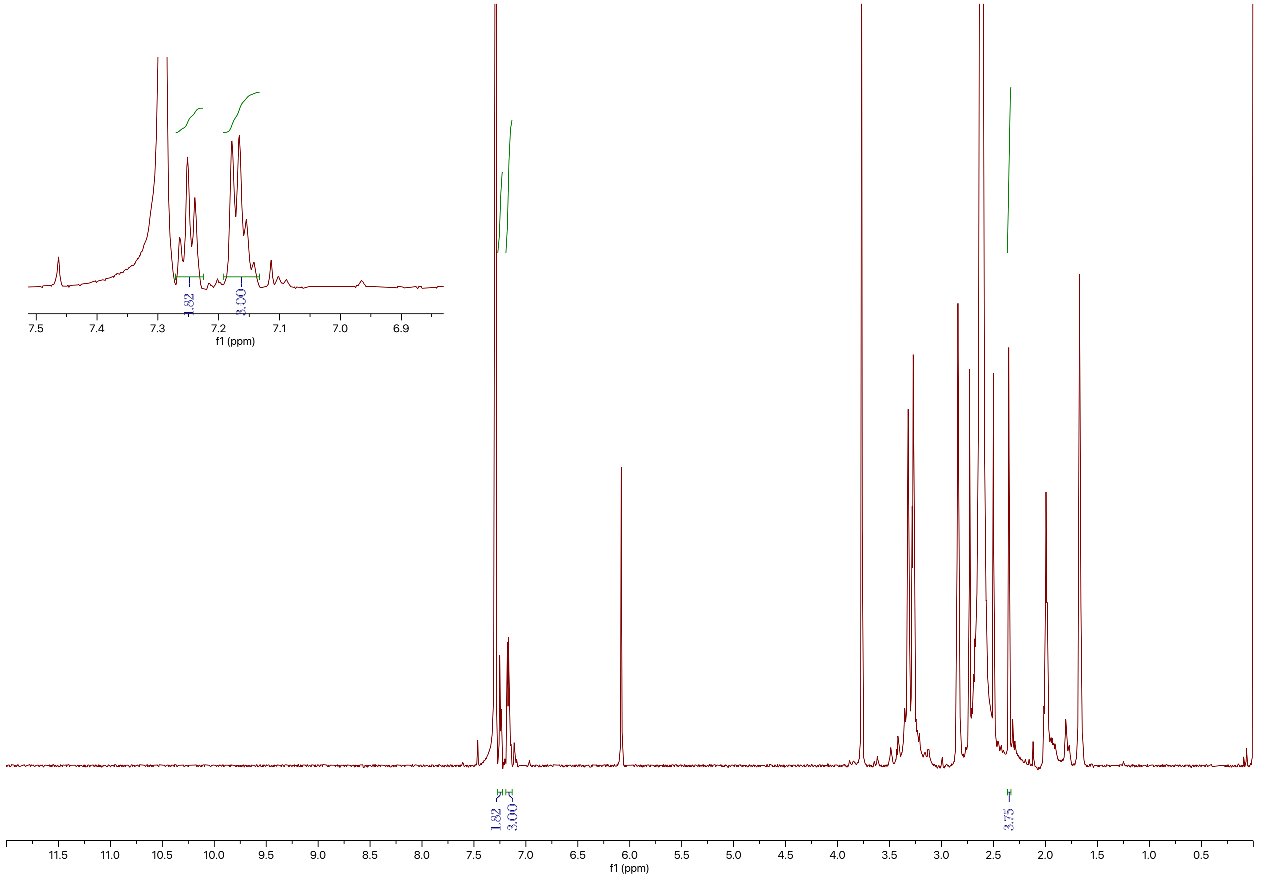

Crude reaction mixture - ^{1}H NMR (600 MHz, $CDCl_3$)

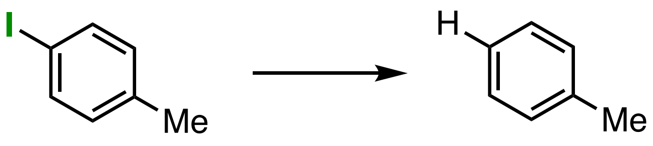

1a”

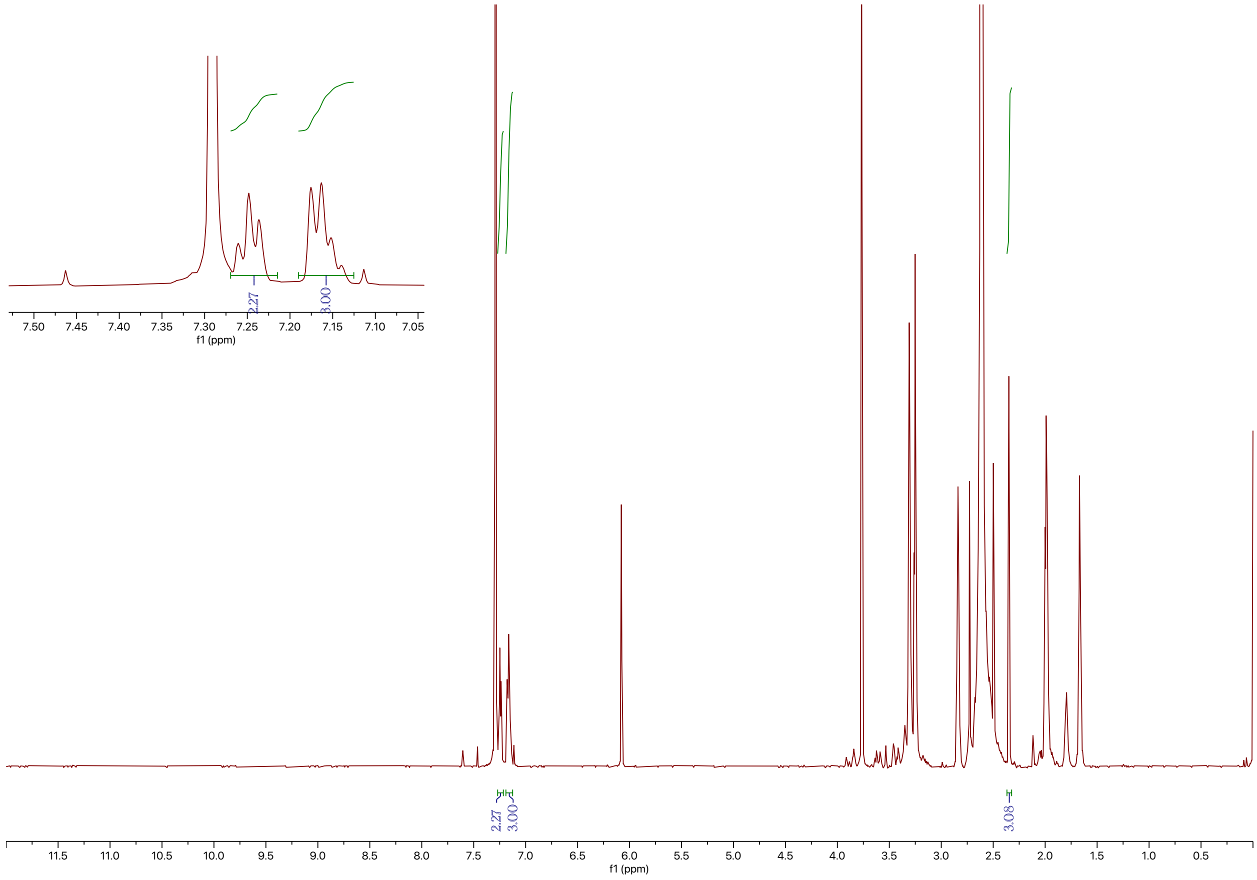

Crude reaction mixture - ^{1}H NMR (600 MHz, $CDCl_3$)

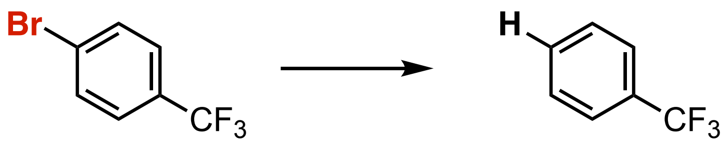

1b

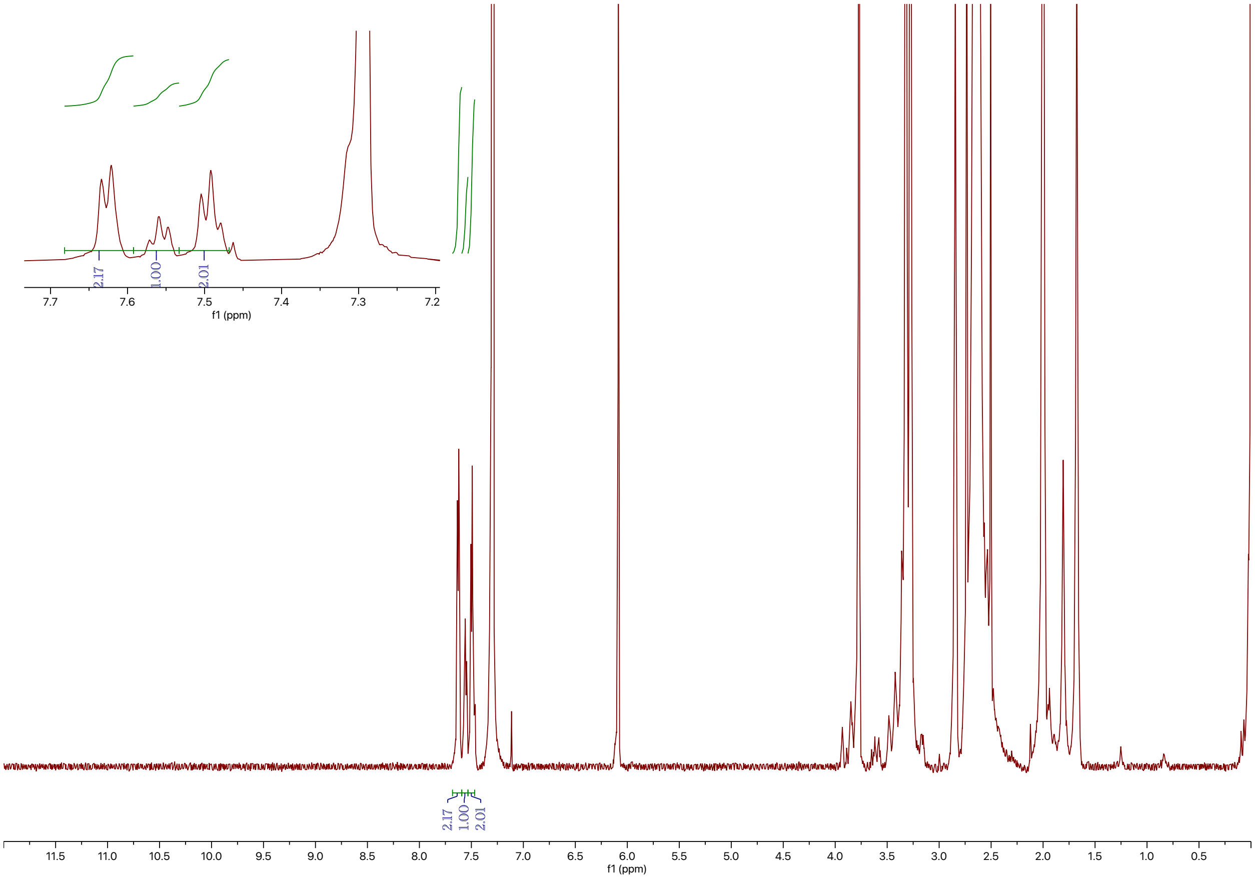

Crude reaction mixture - ^{1}H NMR (600 MHz, $CDCl_3$)

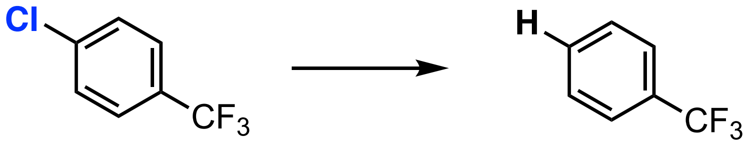

1b'

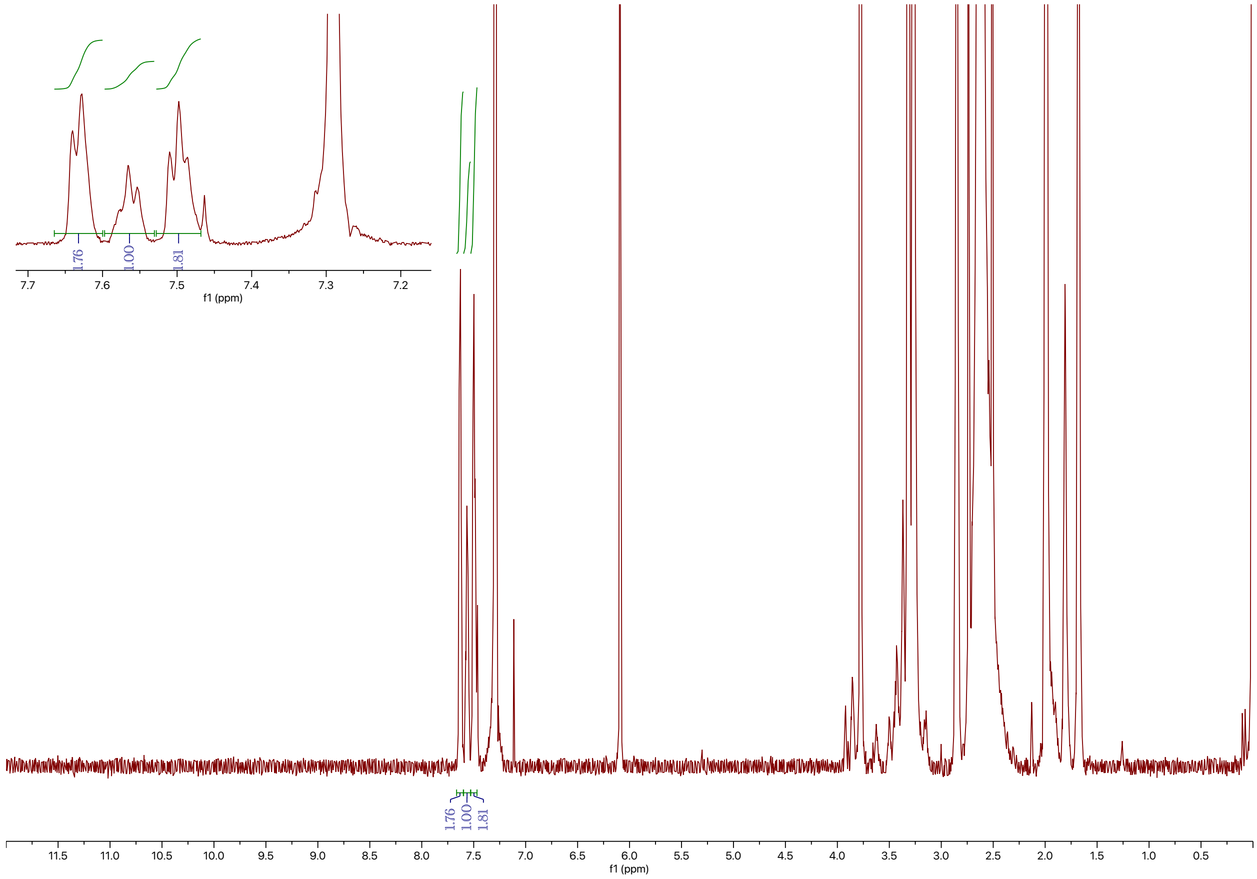

Crude reaction mixture - ^{1}H NMR (600 MHz, $CDCl_3$)

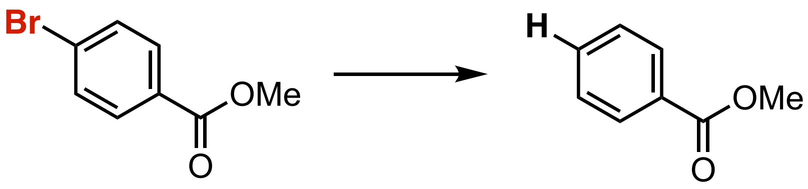

1c

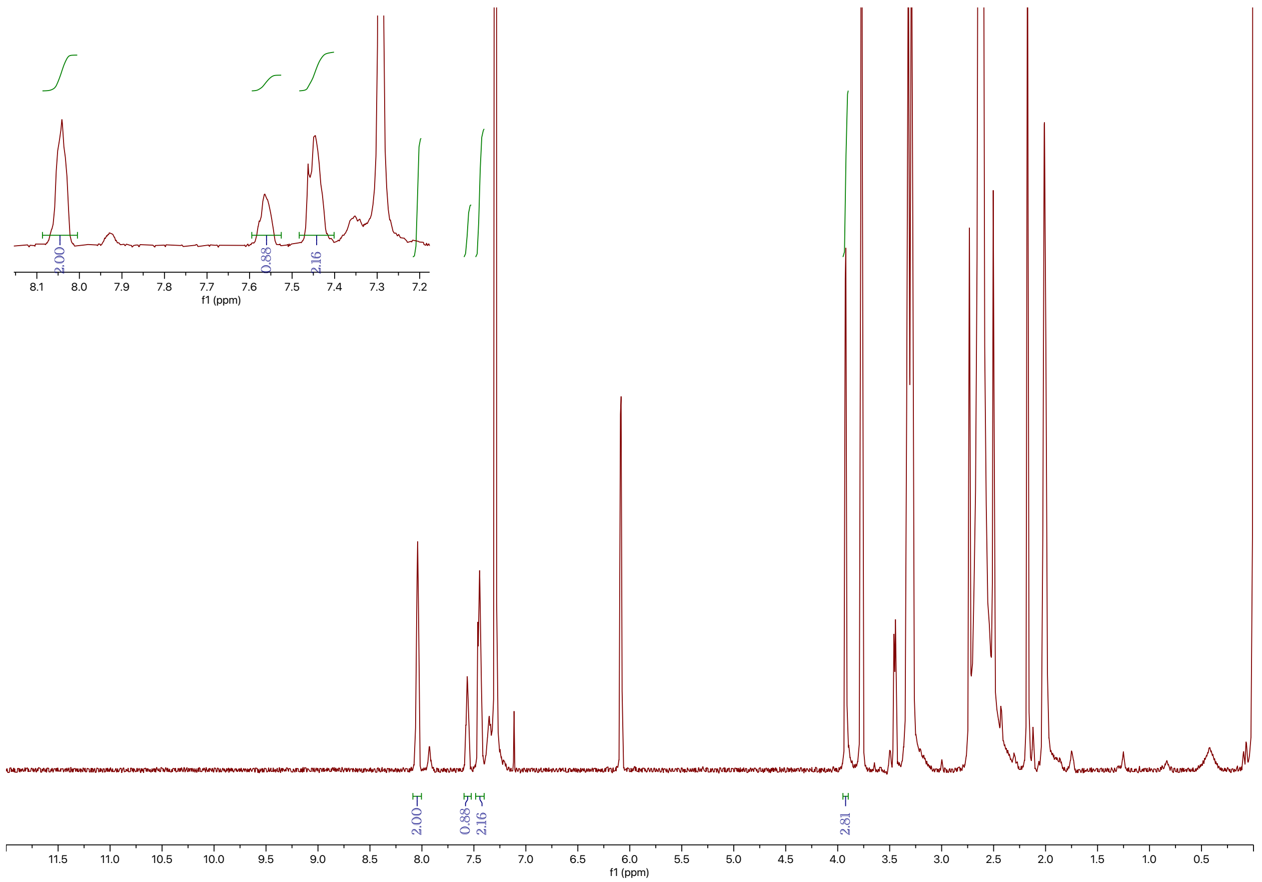

Crude reaction mixture - ^{1}H NMR (600 MHz, $CDCl_3$)

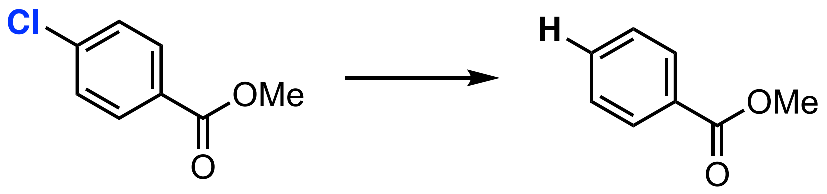

1c’

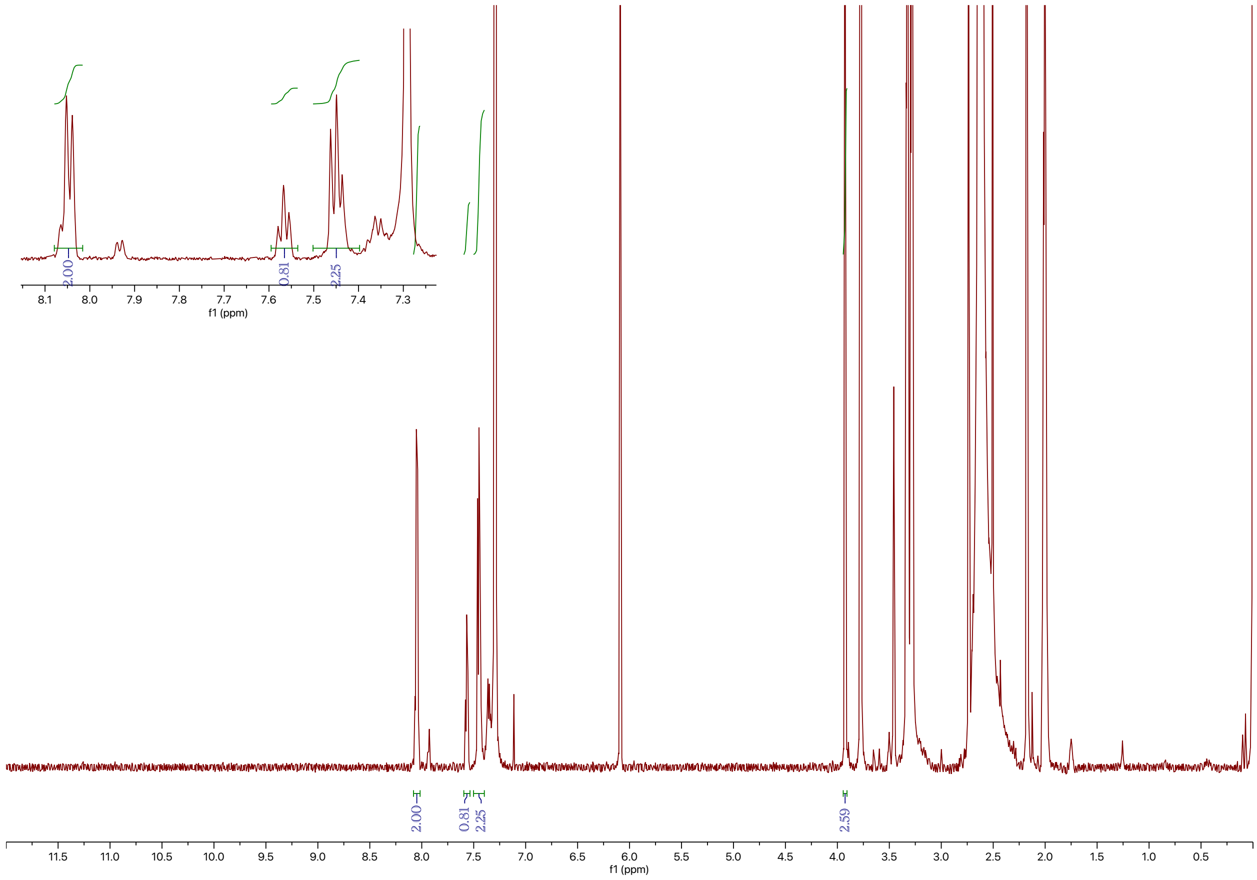

Crude reaction mixture - ^{1}H NMR (600 MHz, $CDCl_3$)

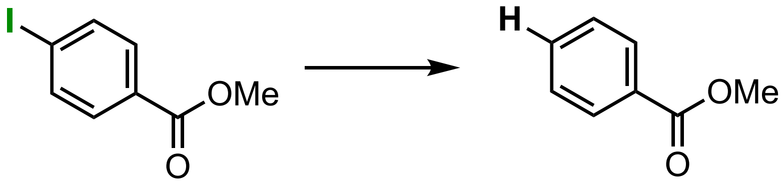

1c''

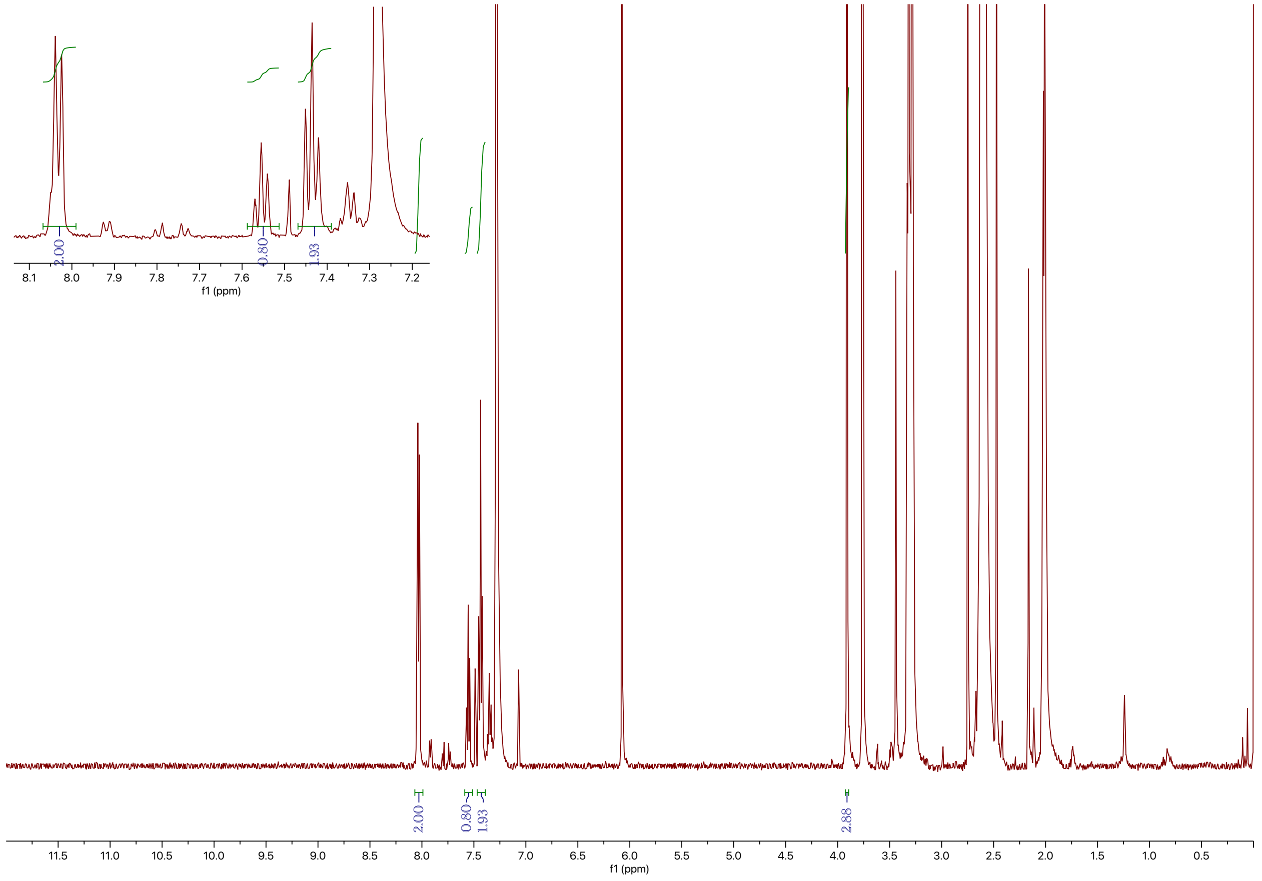

Crude reaction mixture - ^{1}H NMR (500 MHz, $CDCl_3$)

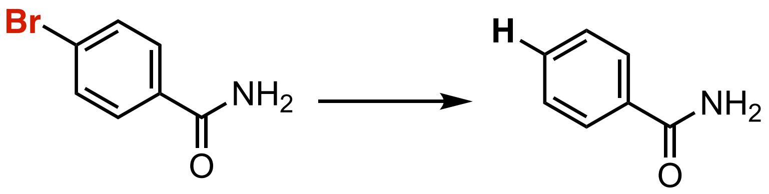

1d

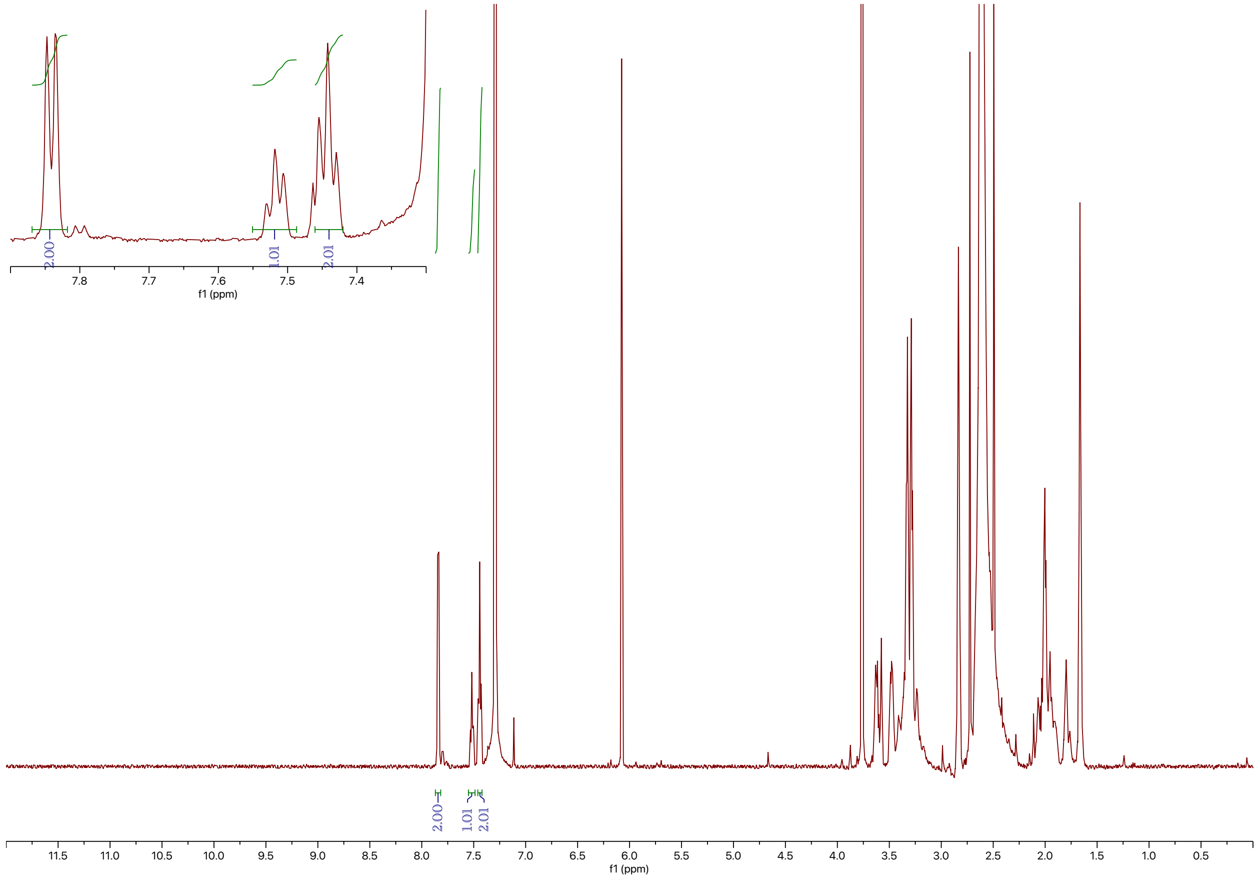

Crude reaction mixture - ^{1}H NMR (500 MHz, $CDCl_3$)

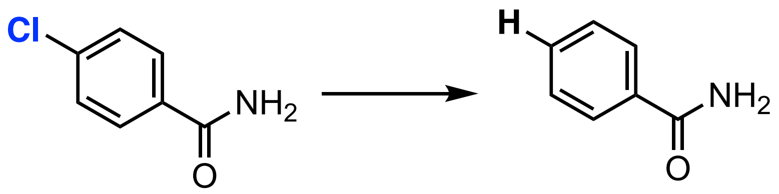

1d’

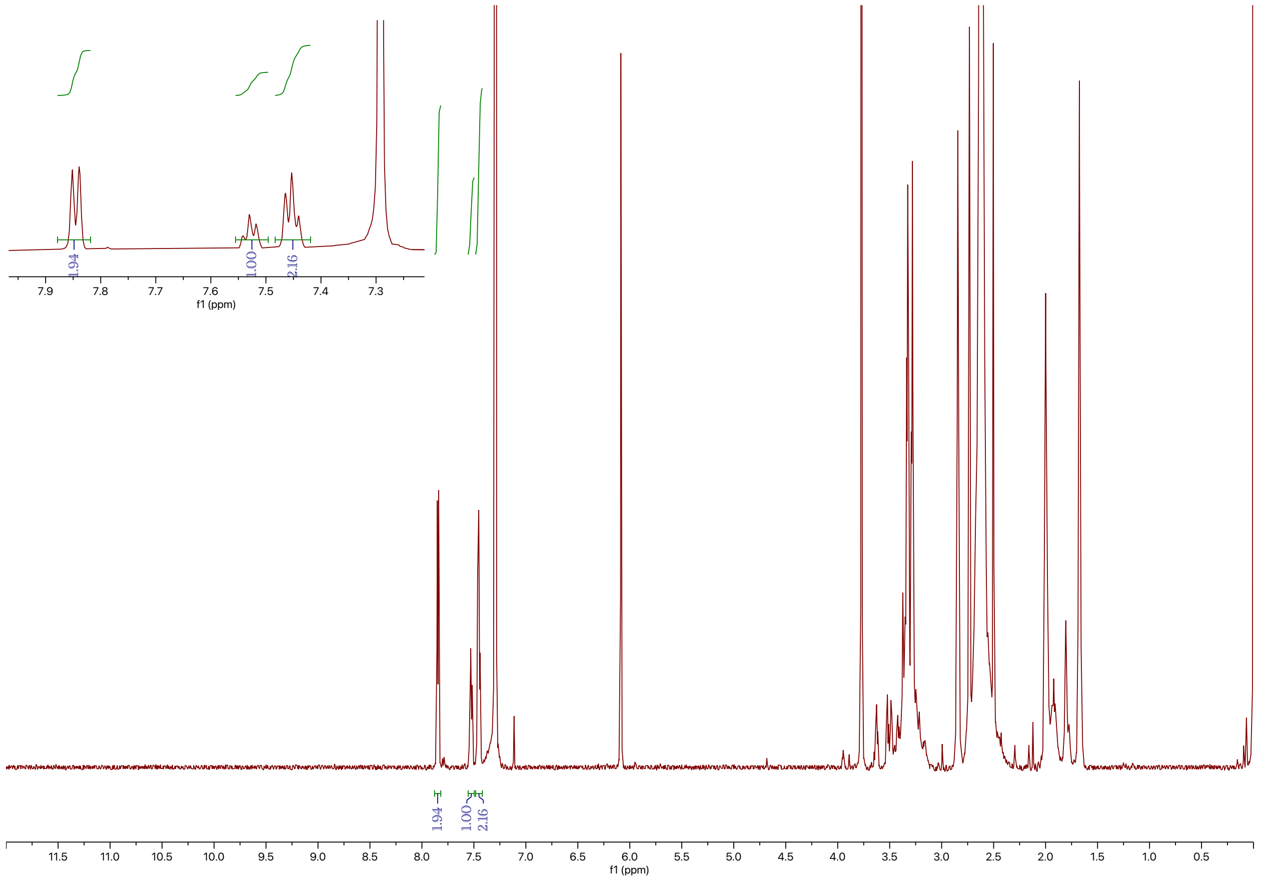

Crude reaction mixture - ^{1}H NMR (600 MHz, $CDCl_3$)

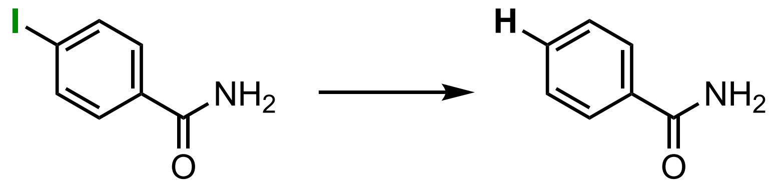

1d”

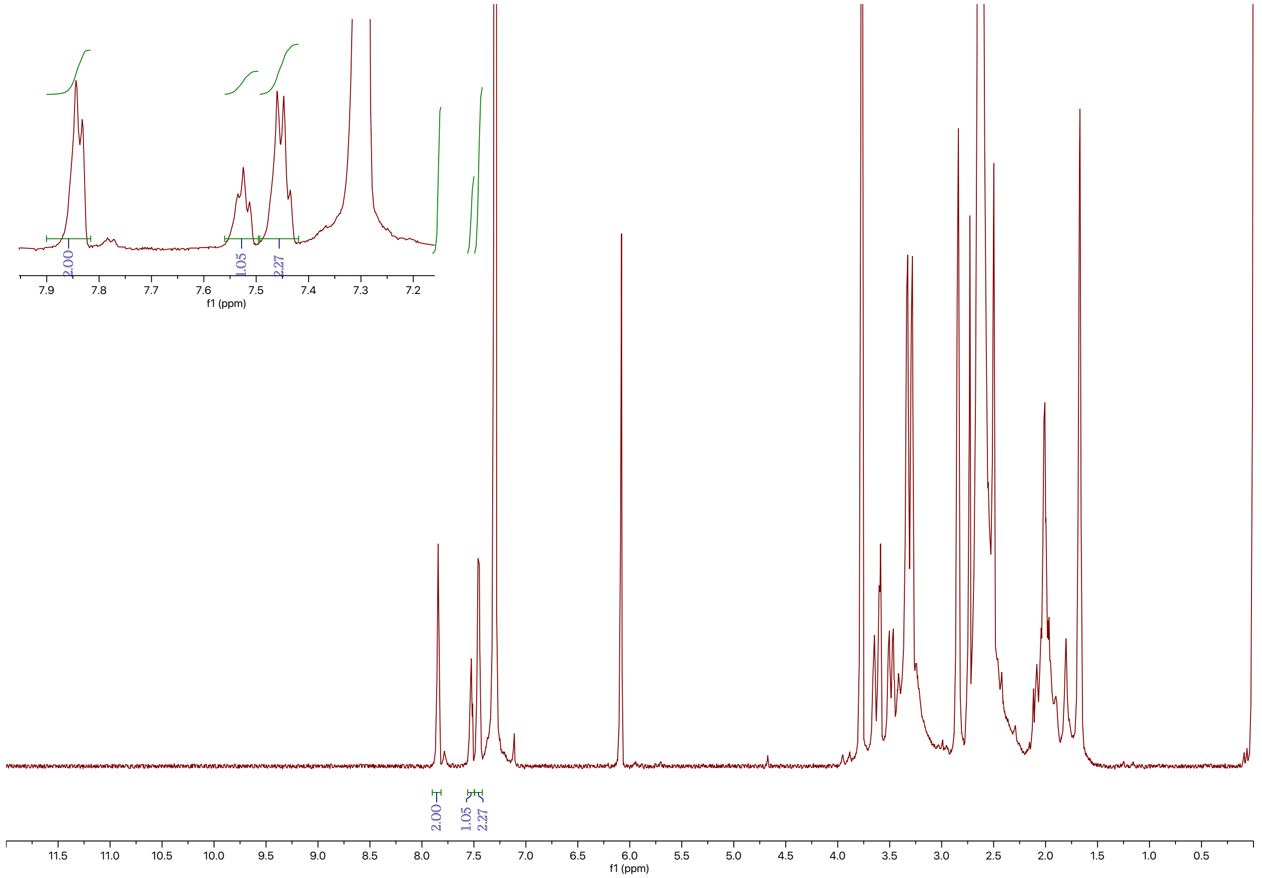

Crude reaction mixture - ^{1}H NMR (600 MHz, $CDCl_3$)

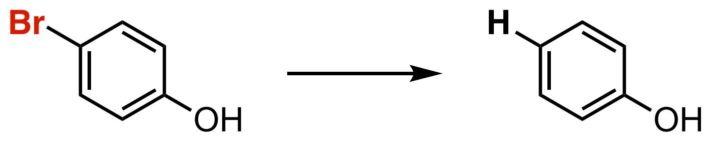

1e

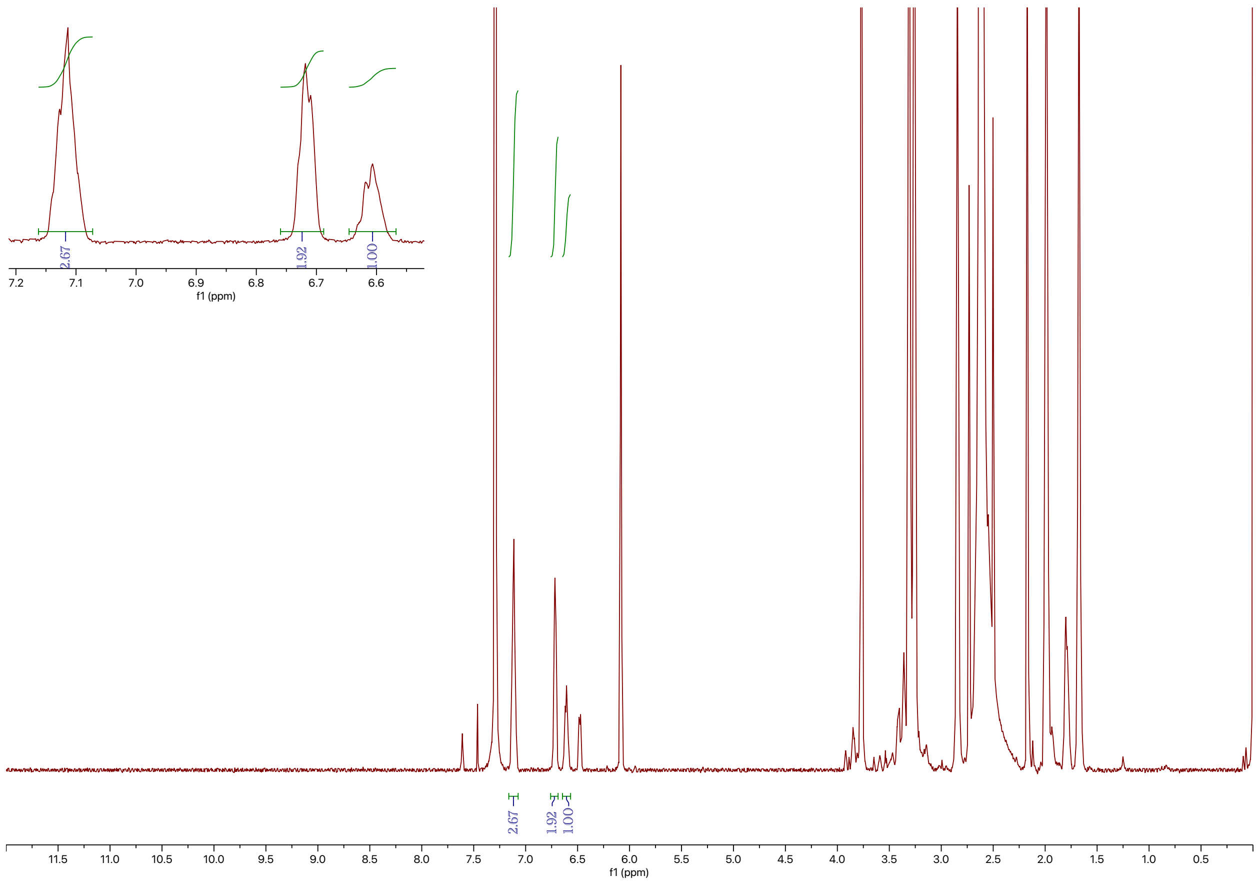

Crude reaction mixture - ^{1}H NMR (600 MHz, $CDCl_3$)

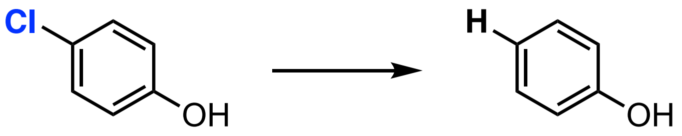

1e'

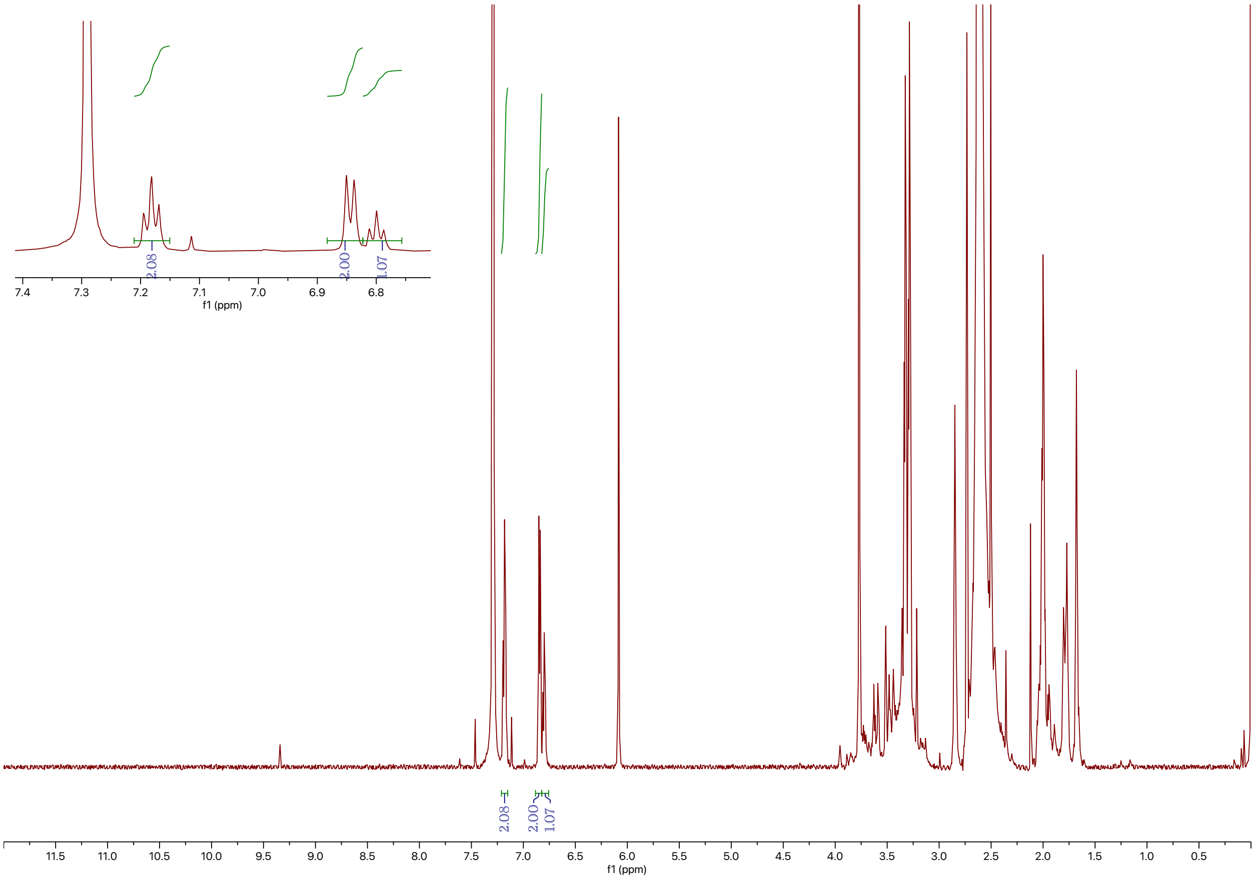

Crude reaction mixture - ^{1}H NMR (600 MHz, $CDCl_3$)

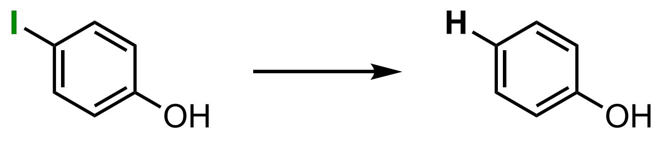

1e”

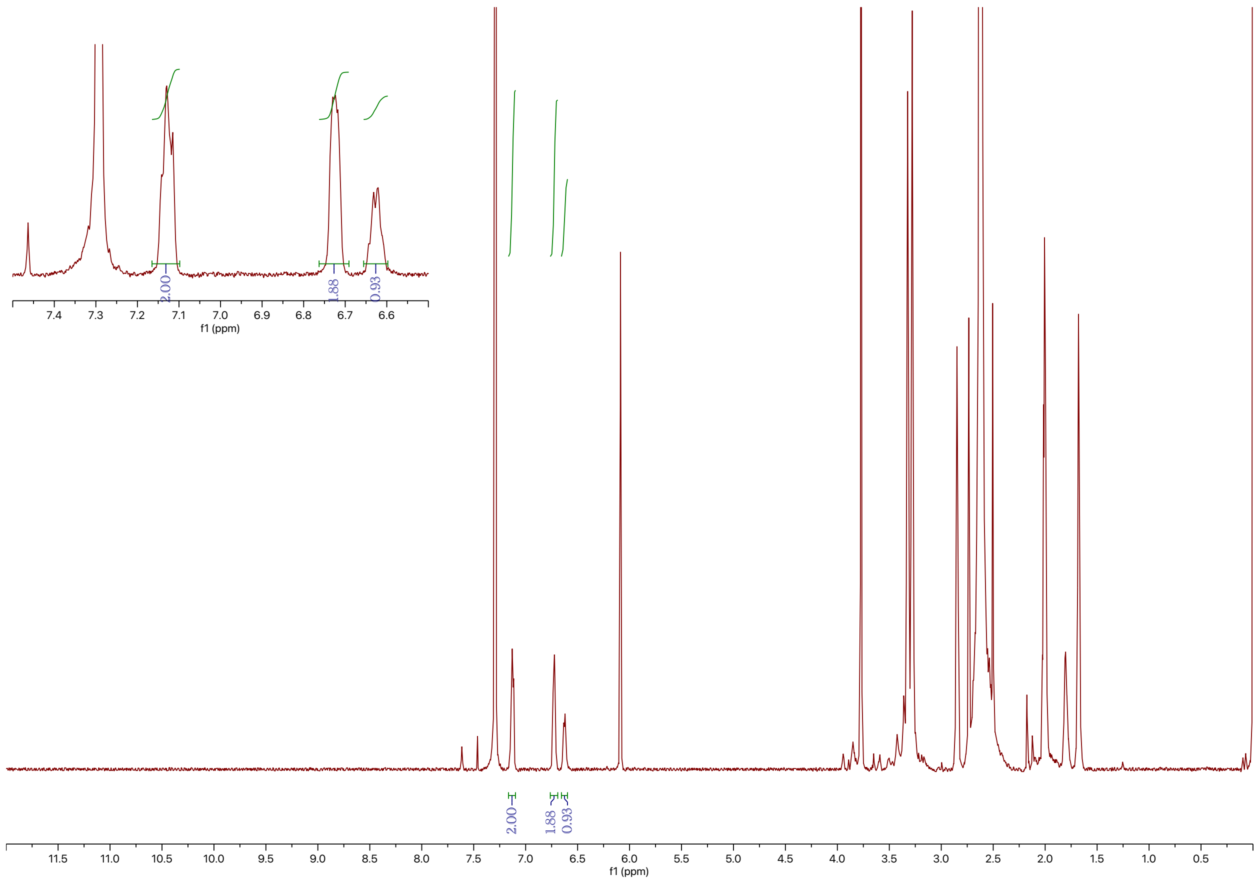

Crude reaction mixture - ^{1}H NMR (600 MHz, $CDCl_3$)

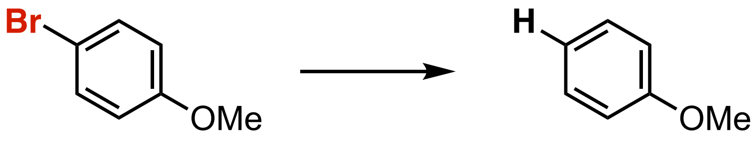

1f

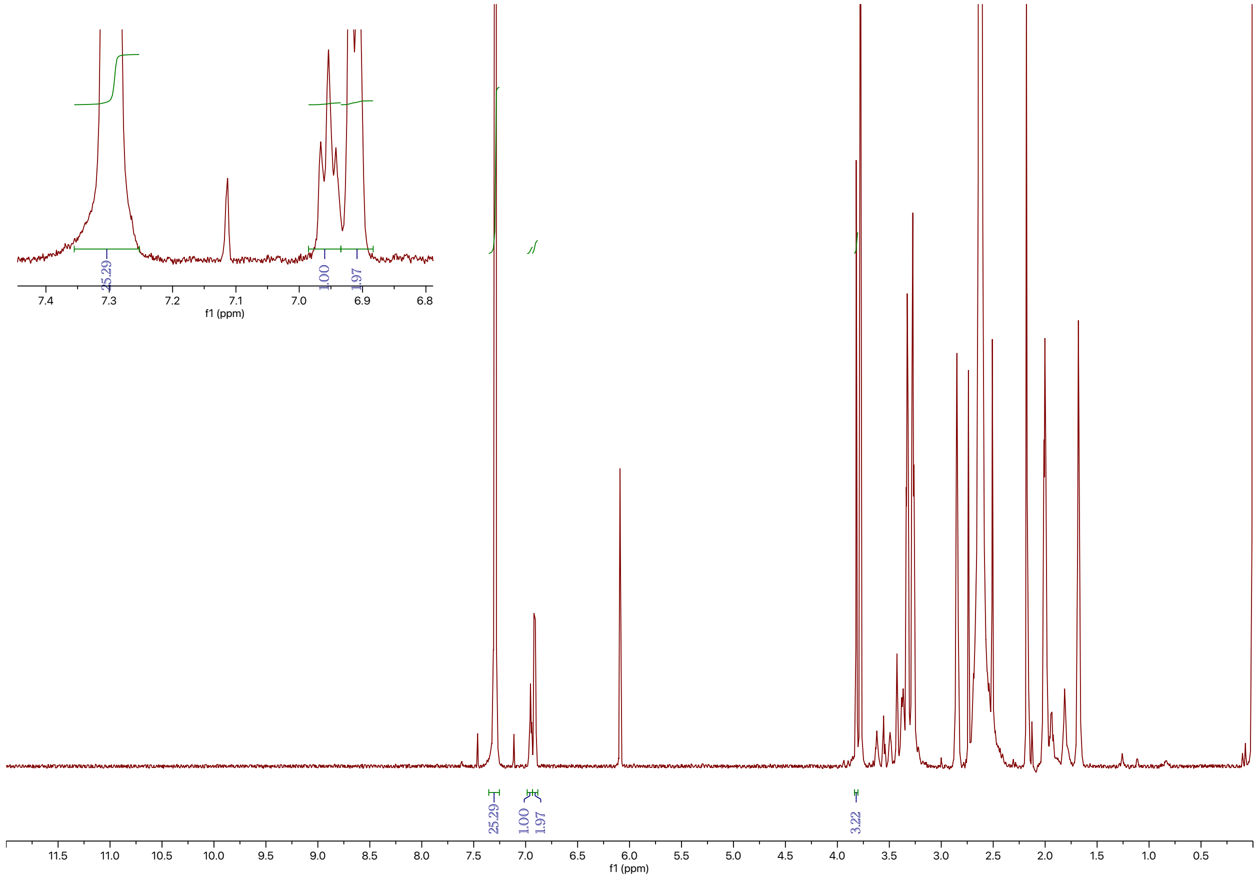

Crude reaction mixture - ^{1}H NMR (600 MHz, $CDCl_3$)

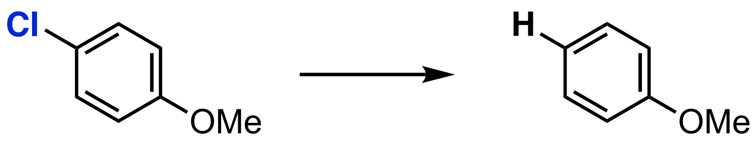

1f’

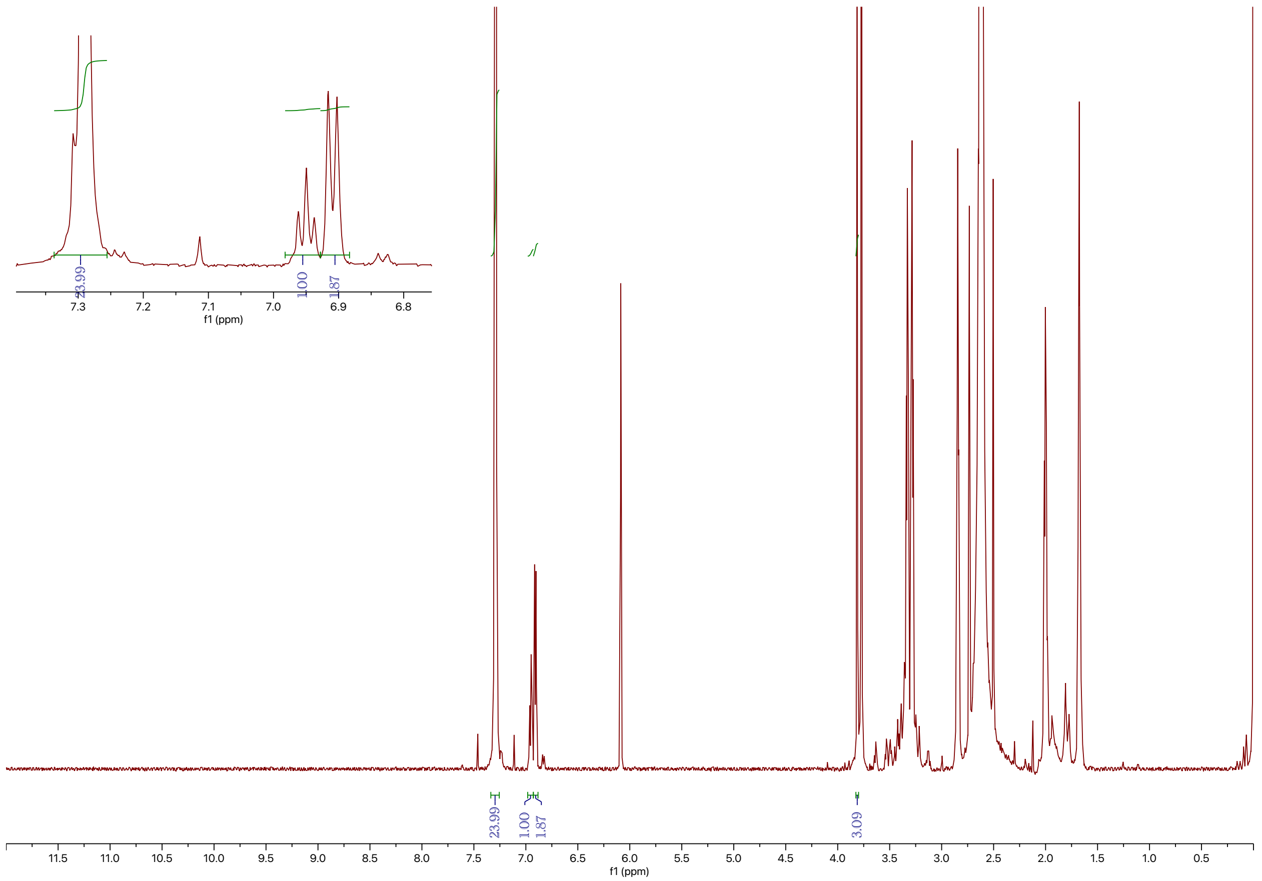

Crude reaction mixture - ^{1}H NMR (600 MHz, $CDCl_3$)

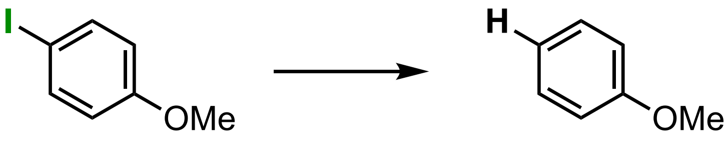

1f''

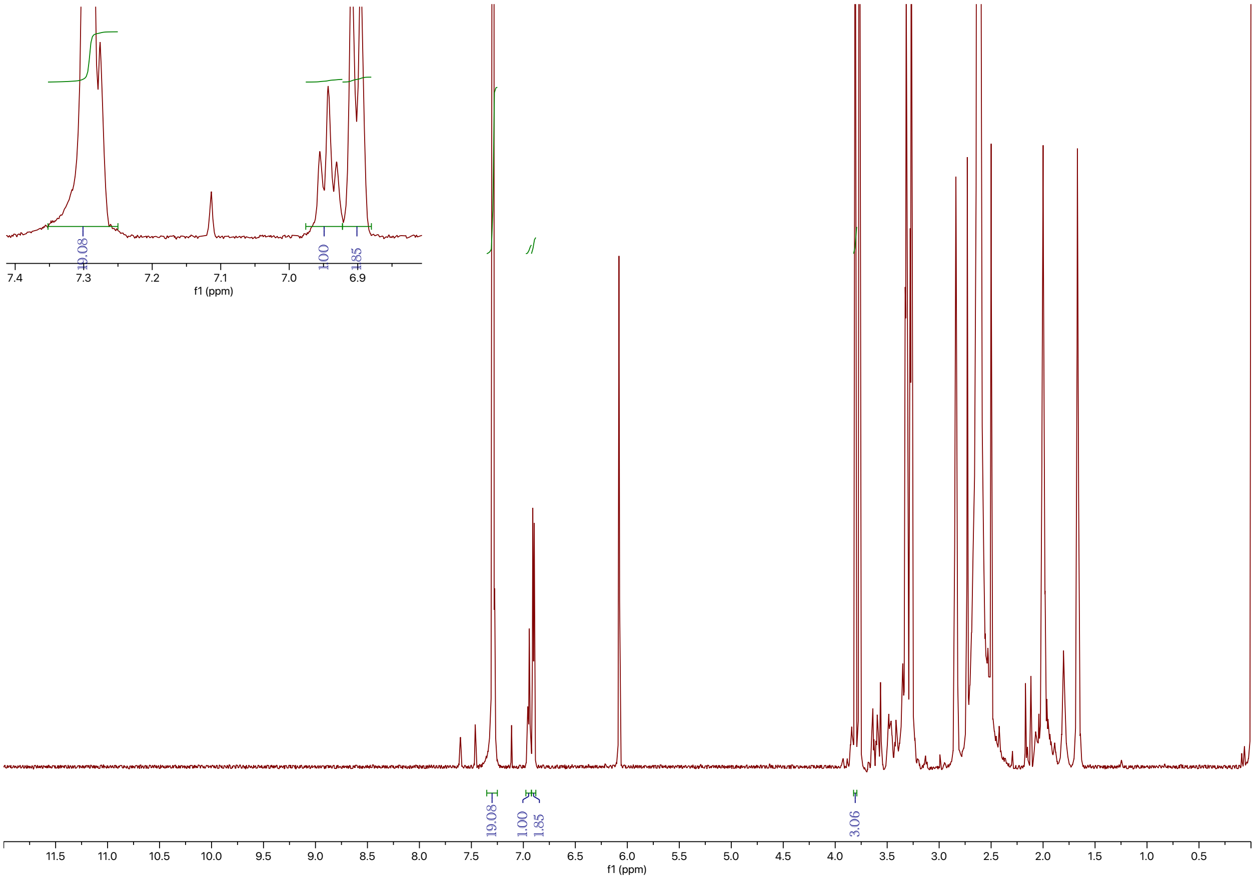

Crude reaction mixture - ¹H NMR (600 MHz, $CDCl_3$)

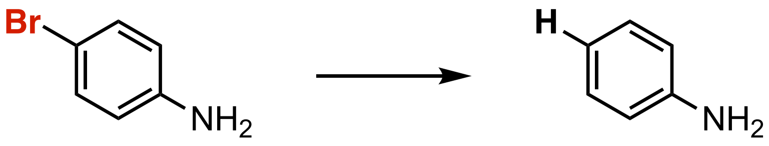

1g

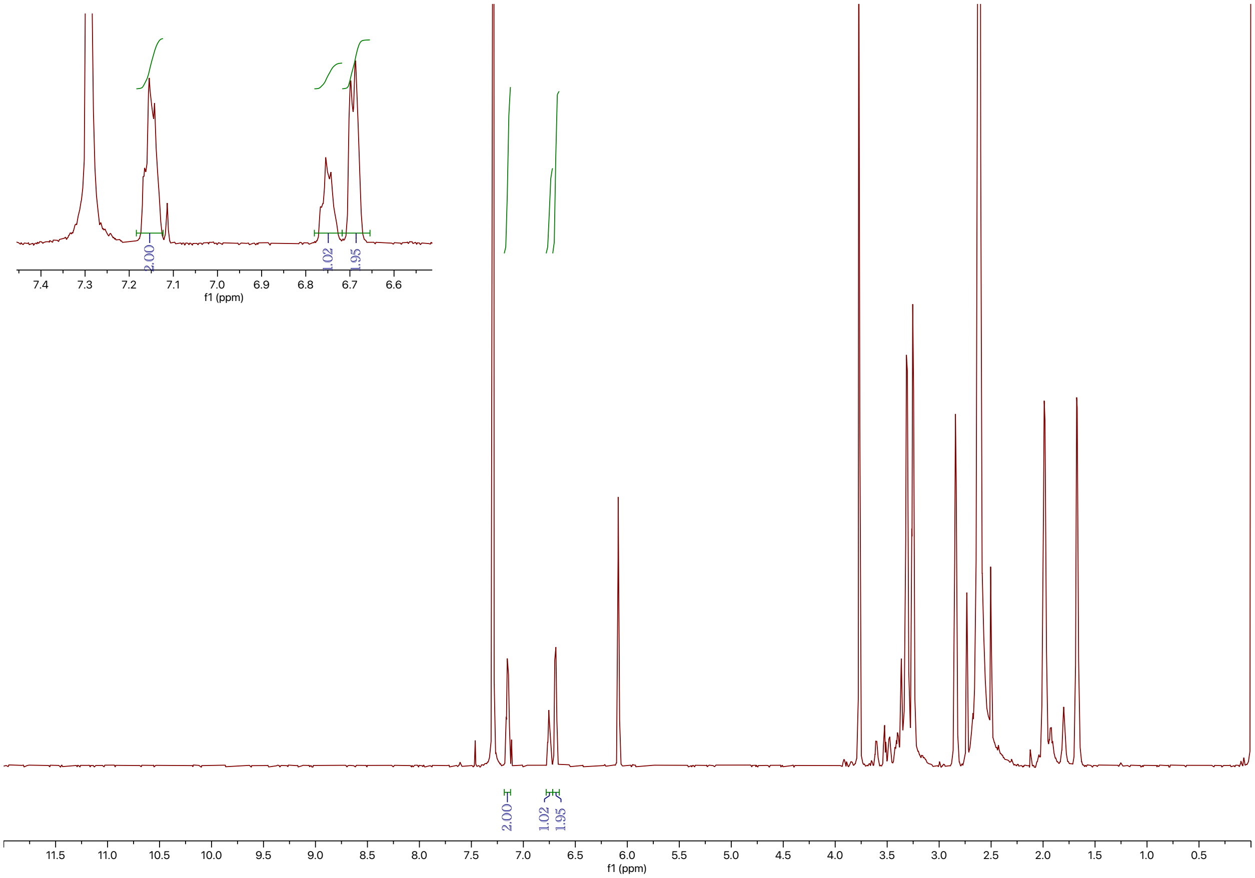

Crude reaction mixture - ^{1}H NMR (600 MHz, $CDCl_3$)

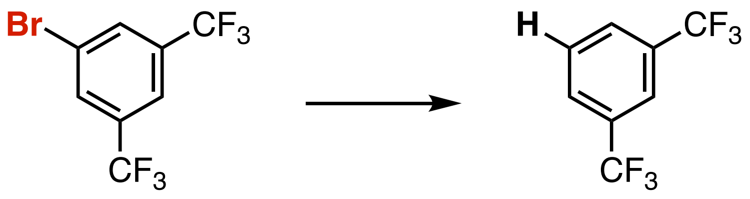

1h

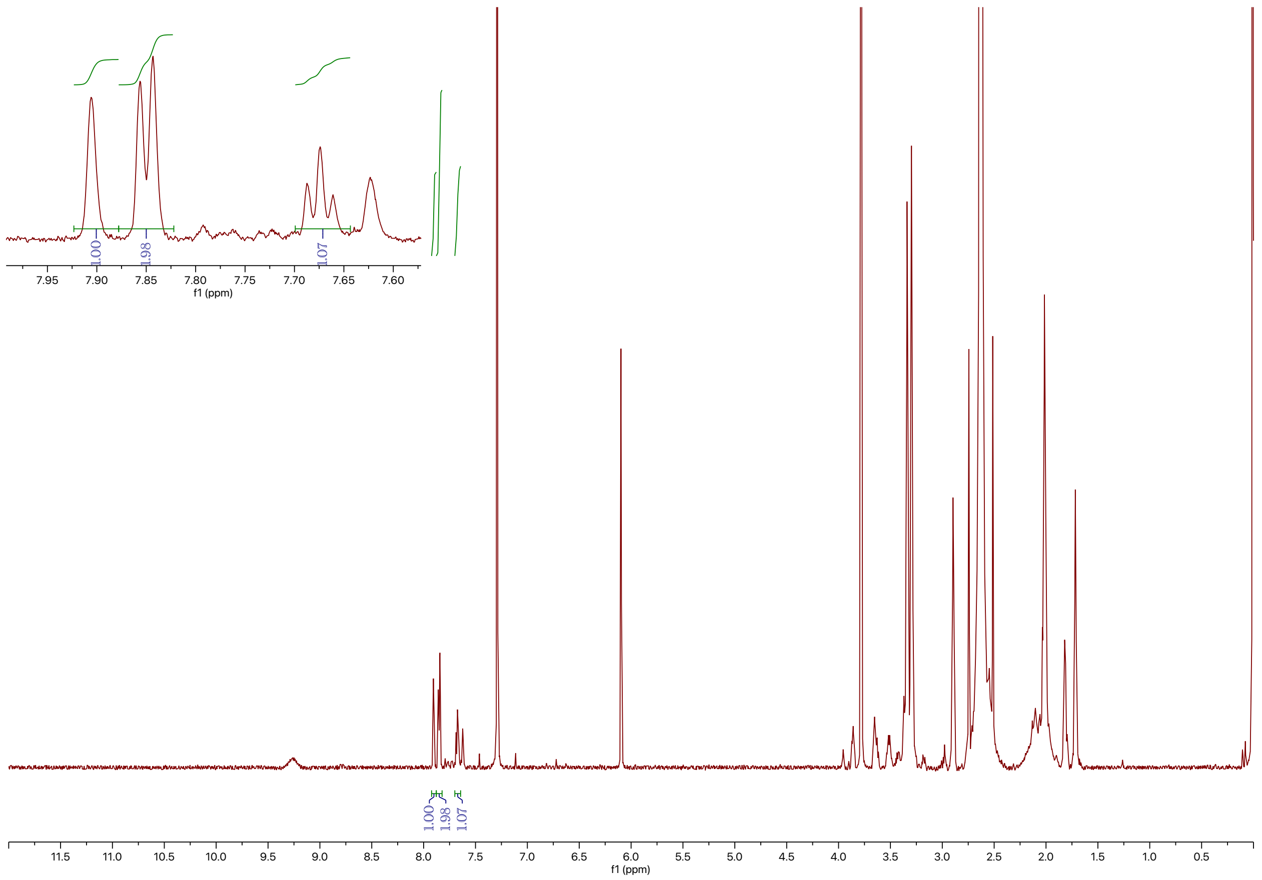

Crude reaction mixture - ^{1}H NMR (600 MHz, $CDCl_3$)

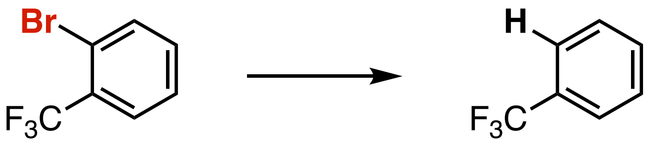

1i

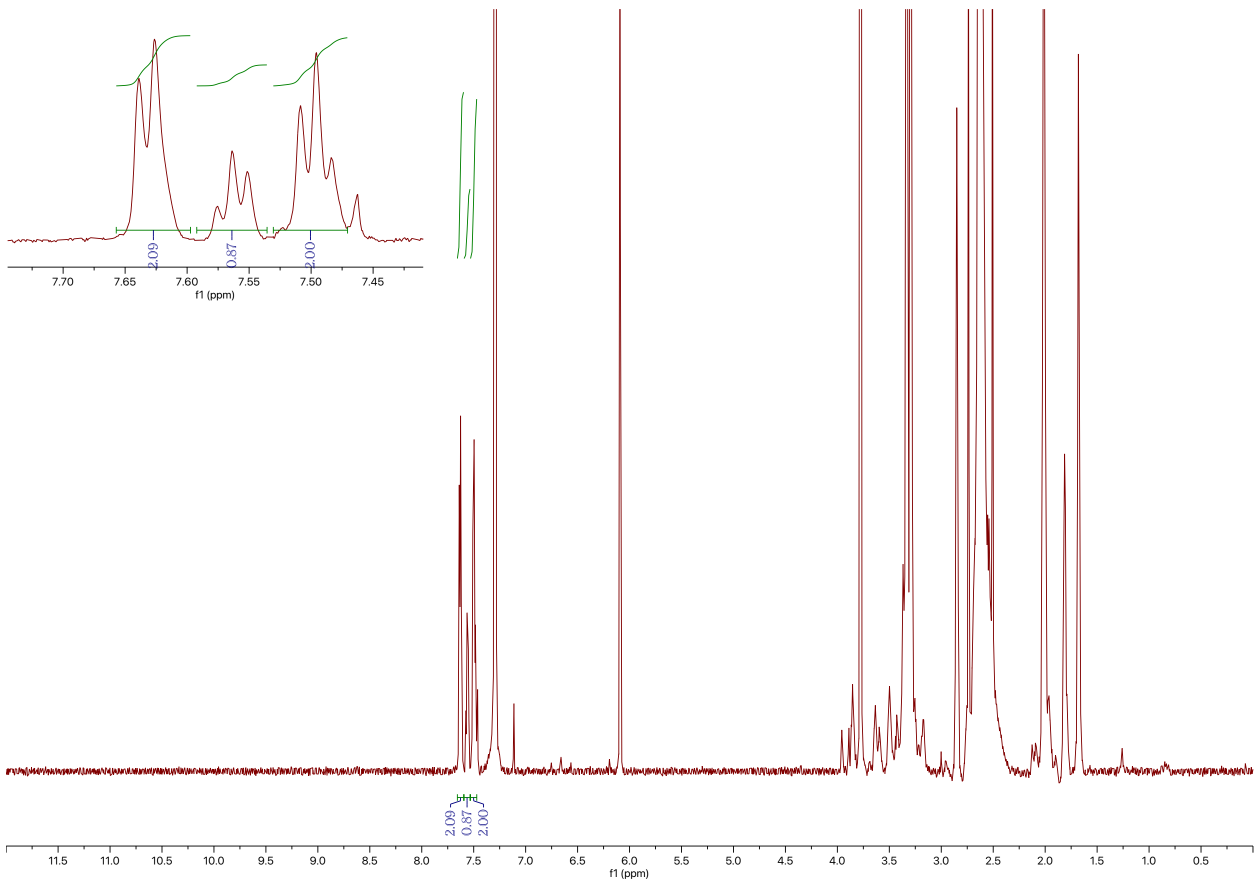

Crude reaction mixture - ^{1}H NMR (600 MHz, $CDCl_3$)

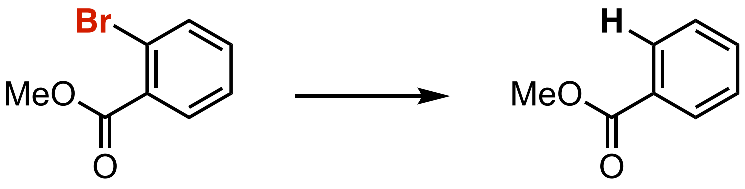

1j

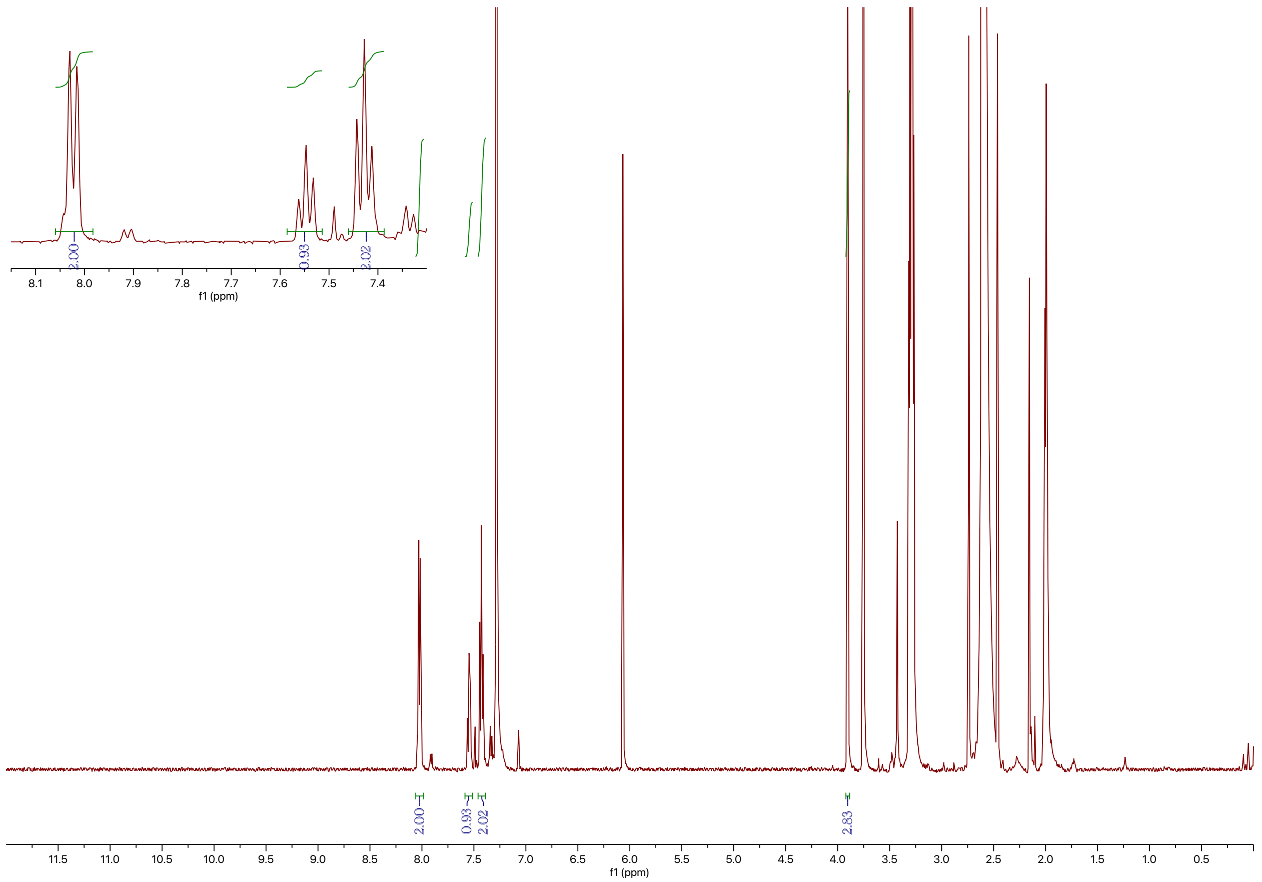

Crude reaction mixture - ^{1}H NMR (600 MHz, $CDCl_3$)

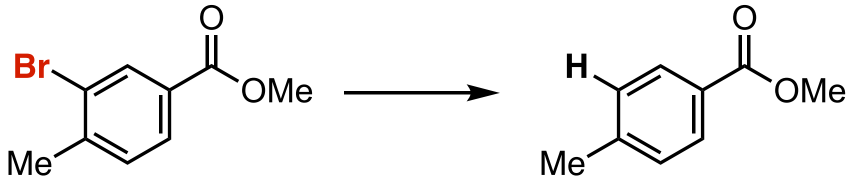

1k

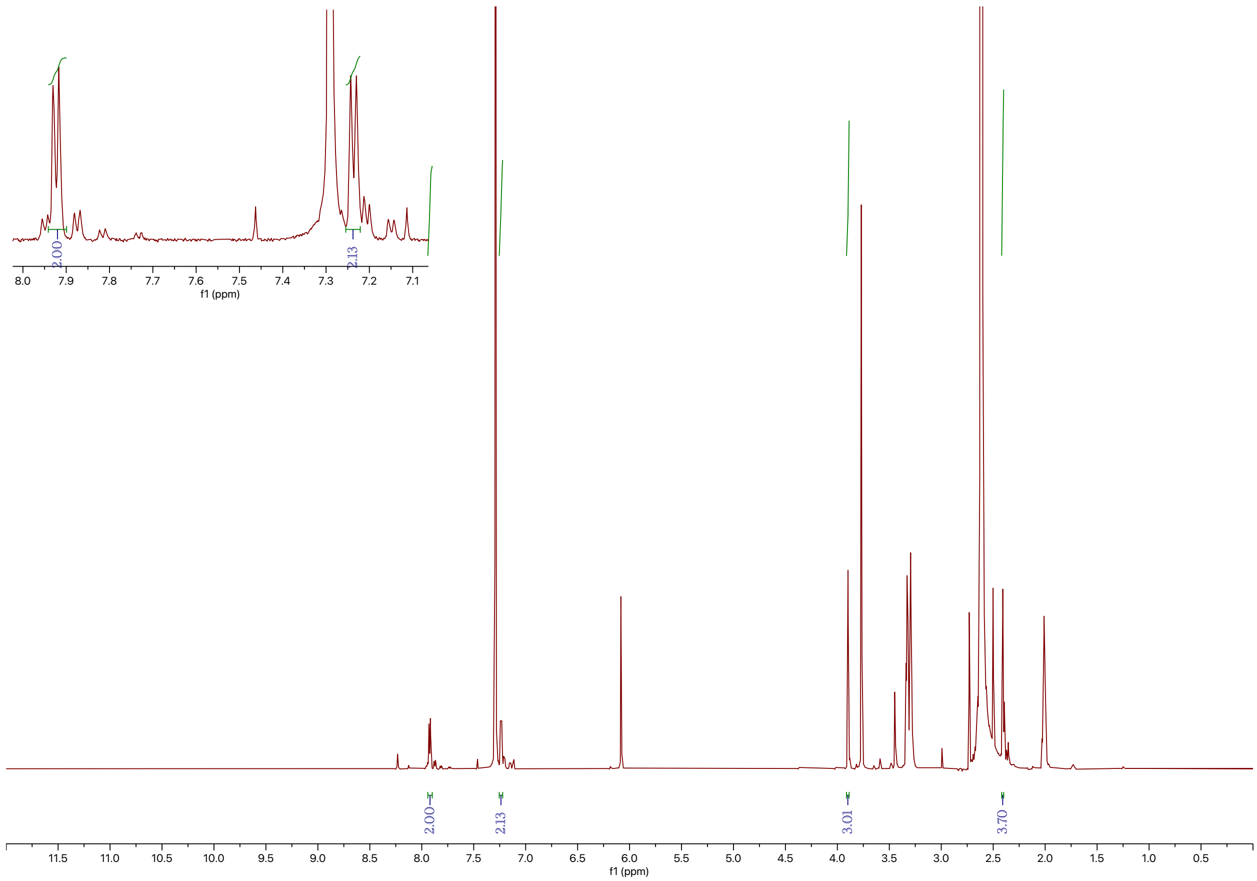

Crude reaction mixture - ^{1}H NMR (600 MHz, $CDCl_3$)

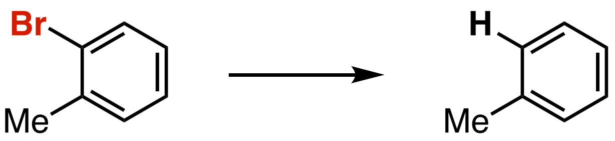

1l

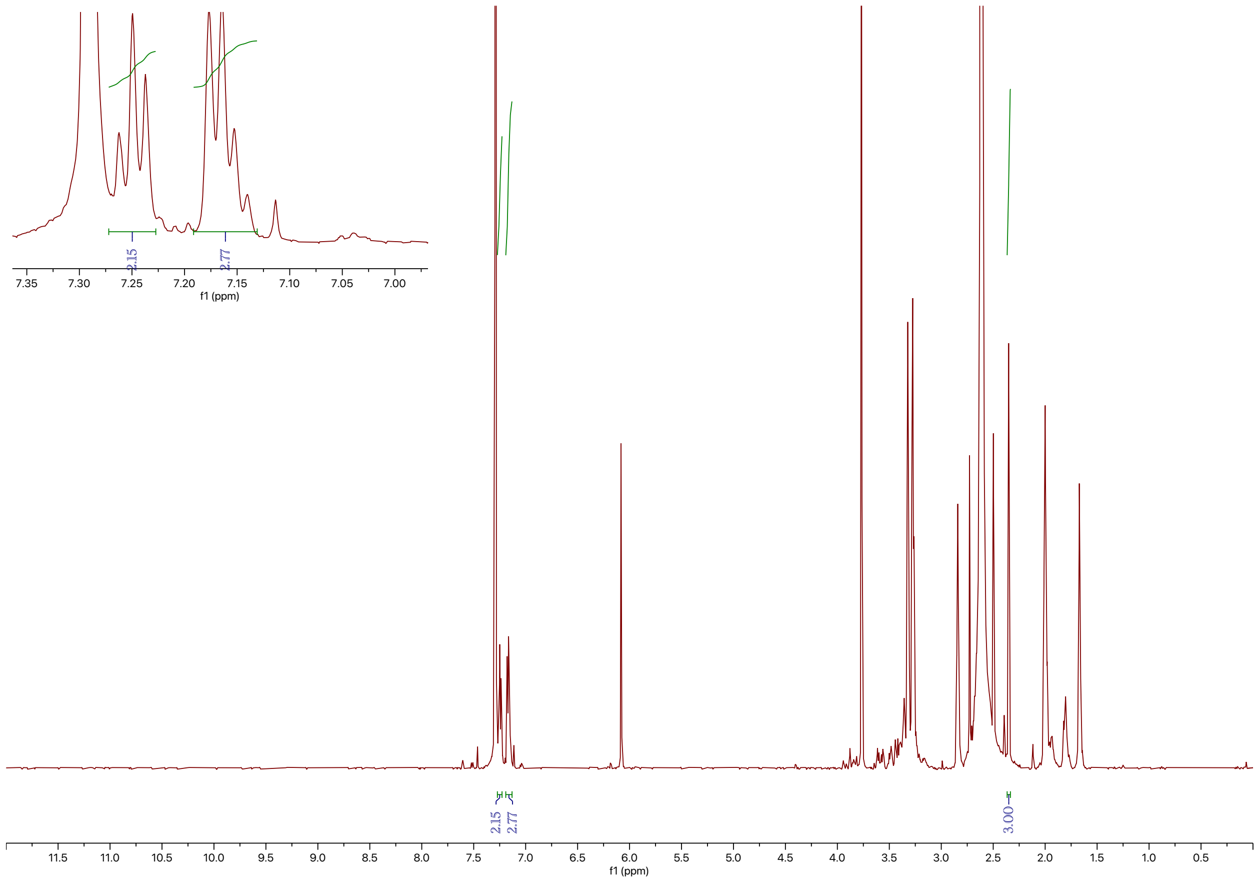

Crude reaction mixture - ^{1}H NMR (600 MHz, $CDCl_3$)

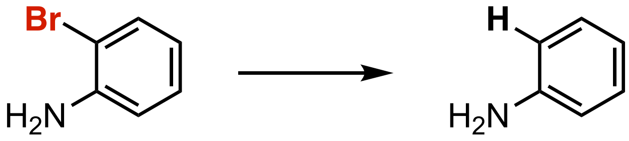

1m

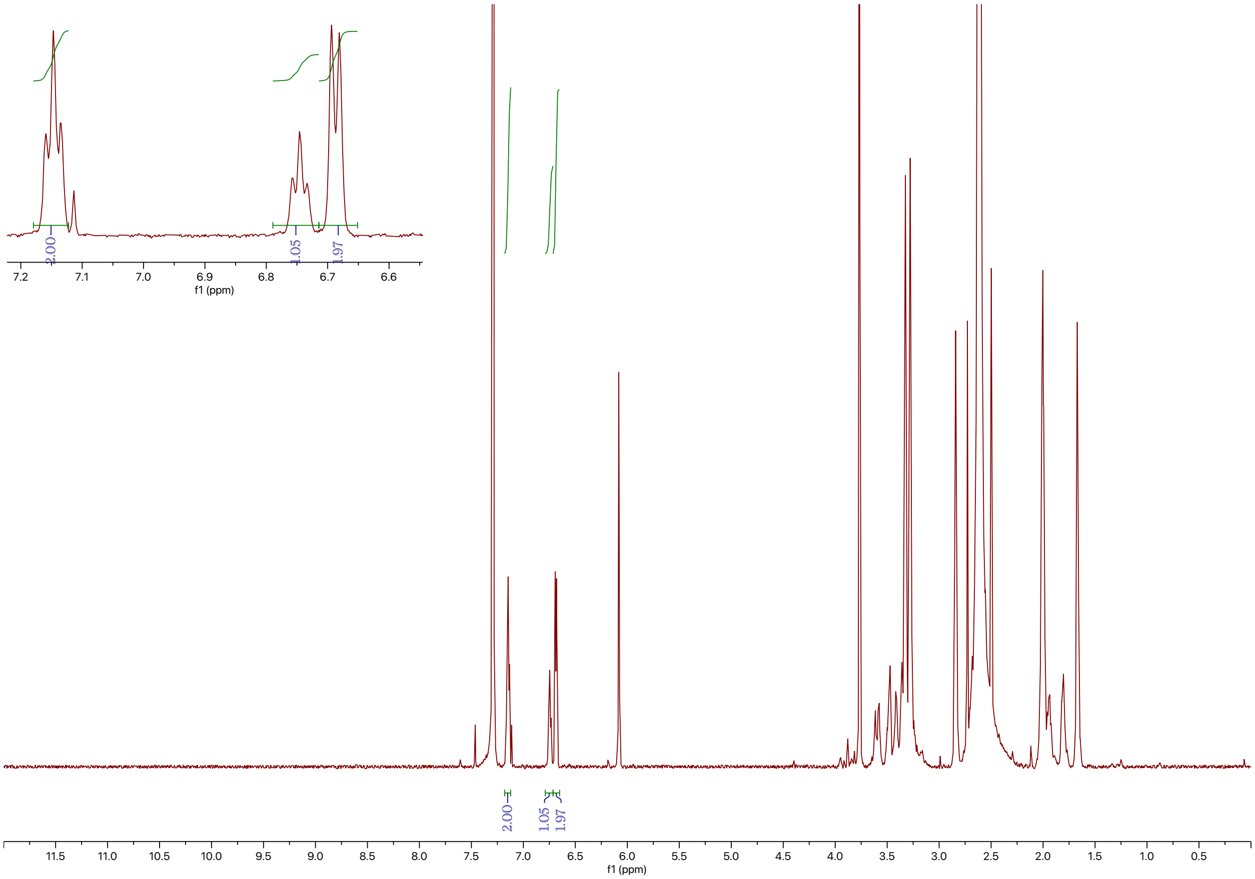

Crude reaction mixture - ^{1}H NMR (600 MHz, $CDCl_3$)

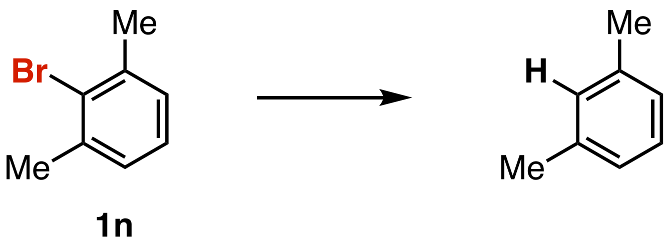

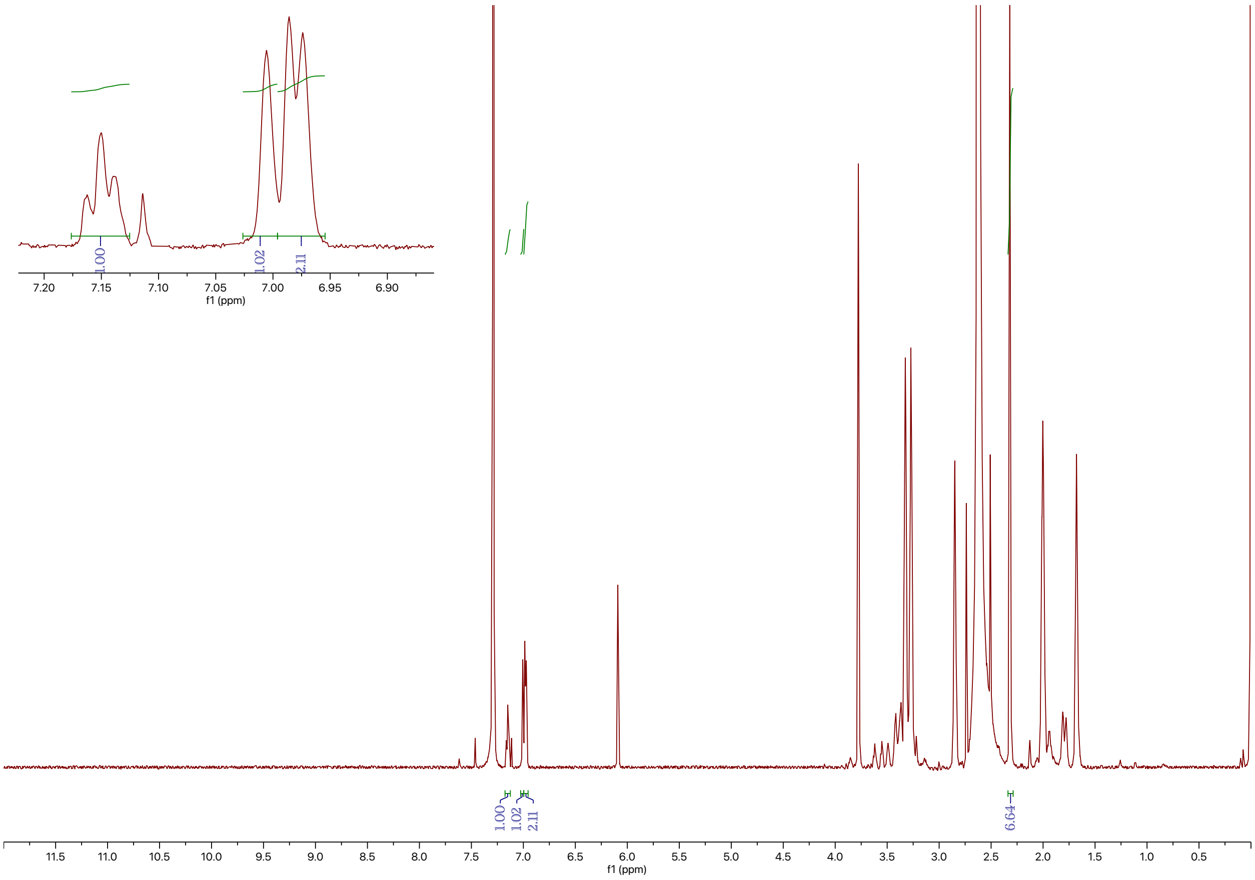

Crude reaction mixture - ^{1}H NMR (600 MHz, $CDCl_3$)

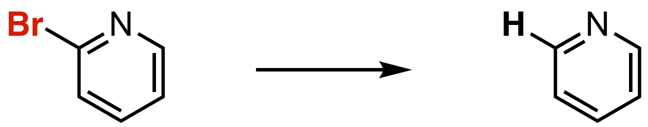

1o

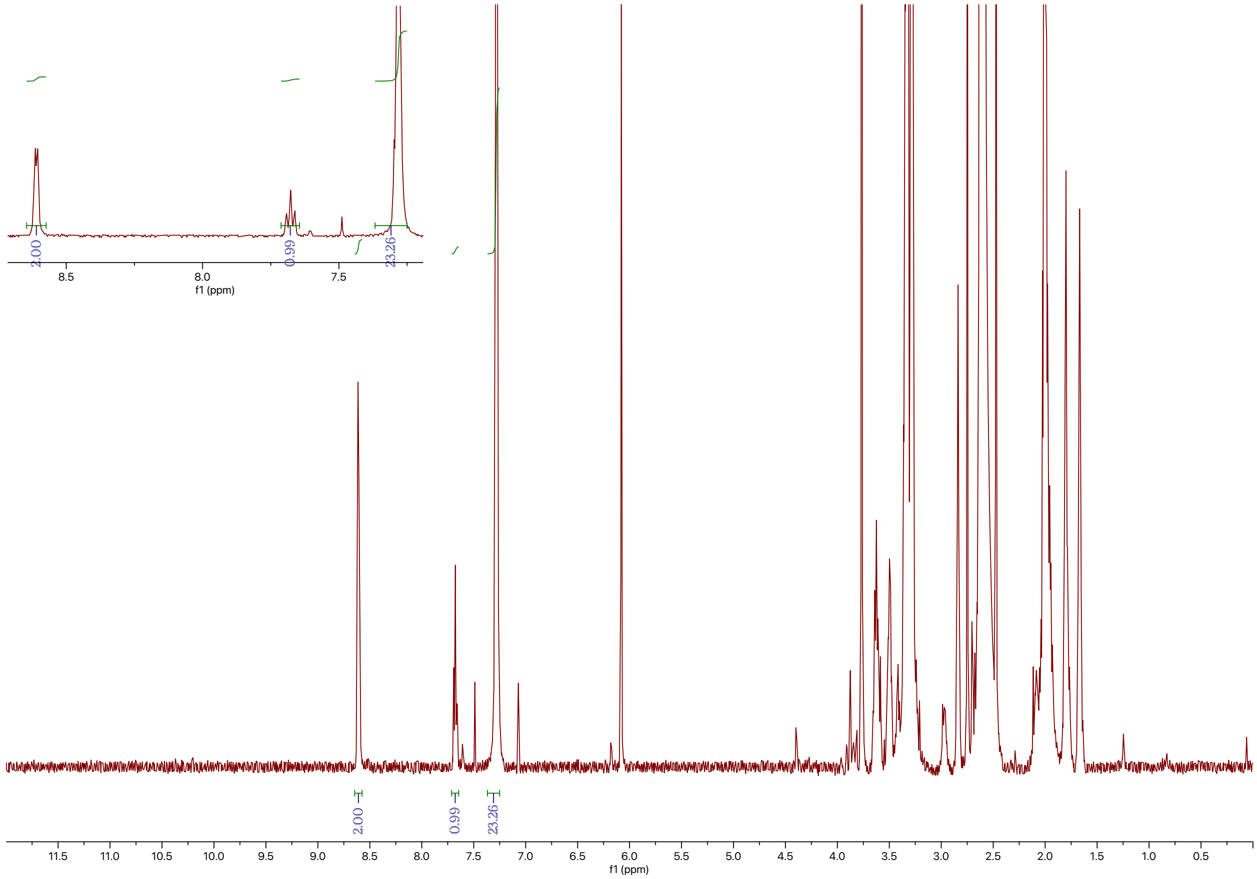

Crude reaction mixture - ^{1}H NMR (600 MHz, $CDCl_3$)

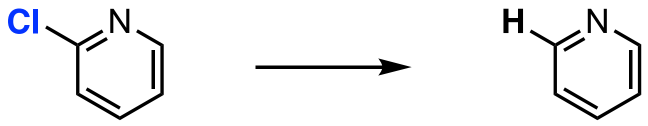

1o'

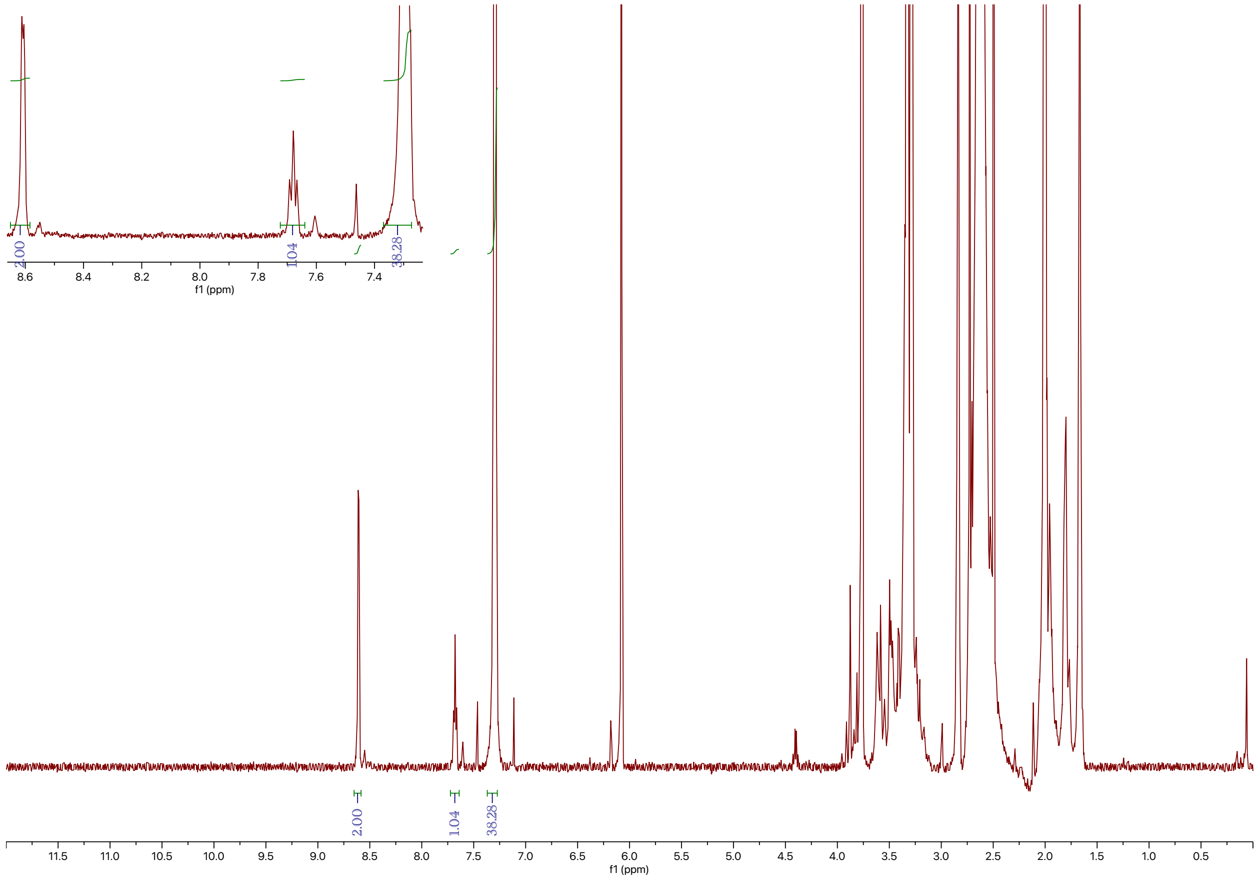

Crude reaction mixture - ^{1}H NMR (600 MHz, $CDCl_3$)

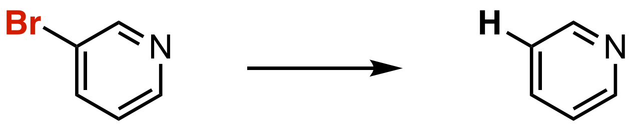

1p

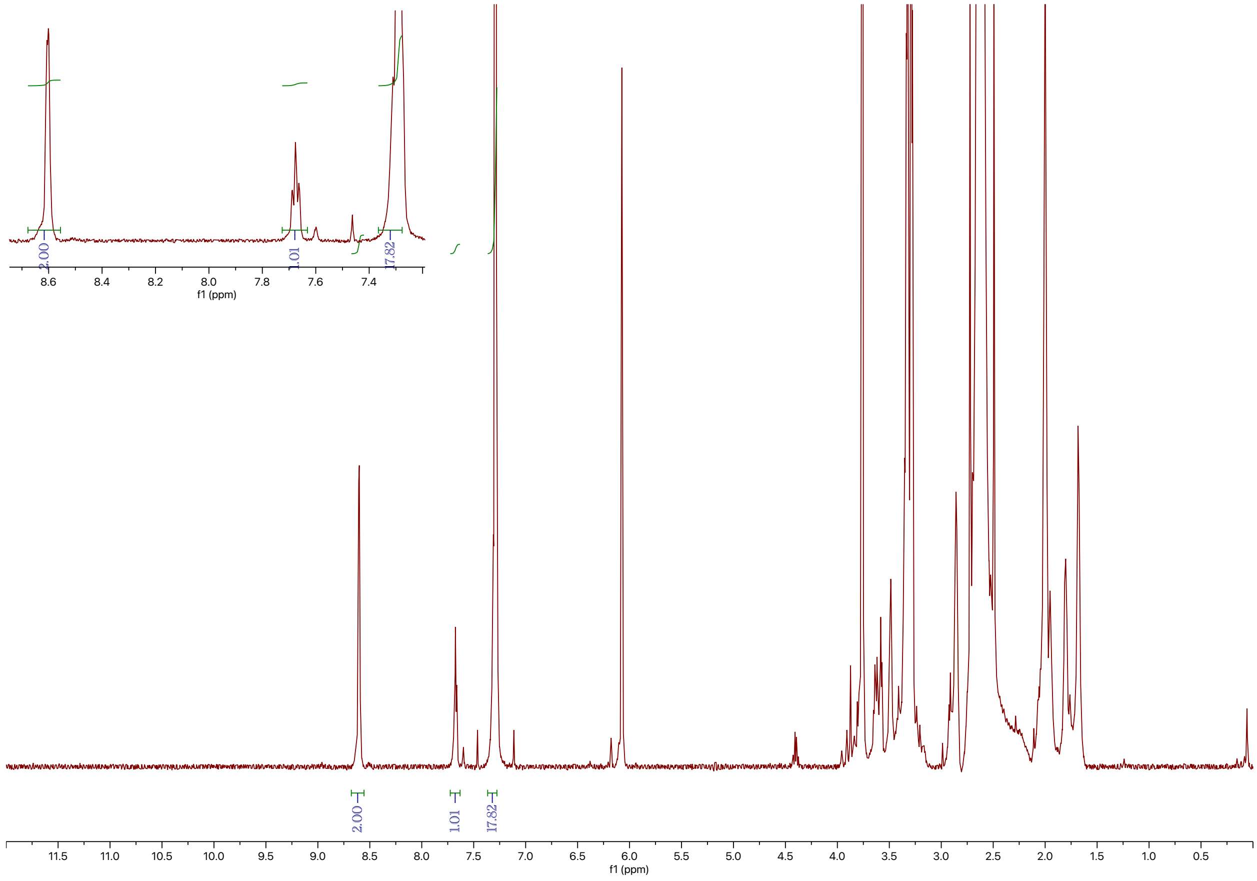

Crude reaction mixture - ^{1}H NMR (600 MHz, $CDCl_3$)

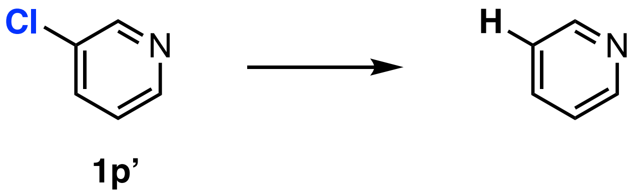

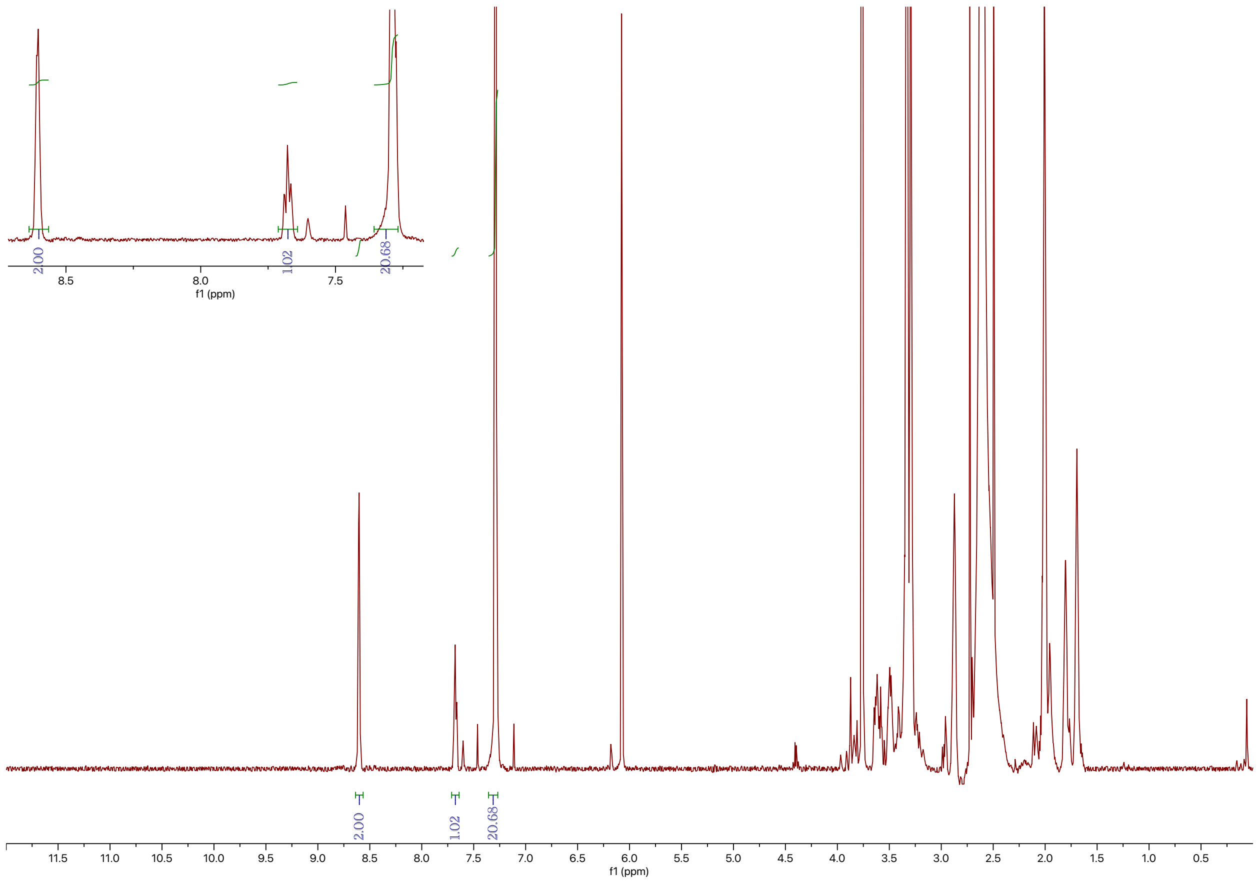

Crude reaction mixture - ^{1}H NMR (600 MHz, $CDCl_3$)

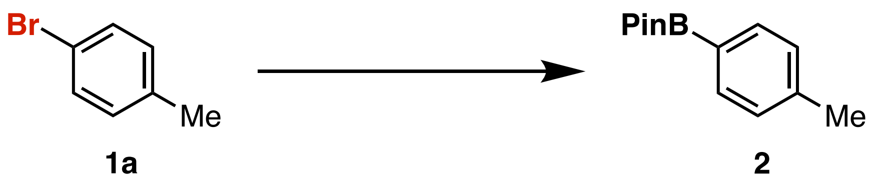

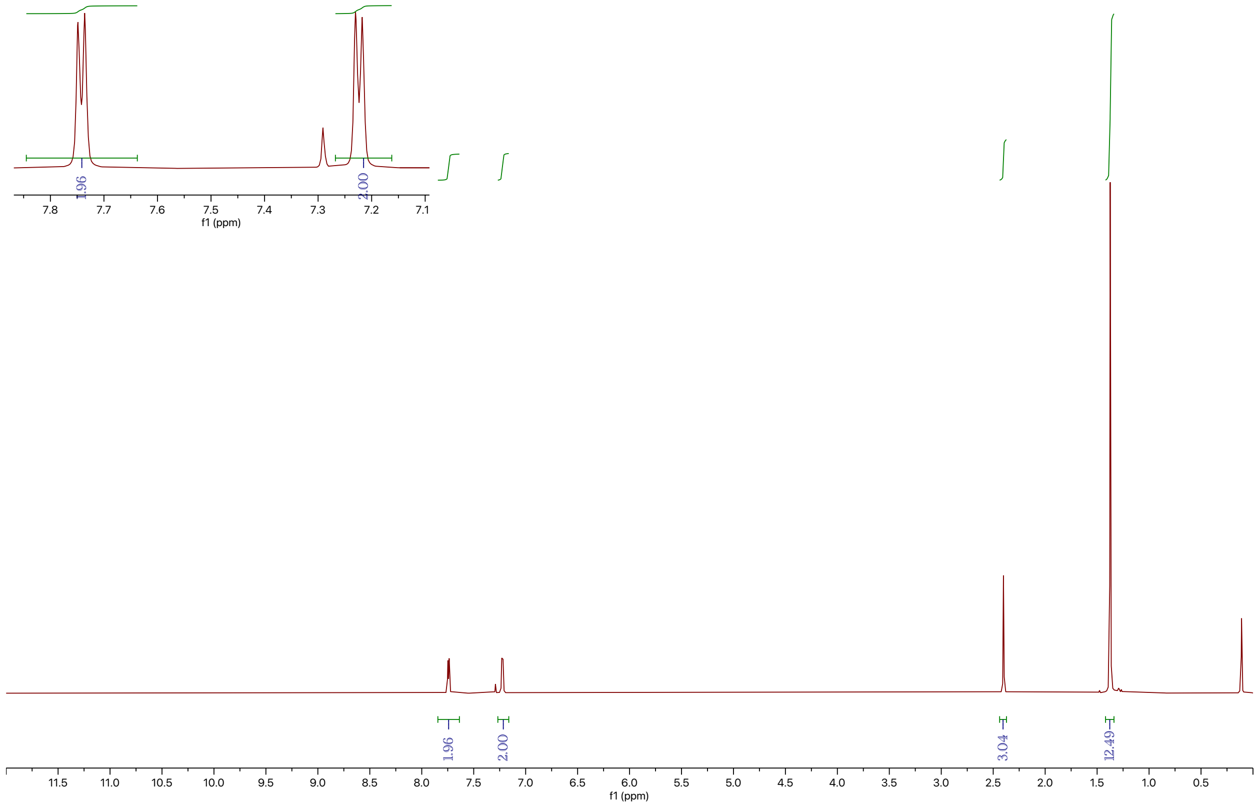

Isolated Product - ^{1}H NMR (600 MHz, $CDCl_3$)

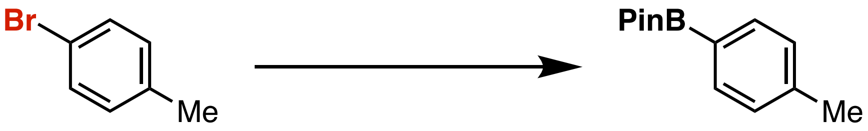

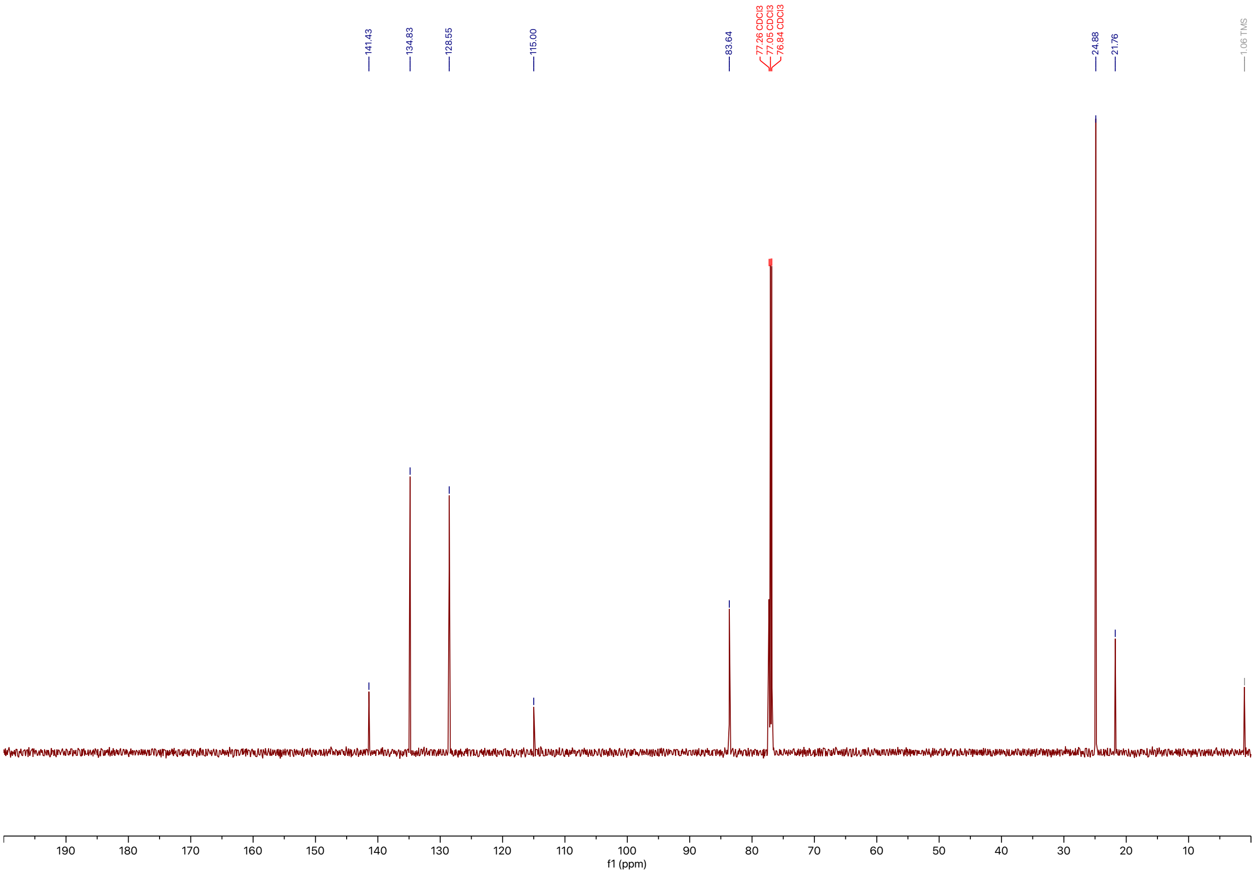

Isolated Product – ^{13}C NMR (151 MHz, $CDCl_3$)

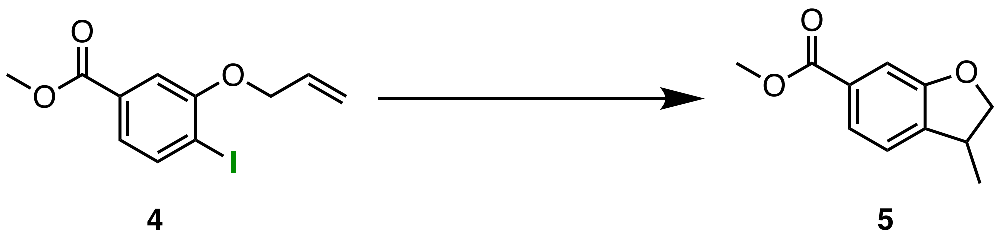

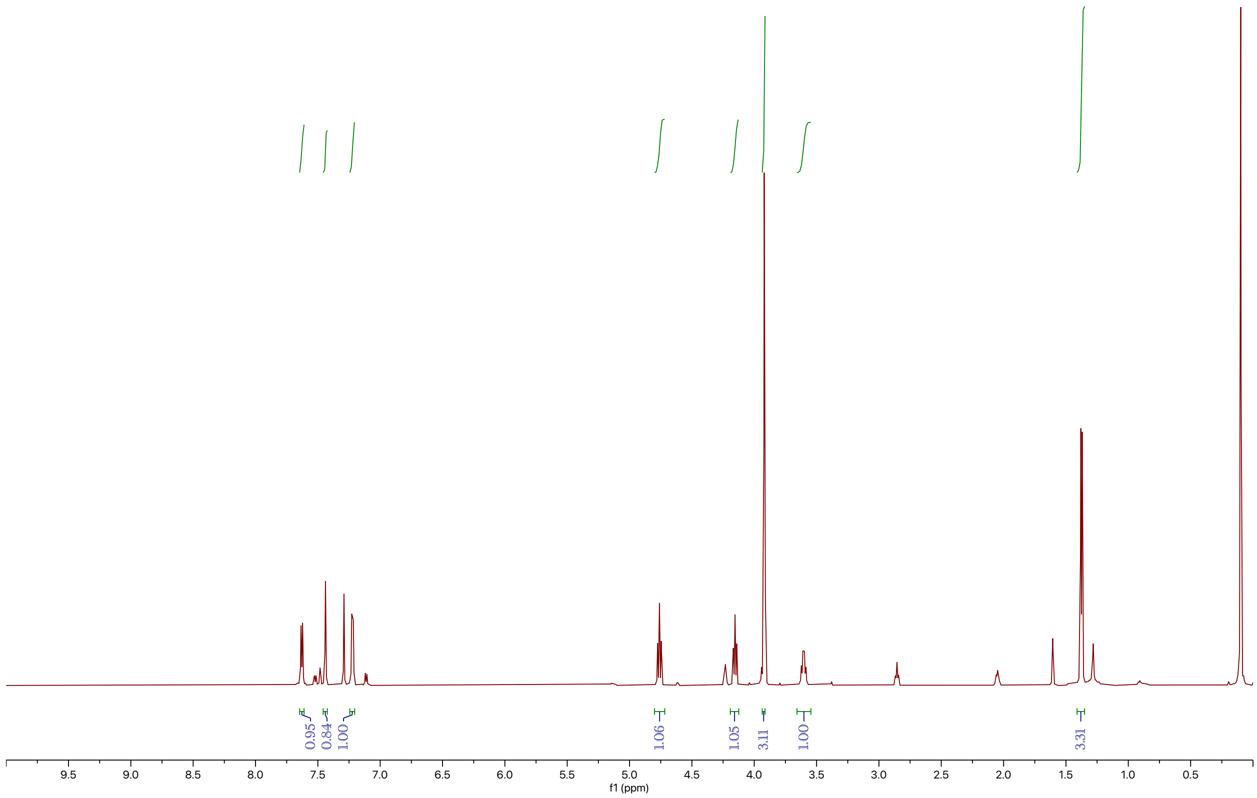

Isolated Product - ^{1}H NMR (600 MHz, $CDCl_3$)

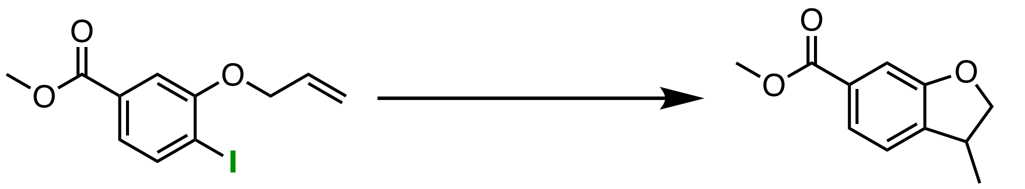

4 **5**

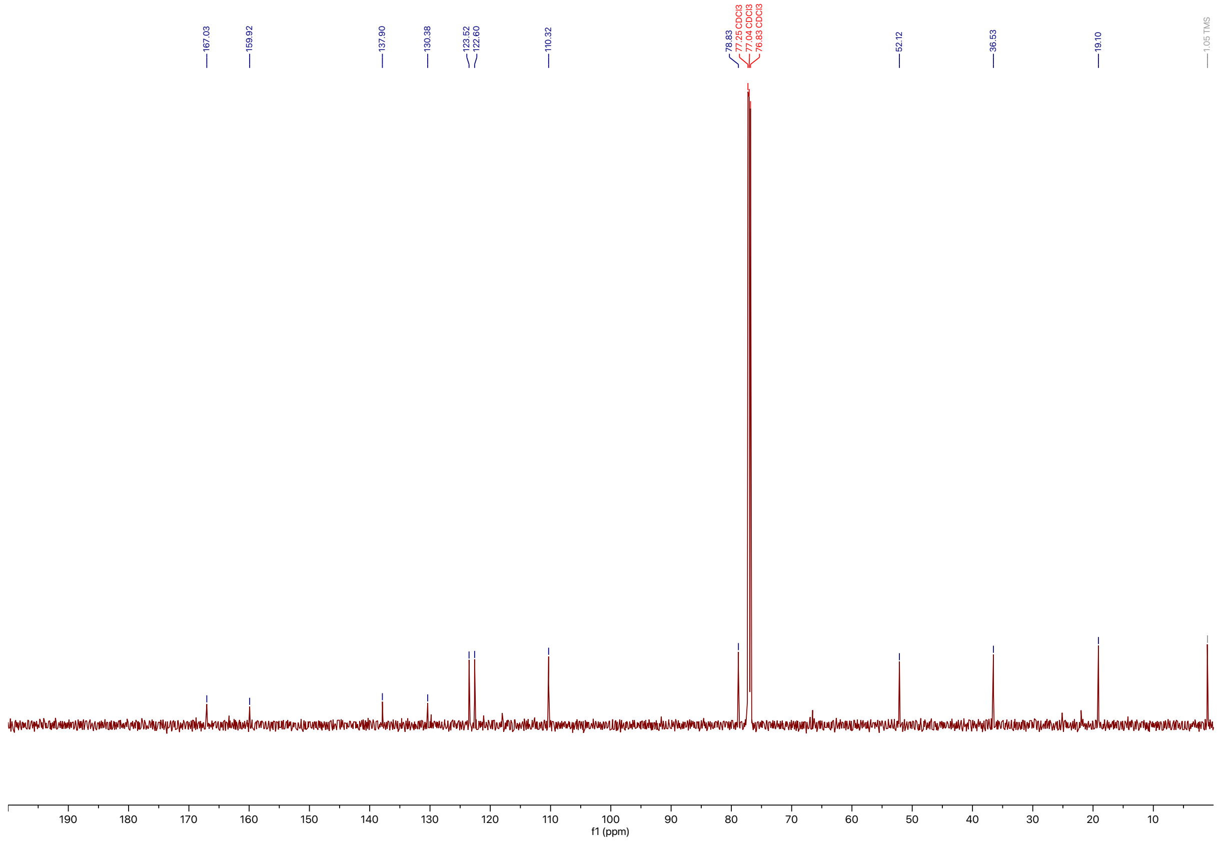

Isolated Product – ^{13}C NMR (151 MHz, $CDCl_3$)

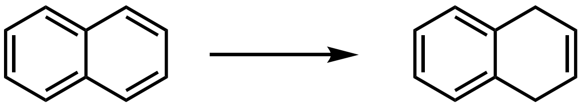

7a

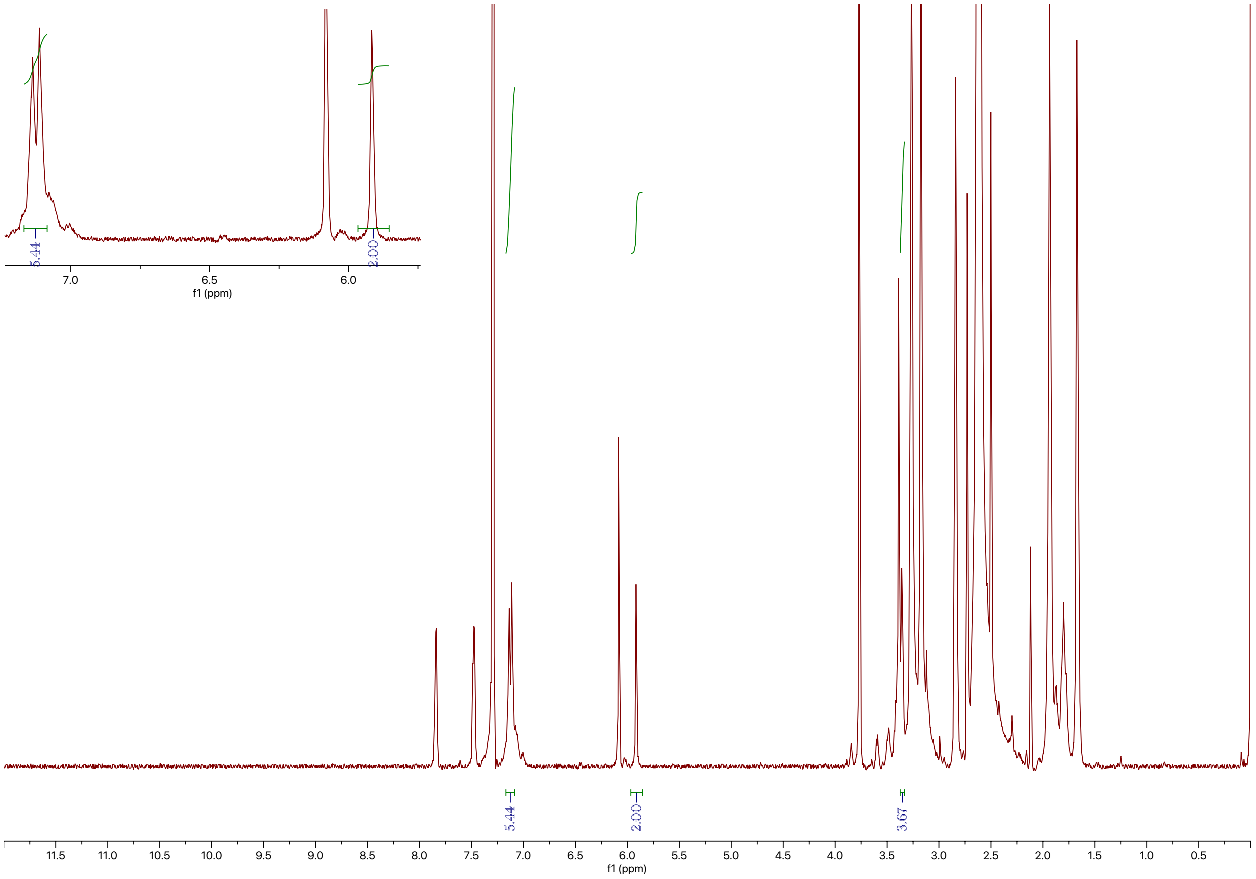

Crude reaction mixture - ^{1}H NMR (600 MHz, $CDCl_3$)

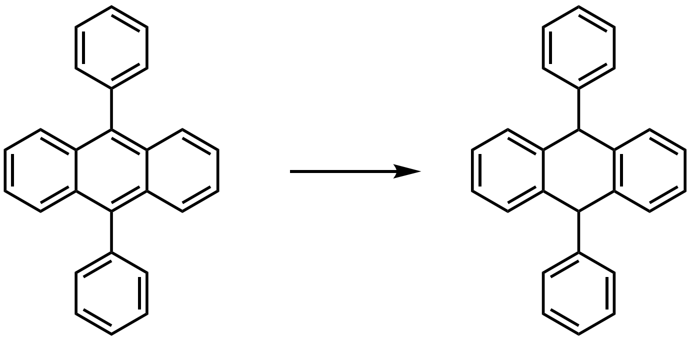

7b

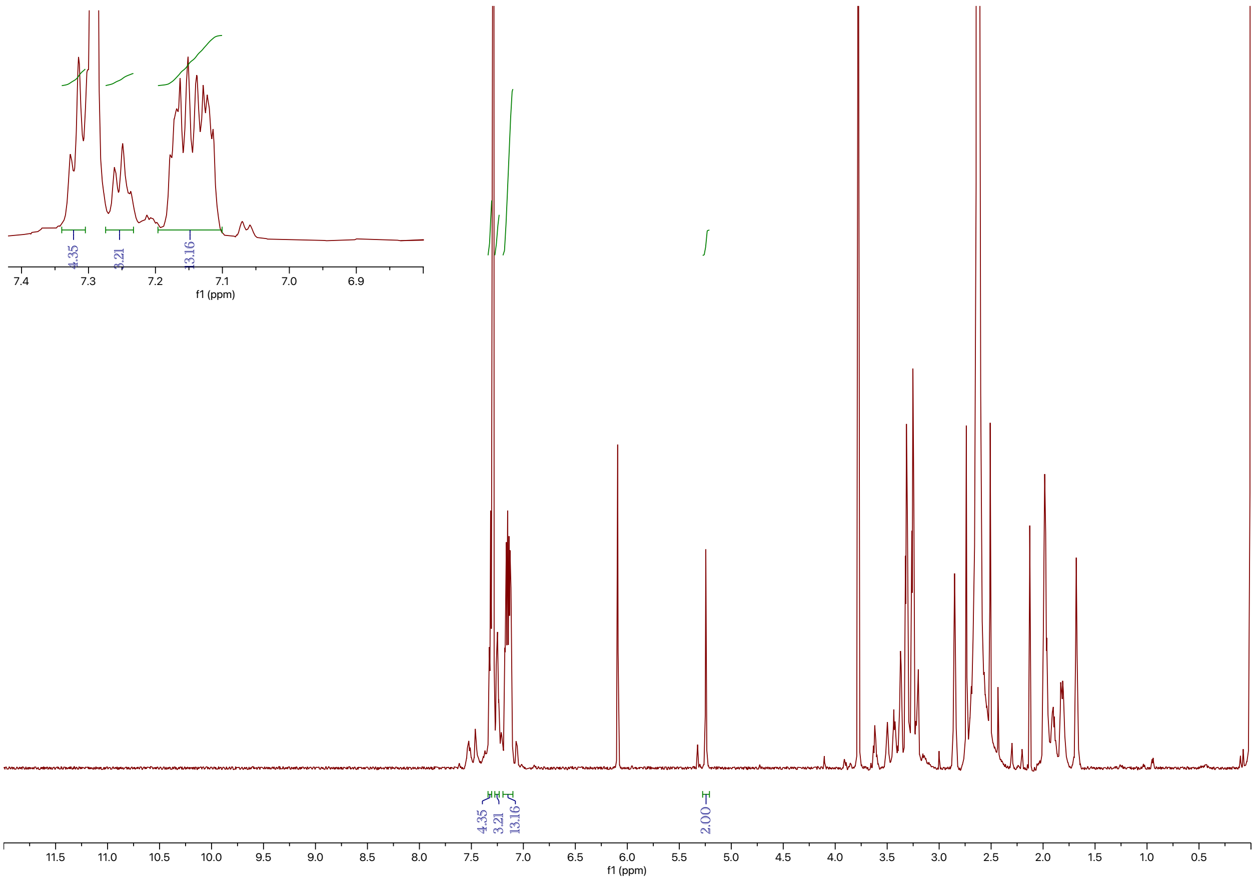

Crude reaction mixture - ^{1}H NMR (600 MHz, $CDCl_3$)
